# Copper-Catalyzed Trifluoromethylation of Alkoxypyridine Derivatives

**DOI:** 10.3390/molecules25204766

**Published:** 2020-10-16

**Authors:** Nandor Gyorfi, Emese Farkas, Norbert Nemet, Csaba Weber, Zoltan Novak, Andras Kotschy

**Affiliations:** 1 Servier Research Institute of Medicinal Chemistry, Záhony u. 7., H-1031 Budapest, Hungary; nandor.gyorfi@servier.com (N.G.); farkas.emese@mail.bme.hu (E.F.); nnorbert141@gmail.com (N.N.); csaba.weber@servier.com (C.W.); 2 Institute of Chemistry, Eötvös Loránd Unversity, Pázmány Péter s. 1/A, H-1117 Budapest, Hungary; novakz@elte.hu

**Keywords:** trifluoromethylation, copper catalysis, heterocycles

## Abstract

The trifluoromethylation of aromatic and heteroaromatic cores has attracted considerable interest in recent years due to its pharmacological relevance. We studied the extension of a simple copper-catalyzed trifluoromethylation protocol to alkoxy-substituted iodopyridines and their benzologs. The trifluoromethylation proceeded smoothly in all cases, and the desired compounds were isolated and characterized. In the trifluoromethylation of 3-iodo-4-methoxyquinoline, we observed a concomitant *O*-*N* methyl migration, resulting in the trifluoromethylated quinolone as a product. Overall, the described procedure should facilitate the broader use of copper-catalyzed trifluoromethylation in medicinal chemistry.

## 1. Introduction

The methyl-methoxypyridine motif is present in several active pharmaceutical ingredients. The most relevant examples include proton pump inhibitors, such as Ilaprazole and Omeprazole [1], the natural antibiotic Piericidin A [2], or a recently developed ERK inhibitor [3]. The incorporation of fluorine into organic molecules typically leads to the improvement of their drug-like properties (i.e., metabolic stability, the crossing of biological barriers) [4,5,6]. This effect is more pronounced when a trifluoromethyl group is introduced, evidenced by the surge in the number of FDA-approved CF_3_-group containing drugs in recent years [7,8,9]. There are multiple approaches for the establishment of the trifluoromethyl group, including nucleophilic, radical, and electrophilic routes [10,11,12,13,14,15,16,17,18,19,20,21,22,23]. Our objective was to systematically study the copper-catalyzed trifluoromethylation [24] of alkoxypyridine derivatives and their benzologs that could serve as useful building blocks in medicinal chemistry.

## 2. Results and Discussion

**Scheme 1:**
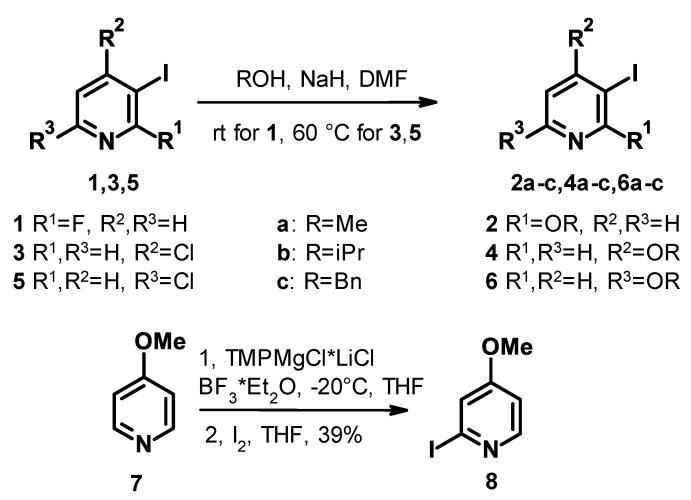
Synthesis of the alkoxy-iodopyridine reagents.

To study the trifluoromethylation of alkoxy-iodopyridines, we needed to synthesize a collection of substrates. The synthesis of such compounds has been described in the literature employing nucleophilic displacement of chlorine or fluorine from a dihalopyridine [25,26,27,28,29]. Following the same logic, we reacted 2-fluoro-3-iodopyridine (**1**), 4-chloro-3-iodopyridine (**3**), or 2-chloro-5-iodopyridine (**5**) with methanol, isopropanol, and benzyl alcohol (Scheme 1) to get the desired alkoxy-iodopyridines (**2a–c**, **4a–c**, **6a–c**). The displacement of the fluorine of **1** proceeded smoothly at ambient temperature, while the transformation of **3** and **5** required elevated temperatures (60 °C). Some of the prepared compounds (**2c**, **4b–c**, **6b–c**) were hitherto unknown. 2-iodo-4-methoxypyridine (**8**) was prepared by the metalation of 4-methoxypyridine, followed by electrophilic quenching of the formed species with I_2_ at −20 °C [30].

**Scheme 2:**
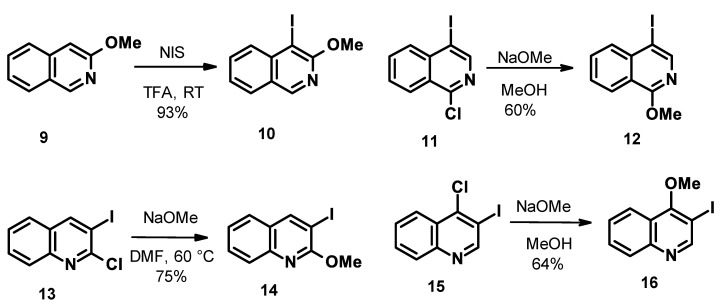
Synthesis of selected methoxy-iodoisoquinoline (**10**, **12**) and quinoline (**14**, **16**) reagents.

We also prepared some bicyclic iodo-methoxypyridine analogs (Scheme 2), namely isoquinolines **10**, **12** and quinolines **14**, **16**. Compounds **12**, **14**, and **16** were prepared analogously to the methoxypyridines, reacting the appropriate chloro-iodo heterocycle (**11**, **13**, **15**) with sodium methoxide. 4-iodo-3-methoxyisoquinoline (**10**), on the other hand, was prepared by the electrophilic iodination of methoxyisoquinoline **9** using *N*-iodosuccinimide, and **10** was isolated in 93% yield.

With the iodo compounds in hand, we studied their reactivity in copper-catalyzed trifluoromethylation reaction, utilizing the literature procedure developed earlier in our laboratories for the functionalization of aryl iodides [24]. These included heating a mixture of one equivalent of the aryl iodide, 20 mol% copper iodide, 20 mol% phenanthroline, three equivalents of potassium fluoride, three equivalents of trimethyl borate, and three equivalents of trimethylsilyl-trifluoromethane under argon in dimethyl sulfoxide at 60 °C. The reactions were usually run on the 2 mmol scale and were monitored by GC-MS and LC. To characterize the efficiency of the transformations, we also determined the conversion of the starting material to the product at the end of the reaction before work-up.

**Scheme 3:**
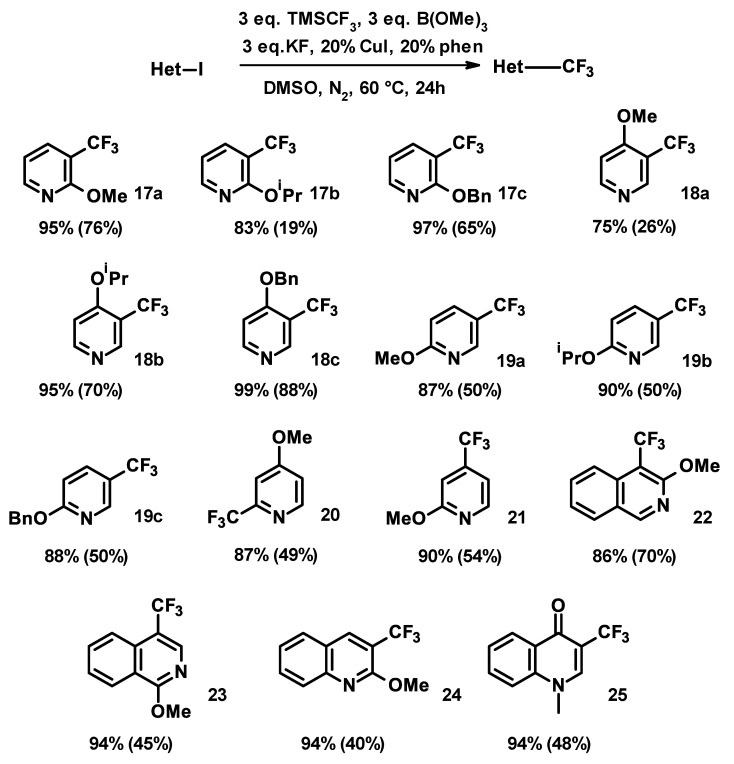
Trifluoromethylation of the alkoxy-iodopyridines, alkoxy-iodoisoquinolines, and alkoxy-iodoquinolines. Numbers in parentheses represent isolated yields.

After the synthesis of heterocyclic iodides, first, we studied the trifluoromethylation of 3-iodopyridine derivatives (Scheme 3). The trifluoromethylation proceeded smoothly, and we observed high conversions in each case. Neither the relative position (c.f. 83%–95%–90% for **17b–18b–19b,** respectively) nor the size of the alkoxy substituent (c.f. 87%–90%–88% for **19a–19b–19c,** respectively) seemed to have a major influence on the transformation. Following an extractive work-up, the products were purified by chromatography. Isolated yields were typically in the 50–88% range, with only two exceptions (**17b**, **18a**). We presumed that this loss was due to product volatility on evaporation of the solvents following the final purification, which was difficult to control at this scale; therefore, we repeated these experiments on a larger scale. On a 4.3 mmol scale, we observed the 90% yield of **17b** by ^19^F NMR measurements in the presence of trifluorotoluene as internal standard, and we could isolate it in a 32% yield. The synthesis of **18a** was repeated on a 7 mmol scale, giving an 83% yield of ^19^F NMR and an isolated yield of 50%. The trifluoromethylation of 2-iodo-4-methoxypyridine (**8**) and 4-iodo-2-methoxypyridine was also smooth (Scheme 3), resulting in high conversions (87% and 90%), and **20** and **21** were isolated in 49% and 54% yield, respectively.

Moving to the benzolog series, the functionalization of 4-iodo-3-methoxyisoquinoline (**10**) and 4-iodo-1-methoxyisoquinoline (**12**) also proceeded smoothly. The trifluoromethylated products **22** and **23** were formed with high conversion and were isolated in 70% and 45% yields, respectively. Switching to the quinoline analogs led to some surprising results. Although the trifluoromethylation of 3-iodo-2-methoxyquinoline (**14**) gave the expected product **24** in 40% yield, starting from 3-iodo-4-methoxyquinoline (**16**), the isolated trifluoromethylated product contained the *N*-methyl-4-quinolone core (**25**) instead of the 4-methoxyquinoline. The structure of **25** was unambiguously proven by NMR measurements. Apparently, the trifluoromethylation was accompanied by the *O*–*N* migration of the methyl group. The *O*–*N* migration of alkoxypyridines is well documented and has been reported to be catalyzed by LiI [31,32], late transition metals, such as Ru and Ir [33,34], or TfOH [35]. It is interesting to note, though, that in the literature precedent, the *O*–*N* methyl migration in 4-methoxyquinoline requires harsher conditions [36]. In our case, we could hypothesize that the combined effect of the iodide and copper ions present in the reaction mixture could facilitate the rearrangement. Following these results, we had a meticulous look at the other reaction mixtures, but we didn’t detect any of the rearranged product in the other reactions.

In summary, we prepared a collection of iodinated methoxypyridines and their benzologs and studied their copper-catalyzed trifluoromethylation. Using the previously optimized coupling conditions, the 15 synthesized iodoarenes were all converted efficiently, and the trifluoromethylated products were usually isolated in good yield. It is worth mentioning that the products were volatile, and their isolated yield might be further improved by increasing the scale of the reaction or using more sophisticated distillation techniques. In the trifluoromethylation of 3-iodo-4-methoxyquinoline, we observed a concomitant *O*–*N* migration of the methyl group. Although this rearrangement is not unprecedented, typically, harsher conditions are required for its completion. Experiments to determine the generality of this phenomenon are in progress in our laboratory.

## 3. Materials and Methods

All commercially available reagents, solvents, and catalysts were used without further purification. Purifications were carried out by forced-flow flash chromatography using pre-packed silica gel cartridges (RediSep Rf Gold) on a Teledyne CombiFlash RF 200 device. Analytical LC-MS: Agilent HP1200 LC with Agilent 6140 quadrupole MS, operating in positive or negative ion electrospray ionization mode. The molecular weight scan range was 100 to 1350 *m/z*. Parallel UV detection was done at 210 nm and 254 nm. LCMS measurements Gemini-NX, 3 μm, C18, 50 mm × 3.00 mm i.d. column at 40 °C, at a flow rate of 1 mL/min using 5 mM aqueous NH_4_HCO_3_ solution and MeCN as eluents. Gas chromatography and low-resolution mass spectrometry were performed on Agilent 6850 gas chromatography and Agilent 5975C mass spectrometer using 15 m × 0.25 mm column with 0.25 μm HP-5MS coating and helium as the carrier gas. Ion source: EI+, 70 eV, 230 °C, quadrupole: 150 °C, interface: 300 °C. UV purity of new compounds is >95% unless otherwise noted. ^1^H and ^13^C NMR spectra were recorded on a Bruker Avance Ultrashield 500 (500 MHz ^1^H and 124 MHz ^13^C) instrument with Bruker Cryo Probe ATM and were internally referenced to residual protio solvent signals (note: DMSO-d_6_ referenced at 2.52 and 39.98 ppm, respectively). ^19^F NMR spectra were recorded on a Bruker Avance III 400 (376.498 MHz ^19^F) instrument with Bruker Prodigy Probe and were referenced to the internal scaling of the instrument. Data for ^1^H NMR were reported as follows: chemical shift (δ ppm), integration, multiplicity (s = singlet, d = doublet, t = triplet, q = quartet, m = multiplet, br = broad), coupling constant (Hz), and assignment. Data for ^13^C NMR were reported in terms of chemical shift, and no special nomenclature was used for equivalent carbons. The ^1^H and ^13^C spectra are available as Supplementary Material. High-resolution mass spectra were obtained on a Shimadzu IT-TOF mass spectrometer system, ion source temperature 200 °C, ESI ±, ionization voltage (±) 4.5 kV, mass resolution min. 10,000. OptiMelt MPA100 melting point apparatus was used for melting point measurements.

3-Iodo-2-methoxypyridine (**2a**) [37], 3-iodo-2-isopropoxypyridine (**2b**) [38], and 3-iodo-4-methoxypyridine (**4a**) [29] were prepared following published procedures.

### 3.1. General Procedure for the Nucleophilic Substitution on 3-Iodopyridine Derivatives

The 0.4 g (10 mmol) of 60% NaH was dissolved in 10 mL dry DMF under an N_2_ atmosphere. The suspension was cooled to 0 °C, and 10 mmol of the appropriate alcohol was added. The solution was allowed to warm up to room temperature. When the formation of H_2_ gas stopped, the solution of 5 mmol of the appropriate 3-iodopyridine (**1**,**3**,**5**) in 15 mL DMF was added, and the mixture was stirred at ambient temperature (for **1**) or at 60 °C (for **3** and **5**) until no further conversion was observed. The mixture was then diluted with water and extracted with EtOAc. The combined organic layers were dried over Na_2_SO_4_, filtered, and concentrated under reduced pressure. The crude product was purified via preparative reversed-phase chromatography using 25 mM aqueous NH_4_HCO_3_ solution and acetonitrile as eluents or via flash chromatography on silica gel using DCM, MeOH, heptane, and EtOAc as eluents.

*2-benzyloxy-3-iodopyridine* (**2c**)

Starting from 1.12 g (5 mmol, 1 equiv.) 2-fluoro-3-iodopyridine (**1**) using the general nucleophilic substitution procedure and 1.0 mL (10 mmol, 2 equiv.) benzyl-alcohol as appropriate starting material, the mixture was stirred at room temperature for 2 h. Following purification of the crude product via flash chromatography on silica gel using heptane and EtOAc (9:1), 2-benzyloxy-3-iodopyridine (**2c**) was obtained as a colorless oil (1.08 g, 69% yield).

^1^H-NMR: (500 MHz, DMSO) δ 8.22 (dd, *J* = 7.5 Hz, *J* = 1.6. Hz, 1H), 8.17 (dd, *J* = 4.8 Hz, *J* = 1.6 Hz, 1H), 7.47 (d, *J* = 7.3 Hz, 2H), 7.40 (t, *J* = 7.5 Hz, 2H), 7.32 (t, *J* = 7.3 Hz, 1H), 6.82 (dd, *J* = 7.5 Hz, *J* = 4.8 Hz, 1H), 5.4 (s, 1H). ^13^C-NMR: (124 MHz, DMSO) δ 161.3, 148.9, 147.0, 137.5, 128.9, 128.4, 127.8, 119.6, 81.2, 68.4. HR-MS: *m/z* calculated for C_12_H_11_INO ([M + H]^+^): 311.9880, found: 311.9879.

*3-iodo-4-isopropoxypyridine* (**4b**)

Starting from 1.20 g (5 mmol) 4-chloro-3-iodopyridine (**3**) using the general nucleophilic substitution procedure and 0.8 mL (10 mmol) isopropyl-alcohol as appropriate starting material, following purification by flash chromatography on silica gel with heptane and ethyl-acetate (7:3), 3-iodo-4-isopropoxypyridine (**4b**) was obtained as a colorless liquid (0.91 g, 69% yield).

^1^H-NMR: (500 MHz, DMSO) δ 8.68 (s, 1H), 8.33 (d, *J* = 5.7 Hz, 1H), 7.08 (d, *J* = 5.7 Hz, 1H), 4.83 (sp, *J* = 6.0 Hz, 1H), 1.32 (d, *J* = 6.0 Hz, 6H). ^13^C-NMR (124 MHz, DMSO) δ 162.6, 157.8, 151.1, 109.9, 87.3, 71.7, 22.0. HR-MS: *m/z* calculated for C_8_H_11_INO ([M + H]^+^): 263.9880, found: 263.9887.

*4-benzyloxy-3-iodopyridine* (**4c**)

Starting from 1.20 g (5 mmol) 4-chloro-3-iodopyridine (**3**) using the general nucleophilic substitution procedure and 1.0 mL (10 mmol) benzyl-alcohol as appropriate starting material, following purification via preparative HPLC on C18 column with ammonium hydrocarbonate and acetonitrile in 5–95% gradient elution, 4-benzyloxy-3-iodopyridine (**4c**) was obtained as light brown oil (1.13 g, 72% yield).

^1^H-NMR: (500 MHz, DMSO) δ 8.72 (s, 1H), 8.38 (d, *J* = 5.6 Hz, 1H), 7.52–7.35 (m, 5H), 7.16 (d, *J* = 5.6 Hz, 1H), 5.31 (s, 2H). ^13^C-NMR (124 MHz, DMSO) δ 163.2, 157.6, 151.3, 136.3, 129.3, 128.6, 127.9, 109.6, 86.5, 70.5. HR-MS: *m/z* calculated for C_12_H_11_INO ([M + H]^+^): 311.9880, found: 311.9890.

*5-iodo-2-isopropoxypyridine* (**6b**)

Starting from 1.20 g (5 mmol) 2-chloro-5-iodopyridine (**5**) using the general nucleophilic substitution procedure and 0.8 mL (10 mmol) isopropyl-alcohol as appropriate starting material, following purification by flash chromatography on silica gel with heptane and ethyl-acetate (9:1), 5-iodo-2-isopropoxypyridine (**6b**) was obtained as a pale brown liquid (1.24 g, 95% yield).

^1^H-NMR (500 MHz, DMSO): δ 8,35 (dd, *J* = 2,4 Hz, *J* = 0.7 Hz, 1H), 7,94 (dd, *J* = 8.6 Hz, *J* = 2,4 Hz, 1H), 6.64 (dd, *J* = 8.7 Hz, *J* = 0.7 Hz, 1H), 5.16 (sp, *J* = 6.2 Hz, 1H), 1,26 (d, *J* = 6.2 Hz, 6H). ^13^C-NMR (124 MHz, DMSO): δ 162.7, 152.8, 147.2, 114.4, 83.3, 68.4, 31.7, 28.9, 22.2, 14.4. HR-MS: *m/z* calculated for C_8_H_11_INO ([M + H]^+^): 263.9880, found: 263.9880.

*2-benzyloxy-5-iodopyridine* (**6c**)

Starting from 1.20 g (5 mmol) 2-chloro-5-iodopyridine (**5**) using the general nucleophilic substitution procedure and 1.0 mL (10 mmol) benzyl-alcohol as appropriate starting material, following purification by preparative HPLC on C18 with ammonium hydrocarbonate and acetonitrile in 5–95% gradient elution, 2-Benzyloxy-5-iodopyridine (**6c**) was obtained as a white solid (1.03 g, 66.1% yield).

^1^H-NMR (500 MHz, DMSO): δ 8.39 (d, *J* = 2.4 Hz, 1H), 8.00 (dd, *J* = 8.7 Hz, *J* = 2.4 Hz, 1H), 7.43–7.30 (m, 5H), 6.79 (d, *J* = 8.7 Hz, 1H), 5.31 (s, 2H). ^13^C-NMR (124 MHz, DMSO): δ 162.9, 152.7, 147.4, 137.3, 128.8, 128.4, 128.3, 114.0, 84.1, 67.6. HR-MS: *m/z* calculated for C_12_H_11_INO ([M+H^+^]): 310.9880, found: 311.9872. Mp: 38.5–39.2 °C

*2-iodo-4 methoxypyridine* (**8**) [30]

A solution of 4-methoxypyridine (2 g, 18.3 mmol) in 90 mL of dry THF containing BF_3_·Et_2_O (2.5 mL, 20 mmol) was treated with TMPMgCl·LiCl (27 mL, 27 mmol, 1M toluene) at −20 °C for 20 h, and the resulting mixture was quenched by adding the solution of iodine (9.14 g, 36 mmol) in 37 mL of THF. Following the warming to room temperature, the resulting mixture was quenched by adding 100 mL of sat. aqueous NH_4_Cl and NH_3_ (9 mL) and sat. aqueous Na_2_S_2_O_3_ solution (18 mL), followed by extraction with Et_2_O (4 × 100 mL). The combined organic layer was dried over MgSO_4_, and the filtrate was concentrated at reduced pressure. The residue was purified on silica gel using heptane and Et_2_O (4:1) as eluent, and **8** was isolated as light brown oil (1.65 g, 39% yield).

^1^H-NMR (500 MHz, DMSO): δ 8.13 (d, *J* = 5.8 Hz, 1H), 7.42 (d, *J* = 2.4 Hz, 1H), 7.01 (dd, *J* = 5.8 Hz, *J* = 2.4 Hz, 1H), 3.82 (s, 3H). ^13^C-NMR (124 MHz, DMSO): δ 166.0, 151.9, 120.3, 119.8, 111.4, 56.3. HR-MS: *m/z* calculated for C_6_H_7_INO ([M+H^+^]): 235.9567, found: 235.9571.

*4-iodo-3-methoxyisoquinoline* (**10**)

In an oven-dried two necked round bottom flask, 3-methoxyisoquinoline (**9**, 508 mg, 3.3 mmol) and *N*-iodosuccinimide (810 mg, 3.5 mmol) were dissolved in dry acetonitrile under nitrogen. The 77 µL trifluoroacetic acid (1 mmol) was added to the mixture by a syringe. The mixture was stirred at room temperature overnight. The solvent was removed, and the residue was treated with 40 mL of water. The aqueous mixture was extracted with 4 × 25 mL of DCM. The organic layer was washed with 25 mL of 1M Na_2_S_2_O_3_ and 50 mL of brine. The organic layer was dried over MgSO_4_. The filtrate was concentrated under reduced pressure to give 4-iodo-3-methoxyisoquinoline (**10**) as off-white solid (870 mg, 93% yield). 

^1^H-NMR (500 MHz, DMSO): δ 9.04 (s, 1H), 8.06 (m, 1H), 7.90 (m, 1H), 7.81 (m, 1H), 7.54 (m, 1H), 4.04 (s, 3H). ^13^C-NMR (124 MHz, DMSO): δ 159.8, 151.4, 140.4, 133.1, 129.3, 129.0, 126.2, 125.7, 78.5, 55.6. HR-MS: *m/z* calculated for C_10_H_9_INO ([M + H]^+^): 285.9723, found: 285.9730. Mp: 67.9–69.5 °C.

*4-iodo-1-methoxyisoquinoline* (**12**)

NaOMe (112 mg, 2.07 mmol) was dissolved in 5 mL dry MeOH under an N_2_ atmosphere. 4-iodo-1-chloroisoquinoline (**11**, 400 mg, 1.38 mmol), dissolved in 5 mL dry dioxane, was added to it, and the mixture was heated to 80 °C for 8 h. The solvents were removed in vacuo, and the residue was diluted with water and extracted with EtOAc. The combined organic layers were washed with brine, dried over MgSO_4_, filtered, and concentrated under reduced pressure to give **12** as a light brown solid (236 mg, 60% yield).

^1^H-NMR (500 MHz, DMSO): δ 8.43 (s, 1H), 8.18 (m, *J* = 8.2 Hz, 1H), 7.91 (m, 1H), 7.89 (m, 1H), 7.73 (m, *J* = 8.2 Hz, 1H), 4.06 (s, 3H). ^13^C-NMR (124 MHz, DMSO): δ 161.2, 147.3, 138.0, 133.1, 130.4, 128.9, 124.7, 120.6, 87.6, 54.5. HR-MS: *m/z* calculated for C_10_H_9_INO ([M + H]^+^): 285.9723, found: 285.9727. Mp: 43.9–44.5 °C.

*3-iodo-2-methoxyquinoline* (**14**)

An oven-dried vial was charged with 3-iodo-2-chloroquinoline (**13**, 200 mg, 0.7 mmol), dissolved in dry DMF (5 mL). The 25 w% NaOMe solution in MeOH (190 µL, 179 mg) was added slowly to the mixture by a syringe under nitrogen. The mixture was stirred for 3 h at 40 °C. The mixture was quenched with 2 mL of water. Purification via preparative HPLC on C18 column with ammonium hydrocarbonate and acetonitrile, in 5–95% gradient elution, with direct injection gave **14** as light-brown solid (148 mg, 75% yield).

^1^H-NMR (500 MHz, DMSO): δ 8.86 (s, 1H), 7.86 (m, 1H), 7.79 (m, 1H), 7.70 (m, 1H), 7.46 (m, 1H), 4.02 (s, 3H). ^13^C-NMR (124 MHz, DMSO): δ 159.2, 148.8, 145.6, 130.8, 127.3, 127.0, 126.9, 125.2, 82.4, 55.2. HR-MS: *m/z* calculated for C_10_H_9_INO ([M + H]^+^): 285.9723, found 285.9717. Mp: 46.8–47.9 °C.

*3-iodo-4-methoxyquinoline* (**16**)

NaOMe (169.8 mg, 3.14 mmol) was dissolved in 4 mL of dry CH_3_OH under an N_2_ atmosphere. 3-iodo-4-chloroquinoline (**15**, 700 mg, 2.42 mmol), dissolved in 8 mL of dry dioxane, was added to it, and the mixture was heated to 80 °C for 6 h. The methanol was then removed in vacuo, and the residue was diluted with water and extracted with EtOAc. The combined organic layers were washed with brine, dried over MgSO_4_, filtered, and concentrated under reduced pressure. The crude product was purified via flash chromatography using heptane and EtOAc as eluents to give **16** as light yellow solid (440 mg, 64% yield).

^1^H-NMR (500 MHz, DMSO): δ 9.08 (s, 1H), 8.12 (dm, *J* = 8.4 Hz, 1H), 8.05 (dm, *J* = 8.4 Hz, 1H), 7.84 (m, *J* = 8.4 Hz, 1H), 7.69 (m, *J* = 8.4 Hz, 1H), 4.02 (s, 3H). ^13^C-NMR (124 MHz, DMSO): δ 163.8, 158.0, 149.4, 130.9, 129.6, 127.9, 124.5, 122.4, 86.3, 62.4. HR-MS: *m/z* calculated for C_10_H_9_INO ([M + H]^+^): 285.9723, found: 285.9726. Mp: 122.3–124.9 °C.

### 3.2. General Trifluoromethylation Procedure

An oven-dried vial with a septum cap and a stir bar was charged with copper (I) iodide (76 mg, 0.4 mmol), 1,10-phenanthroline (72 mg, 0.4 mmol), KF (348 mg, 6 mmol), and the aryl iodide (2.00 mmol, if solid). The reaction vessel was closed, then evacuated and refilled with argon or nitrogen three times. DMSO (4.0 mL), aryl iodide (2.00 mmol, if liquid), B(OMe)_3_ (623 mg, 6 mmol), and TMSCF_3_ (854 mg, 887 μL, 6 mmol) were added via syringe. The resulting orange-brown suspension was stirred for 24 h at 60 °C. After cooling to ambient temperature, the orange solution was diluted with DCM (10 mL) and washed with 1N HCl (25 mL). Acidic washing was omitted for basic products. The washing was re-extracted with DCM (2 × 5 mL), and the combined organic layer was washed with conc. ammonia (25%, 25 mL) to remove traces of copper salts. The washing was re-extracted with DCM (2 × 5 mL), and the combined organic layer was washed with brine (15 mL) and dried over MgSO_4_ and concentrated. The crude product was purified by flash column chromatography unless otherwise noted.

*2-methoxy-3-trifluoromethylpyridine* (**17a**)

Starting from **2a** (2.01 g, 8.5 mmol), using the general trifluoromethylation procedure, **17a** was obtained as a colorless liquid (1.14 g, 76% yield). No further purification was done after the extraction.

^1^H-NMR (500 MHz, DMSO): δ 8.46 (dd, *J* = 4.7 Hz, *J* = 1.0 Hz, 1H), 8.07 (dd, *J* = 7.5 Hz, 1H), 7.18 (m, *J* = 6.9 Hz, *J* = 5.1 Hz, 1H), 3.98 (s, 3H). ^13^C-NMR (124 MHz, DMSO): δ 160.5, 151.7, 137.5, 123.6, 117.2, 111.9, 54.4. ^19^F-NMR (376 MHz, DMSO): δ −62.4. HR-MS: *m/z* calculated for C_7_H_7_F_3_NO ([M + H]^+^): 178.0474, found: 178.0473.

*2-isopropoxy-3-trifluoromethylpyridine* (**17b**)

Starting from **2b** (528 mg, 2 mmol), using the general trifluoromethylation procedure, **17b** was obtained following the purification by flash chromatography on silica gel with heptane and ethyl-acetate (1:1) as a colorless liquid (77 mg, 19% yield).

^1^H-NMR (500 MHz, DMSO): δ 8.42 (m, 1H), 8.07 (m, 1H), 7.13 (m, 1H), 5.39 (sp, *J* = 6.2 Hz, 1H), 1.31 (d, *J* = 6.2 Hz, 6H). ^13^C-NMR (124 MHz, DMSO): δ 160.0, 151.8, 137.6, 123.6, 116.8, 112.3, 69.6, 22.1. ^19^F-NMR (376 MHz, DMSO): δ -62.4. HR-MS (GC-MS (TOF); [M]^+^): Calculated for C_9_H_10_F_3_NO([M]^+^): 205.0714, found: 205.0703.

*2-benzyloxy-3-trifluoromethylpyridine* (**17c**)

Starting from **2c** (237 mg, 0.75 mmol), using the general trifluoromethylation procedure following the purification via preparative HPLC on C18 column with ammonium hydrocarbonate and acetonitrile in 5–95% gradient elution, **17c** was obtained as a colorless liquid (123 mg, 65% yield).

^1^H-NMR: (500 MHz, DMSO): δ 8.45 (dd, *J* = 5.0 Hz, *J* = 1.0 Hz, 1H), 8.14 (dd, *J* = 7.6 Hz, *J* = 1.1 Hz, 1H), 7.45–7.30 (m, 5H), 7.20 (m, 1H), 5.51 (s, 2H). ^13^C-NMR: (124 MHz, DMSO): δ 159.9, 151.8, 137.8, 128.9, 128.3, 127.8, 124.7, 123.6, 117.6, 112.1, 68.0. ^19^F-NMR (376 MHz, DMSO): δ −62.3. HR-MS: *m/z* calculated for C_13_H_11_F_3_NO ([M + H]^+^): 254.0787, found: 254.0795.

*4-methoxy-3-trifluoromethylpyridine* (**18a**)

Starting from **4a** (473 mg, 2 mmol), using the general trifluoromethylation procedure following the purification by flash chromatography on silica gel with pentane and ethyl-acetate (1:2), **18a** was obtained as a colorless liquid (93 mg, 26% yield).

^1^H-NMR (500 MHz, DMSO): δ 8.72 (d, *J* = 5.9 Hz, 1H), 8.68 (s, 1H), 7.34 (d, *J* = 5.9 Hz, 1H), 3.98 (s, 3H). ^13^C-NMR (500 MHz, DMSO): δ 163.6, 156.2, 147.7, 123.8, 114.1 108.9, 57.1. ^19^F-NMR (376 MHz, DMSO) δ -61.0. HR-MS: *m/z* calculated for C_7_H_7_F_3_NO ([M + H]^+^): 178.0474, found: 178.0475.

*4-isopropoxy-3-trifluoromethylpyridine* (**18b**)

Starting from **4b** (1.00 g, 3.8 mmol), using the general trifluoromethylation procedure following the purification by flash chromatography on silica gel with pentane and diethyl ether (1:1), **18b** was obtained as a colorless liquid (670 mg, 85% yield).

^1^H-NMR (500 MHz, DMSO): δ 8.66 (s, 1H), 8.65 (s, 1H), 7.35 (d, *J* = 6.0 Hz, 1H), 4.94 (sp, *J* = 6.1 Hz, 1H), 1.31 (d, *J* = 6.1 Hz, 6H). ^13^C-NMR (124 MHz, DMSO): δ 162.2, 155.8, 148.0, 123.8, 114.7, 110.0, 72.2, 22.5. ^19^F-NMR (376 MHz, DMSO-*d*_6_): δ -61.2. HR-MS: *m/z* calculated for C_9_H_11_F_3_NO ([M + H]^+^): 206.0793, found: 206.0788.

*4-benzyloxy-3-trifluoromethylpyridine* (**18c**)

Starting from **4c** (235 mg, 0.75 mmol), using the general trifluoromethylation procedure following the purification via preparative HPLC on C18 column with ammonium hydrocarbonate and acetonitrile in 5–95% gradient elution, **18c** was obtained as an off-white solid (167 mg, 88% yield). 

^1^H-NMR (500 MHz, DMSO): δ 8.72 (m, 1H), 8.71 (m, 1H), 7.43 (m, 5H), 7.36 (m, 1H), 5.39 (s, 2H). ^13^C-NMR (124 MHz, DMSO): δ 162.6, 156.1, 147.9, 135.9, 129.1, 128.7, 127.9, 123.8, 109.9, 70.6. ^19^F-NMR (376 MHz, DMSO): δ -61.0. HRMS: *m/z* calculated for C_13_H_11_F_3_NO ([M + H]^+^): 254.0793, found: 254.0788. Mp: 66.0–68.6 °C.

*2-methoxy-5-trifluoromethylpyridine* (**19a**)

Starting from **6a** (1.39 g, 5.9 mmol), using the general trifluoromethylation procedure following the purification by flash chromatography on silica gel with heptane and DCM, **19a** was obtained as a colorless liquid (822 mg, 70% yield).

^1^H-NMR (500 MHz, DMSO): δ 8,60 (m, 1H), 8,07 (dd, *J* = 8.7 Hz, *J* = 2,6 Hz, 1H), 7,03 (d, *J* = 8.7 Hz, 1H), 3,93 (s, 3H). ^13^C-NMR (124 MHz, DMSO): δ 166.4, 145.4, 136.9, 111.8, 54.4. ^19^F-NMR (376 MHz, DMSO): δ -59.9. HR-MS: *m/z* calculated for C_7_H_7_F_3_NO ([M + H]^+^): 178.0474, found: 178.0474.

*2-isopropoxy-5-trifluoromethylpyridine* (**19b**)

Starting from **6b** (770 mg, 3.0 mmol), using the general trifluoromethylation procedure following the purification by flash chromatography on silica gel with heptane and ethyl-acetate (9:1), **19b** was obtained as a colorless liquid (340 mg, 52% yield).

^1^H-NMR (500 MHz, DMSO): δ 8.57 (m, 1H), 8.02 (d, *J* = 8.8 Hz, *J* = 2.7 Hz, 1H), 6.93 (d, *J* = 8.8 Hz, 1H), 5.32 (sp, *J* = 6.2 Hz, 1H), 1.31 (d, *J* = 6.2 Hz, 6H). ^13^C-NMR (500 MHz, DMSO): δ 165.7, 145.4, 136.8, 112.2, 69.3, 22.1. ^19^F-NMR (376 MHz, DMSO): δ -59.8. HR-MS (GC-MS, TOF, EI): calculated for C_9_H_10_F_3_NO ([M]^+^): 205.0714, found: 205.0705.

*2-benzyloxy-5-trifluoromethylpyridine* (**19c**)

Starting from **6c** (466 mg, 1.5 mmol), using the general trifluoromethylation procedure following the purification via preparative HPLC on C18 column with ammonium hydrocarbonate and acetonitrile in 5–95% gradient elution, **19c** was obtained as a colorless liquid (192 mg, 51% yield).

^1^H-NMR (500 MHz, DMSO): δ 8.61 (m, 1H), 8.10 (dd, *J* = 8.8 Hz, *J* = 2.6 Hz, 1H), 7.47–7.32 (m, 5H), 7.09 (d, *J* = 8.8 Hz, 1H), 5.43 (s, 2H). ^13^C-NMR (124 MHz, DMSO): δ 165.8, 145.4, 137.1, 137.0, 128.9, 128.6, 128.5, 124.6, 119.5, 112.1, 68.3. ^19^F-NMR (376 MHz, DMSO): δ -59.9. HR-MS: *m/z* calculated for C_13_H_11_F_3_NO ([M + H]^+^): 254.0793, found: 254.0789.

*4-methoxy-2-trifluoromethylpyridine* (**20**)

Starting from **8** (1.18 g, 5 mmol), using the general trifluoromethylation procedure following the purification by flash chromatography on silica gel with pentane and diethyl-ether (3:1), **20** was obtained as a colorless liquid (700 mg, 49% yield).

^1^H-NMR (500 MHz, DMSO): δ 8.57 (d, *J* = 5.7 Hz, 1H), 7.44 (d, *J* = 2.5 Hz, 1H), 7.28 (dd, *J* = 5.7 Hz, *J* = 2.6 Hz, 1H), 3.93 (s, 3H). ^13^C-NMR (124 MHz, DMSO): δ 166.9, 152.0, 148.6, 122.0, 113.3, 108.1, 56.6. ^19^F-NMR (376 MHz, DMSO): δ -66.6. HR-MS: *m/z* calculated for C_7_H_7_F_3_NO ([M + H]^+^): 178.0474, found: 178.0486.

*2-methoxy-4-trifluoromethylpyridine* (**21**)

Starting from 4-iodo-2-methoxypyridine (1.17 g, 5.0 mmol), using the general trifluoromethylation procedure following the purification by flash chromatography on silica gel with pentane and diethyl-ether (95:5), **21** was obtained as a colorless liquid (478 mg, 54% yield).

^1^H-NMR (500 MHz, DMSO): δ 8.45 (d, *J* = 5.3 Hz, 1H), 7.33 (dd, *J* = 5.3 Hz, *J* = 0.8 Hz, 1H), 7.20 (m, 1H), 3.92 (s, 3H). ^13^C-NMR (124 MHz, DMSO): δ 164.5, 149.5, 140.1, 123.2, 112.8, 107.6, 54.4. ^19^F-NMR (376 MHz, DMSO): δ -63.5. HR-MS: *m/z* calculated for C_7_H_7_F_3_NO ([M + H]^+^): 178.0474, found: 178.0481.

*3-methoxy-4-trifluoromethylisoquinoline* (**22**)

Starting from **10** (150 mg, 0.5 mmol), using the general trifluoromethylation procedure following the purification via preparative HPLC on C18 column with ammonium hydrocarbonate and acetonitrile in 5–95% gradient elution, **22** was obtained as a white solid (80 mg, 72% yield).

^1^H-NMR (500 MHz; DMSO): δ 9.38 (s, 1H), 8.22 (d, *J* = 8.0 Hz, 1H), 8.07 (d, J = 8.6 Hz, 1H), 7.91–7.87 (m, 1H), 7.63–7.59 (m, 1H), 4.09 (s, 3H). ^13^C-NMR (124 MHz; DMSO): δ 158.7, 156.7, 135.0, 133.8, 129.9, 125.7, 125.7, 125.0, 122.2, 100.6, 55.2. ^19^F-NMR (376 MHz): δ -52.7 HR-MS: *m/z* calculated for C_11_H_9_F_3_NO ([M + H]^+^): 228.0632, found: 228.0637. Mp: 63.2–65.3 °C.

*1-methoxy-4-trifluoromethylisoquinoline* (**23**)

Starting from **12** (300 mg, 1.1 mmol), using the general trifluoromethylation procedure following the purification via preparative HPLC on C18 column with ammonium hydrocarbonate and acetonitrile in 5–95% gradient elution, **23** was obtained as a white solid (115 mg, 46% yield).

^1^H-NMR (500 MHz, DMSO): δ 8.49 (m, 1H). 8.34 (m, 1H), 7.99 (m, 2H), 7.81 (m, 1H), 4.15 (s, 3H). ^13^C-NMR (124 MHz, DMSO): δ 163.7, 140.1, 133.4, 132.9, 129.0, 125.1, 123.0, 119.0, 113.9, 55.1. ^19^F-NMR (376 MHz, DMSO): δ -58.7. HR-MS: *m/z* calculated for C_11_H_9_F_3_NO ([M+H]^+^): 228.0631, found: 228.0632. Mp: 56.9–58.4 °C.

*2-methoxy-3-trifluoromethylquinoline* (**24**)

Starting from **14** (73 mg, 0.2 mmol), using the general trifluoromethylation procedure following the purification via preparative HPLC on C18 column with ammonium hydrocarbonate and acetonitrile in 5–95% gradient elution, **24** was obtained as a light-brown oil (18 mg, 40% yield).

^1^H-NMR (500 MHz, DMSO): δ 8.82 (s, 1H), 8.09 (d, 1H), 7.86 (m, 2H), 7.57 (m, 1H), 4.01 (s, 3H). ^13^C-NMR (124 MHz, DMSO): δ 157.7, 147.5, 139.4, 133.0, 129.7, 127.0, 125.9, 124.1, 54.6. ^19^F-NMR (376 MHz, DMSO): δ -62.2. HR-MS: *m/z* calculated for C_11_H_8_F_3_NO (GC-MS (TOF); [M]^+^): 227.0558, found: 227.0549.

*1-methyl-3-trifluoromethyl-4-quinolone* (**25**)

Starting from **16** (285 mg, 1 mmol), using the general trifluoromethylation procedure following the purification via preparative HPLC on PFP column with formic acid and acetonitrile in 56% isocratic elution, **25** was obtained as white crystals (112 mg, 48% yield).

^1^H-NMR (500 MHz): δ 8.59 (s, 1H), 8.24 (m, *J* = 8.0 Hz, 1H), 7.89–7.85 (m, *J* = 8.5 Hz, 1H), 7.77 (m, 1H, *J* = 8.5 Hz), 7.56–7.52 (m, *J* = 8.0 Hz, 1H), 3.93 (s, 3H). ^13^C-NMR (124 MHz): δ 173.0, 145.2, 140.9, 133.7, 127.2, 126.0, 125.5, 117.9, 108.7, 41.1. ^19^F-NMR (376 MHz): δ -61.4. HR-MS: *m/z* calculated for C_11_H_9_F_3_NO ([M + H]^+^): 228.0632, found: 228.0627. Mp: 169.5–179.3 °C.

## Supplementary Materials

The following are available online, NMR spectra of the synthesized compounds.
