# Supplementary material for: Copper-Catalyzed Trifluoromethylation of Alkoxypyridine Derivatives
Source: Molecules. 2020 Oct 16;25(20):4766. doi: 10.3390/molecules25204766 (PMC7587554; doi:10.3390/molecules25204766)
Supplement: Supplementary file 1 [file molecules-25-04766-s001.pdf]

Supporting Information

Copper catalysed trifluoromethylation of alkoxypyridine derivates

Nándor Győrfi [1], Emese Farkas [1], Norbert Német [1], Csaba Wéber [1], Zoltán Novák [2] and András Kotschy [1,*]

[1] Servier Research Institute of Medicinal Chemistry, Záhony u. 7., H-1031 Budapest, Hungary
[2] Institute of Chemistry, Eötvös Loránd Unversity, Pázmány Péter s. 1/A, H-1117 Budapest, Hungary
* Correspondence: andras.kotschy@servier.com

Content

The ^{1}H, ^{13}C and ^{19}F NMR spectra of the prepared compounds

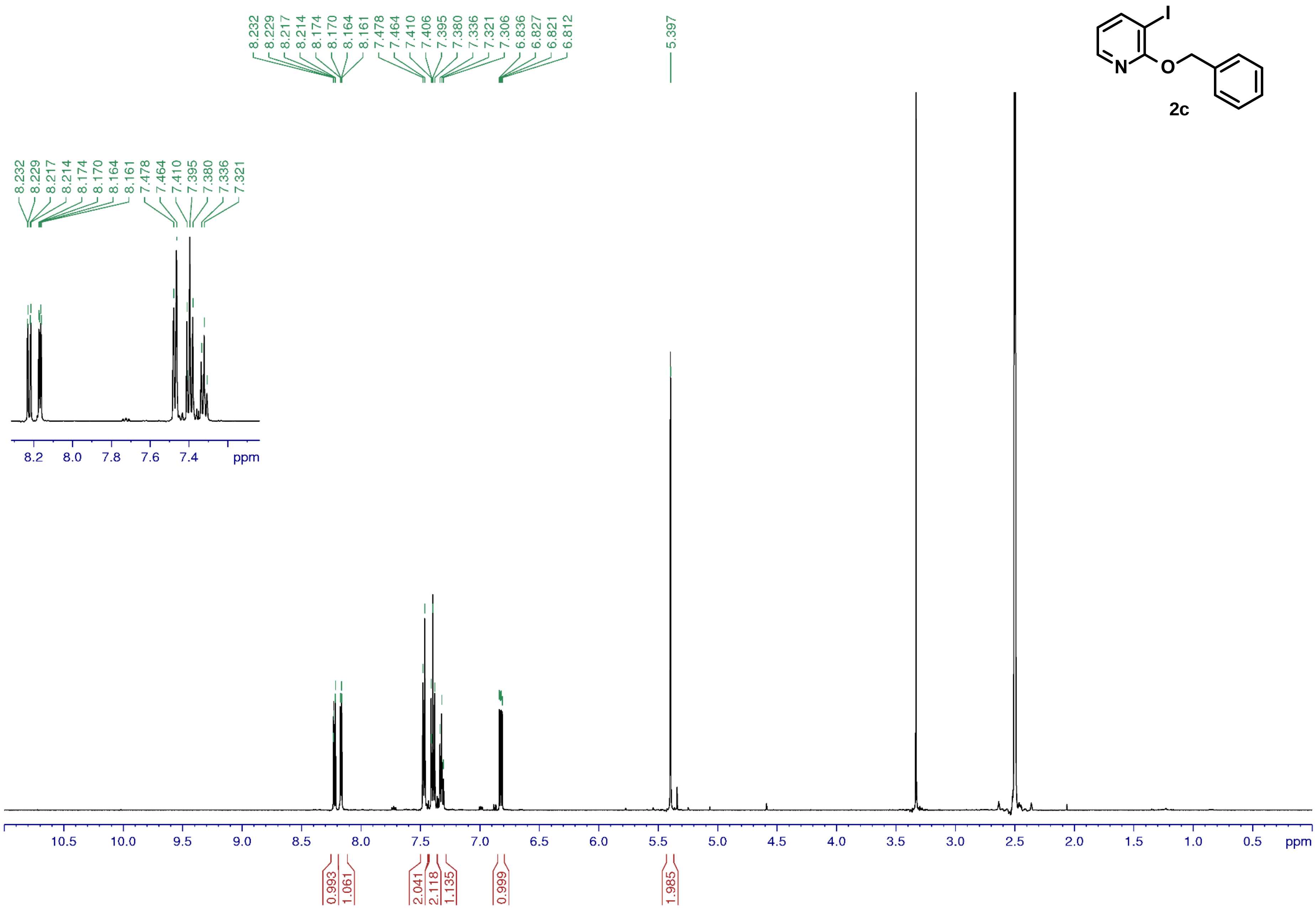

8.232
8.229
8.217
8.214
8.174
8.170
8.164
8.161
7.478
7.464
7.410
7.406
7.395
7.380
7.336
7.321
7.306
6.836
6.827
6.821
6.812
5.397
8.2 8.0 7.8 7.6 7.4 ppm
10.5 10.0 9.5 9.0 8.5 8.0 7.5 7.0 6.5 6.0 5.5 5.0 4.5 4.0 3.5 3.0 2.5 2.0 1.5 1.0 0.5 ppm
0.993
1.061
2.041
2.118
1.135
0.999
1.985

2c

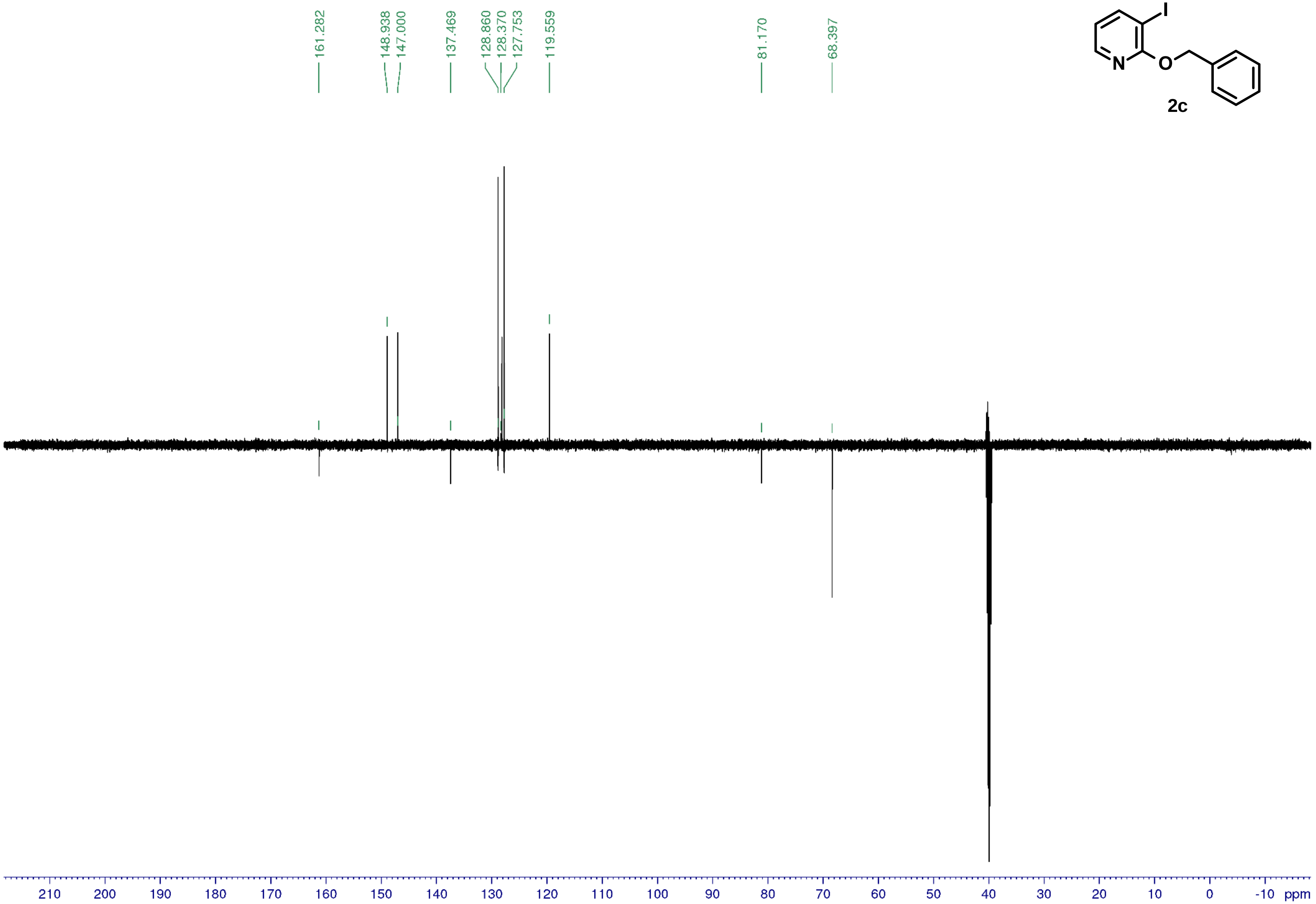
161.282
148.938
147.000
137.469
128.860
128.370
127.753
119.559
81.170
68.397
I
N
O
2c
210 200 190 180 170 160 150 140 130 120 110 100 90 80 70 60 50 40 30 20 10 0 -10 ppm

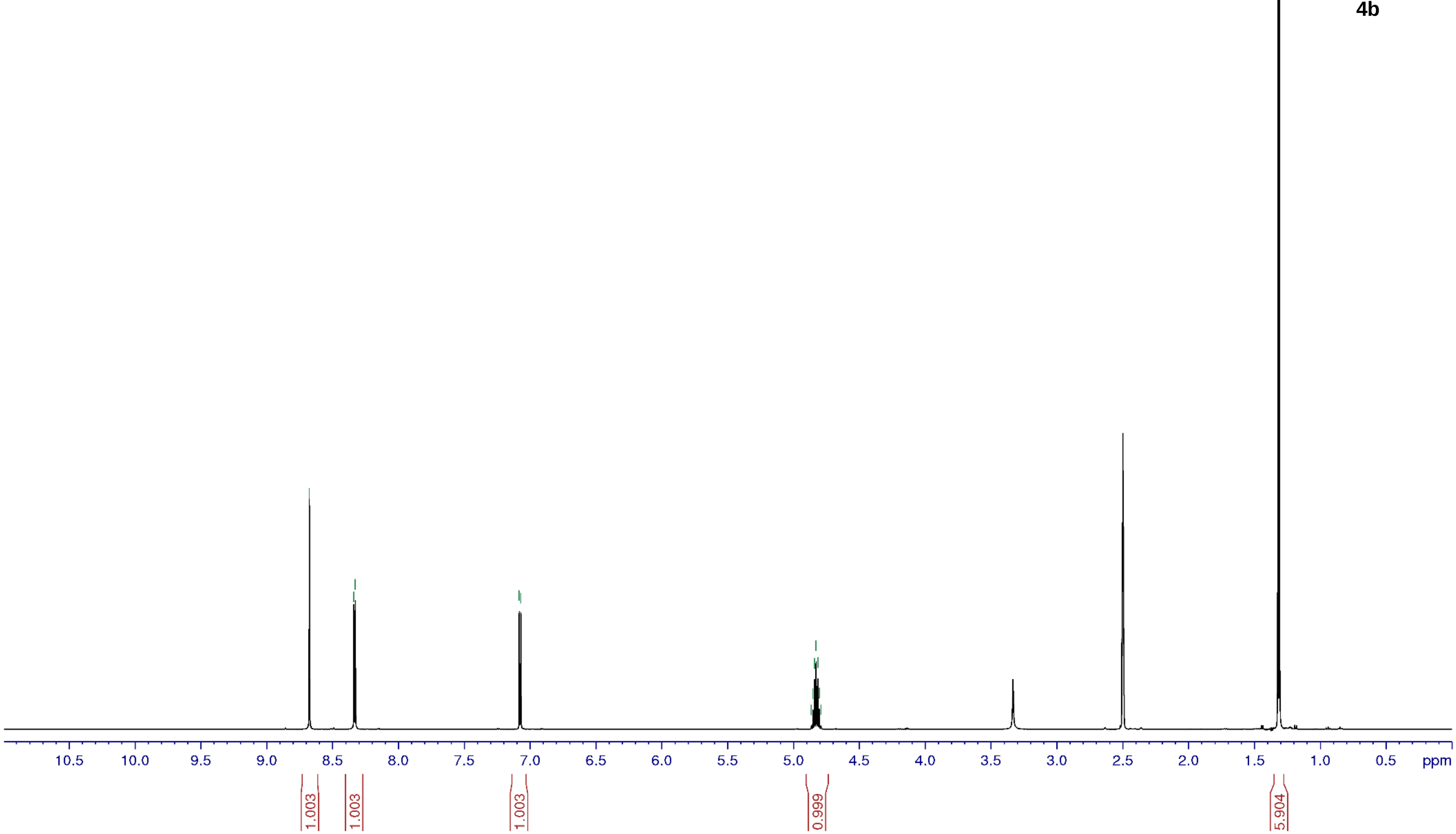
8.678
8.339
8.328
7.084
7.072
4.865
4.853
4.841
4.829
4.817
4.805
4.793
1.323
1.311
10.5 10.0 9.5 9.0 8.5 8.0 7.5 7.0 6.5 6.0 5.5 5.0 4.5 4.0 3.5 3.0 2.5 2.0 1.5 1.0 0.5 ppm
1.003
1.003
1.003
0.999
5.904

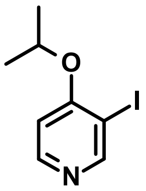
O
I
N

4b

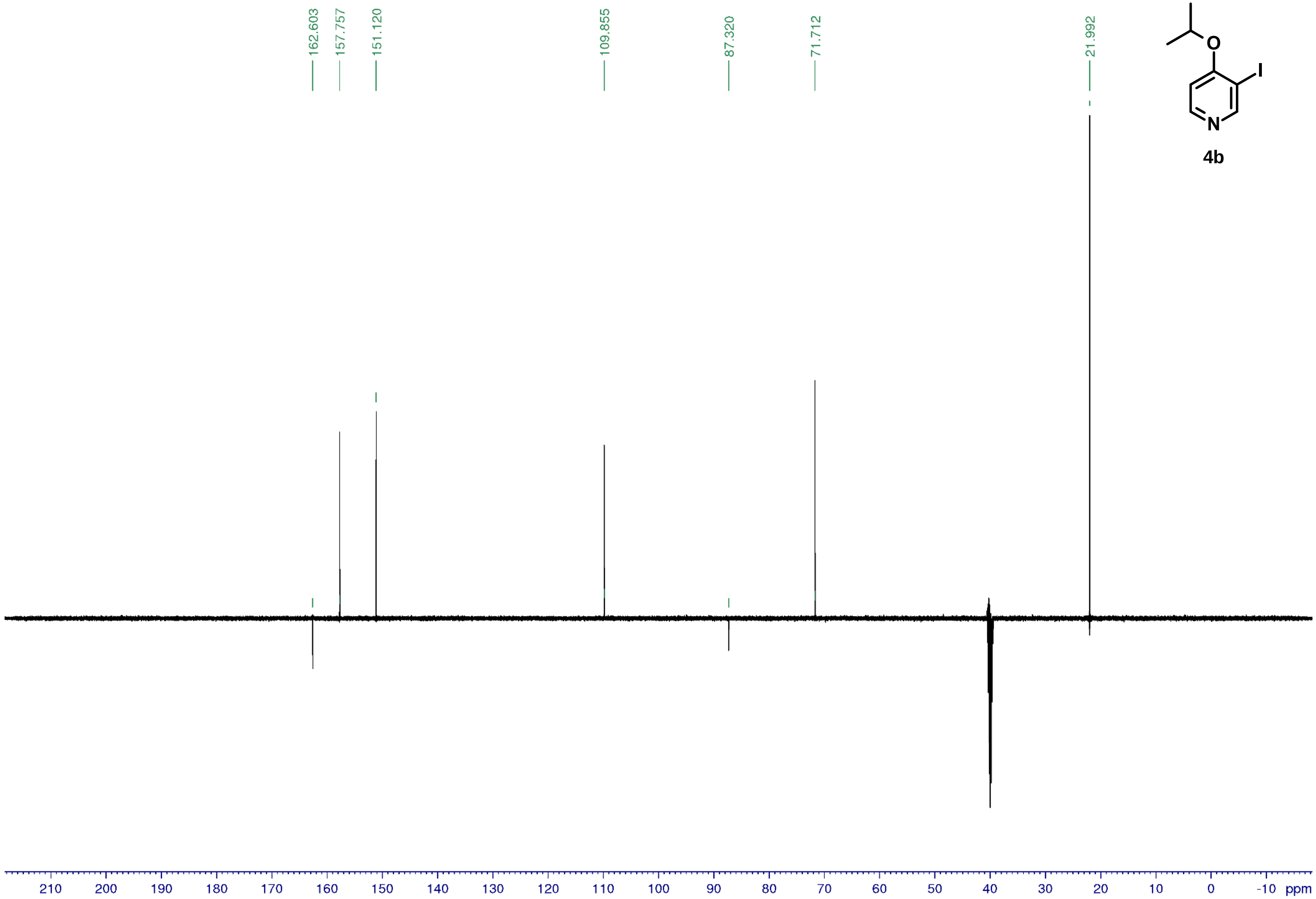
162.603
157.757
151.120
109.855
87.320
71.712
21.992
O
I
N
4b
210
200
190
180
170
160
150
140
130
120
110
100
90
80
70
60
50
40
30
20
10
0
-10
ppm

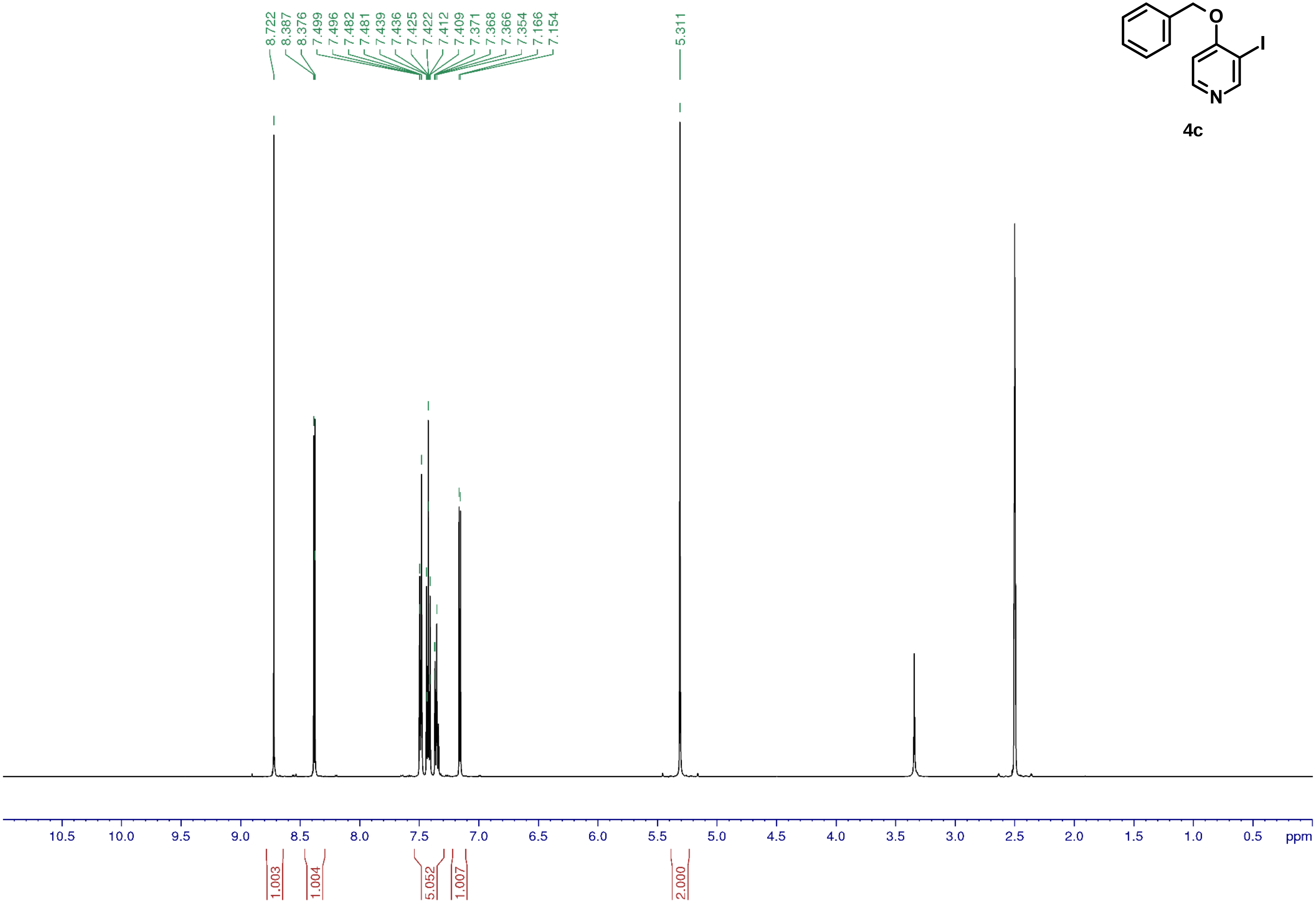
8.722
8.387
8.376
7.499
7.496
7.482
7.481
7.439
7.436
7.425
7.422
7.412
7.409
7.371
7.368
7.366
7.354
7.166
7.154
5.311
O
I
N
10.5
10.0
9.5
9.0
8.5
8.0
7.5
7.0
6.5
6.0
5.5
5.0
4.5
4.0
3.5
3.0
2.5
2.0
1.5
1.0
0.5
ppm
1.003
1.004
5.052
1.007
2.000

4c

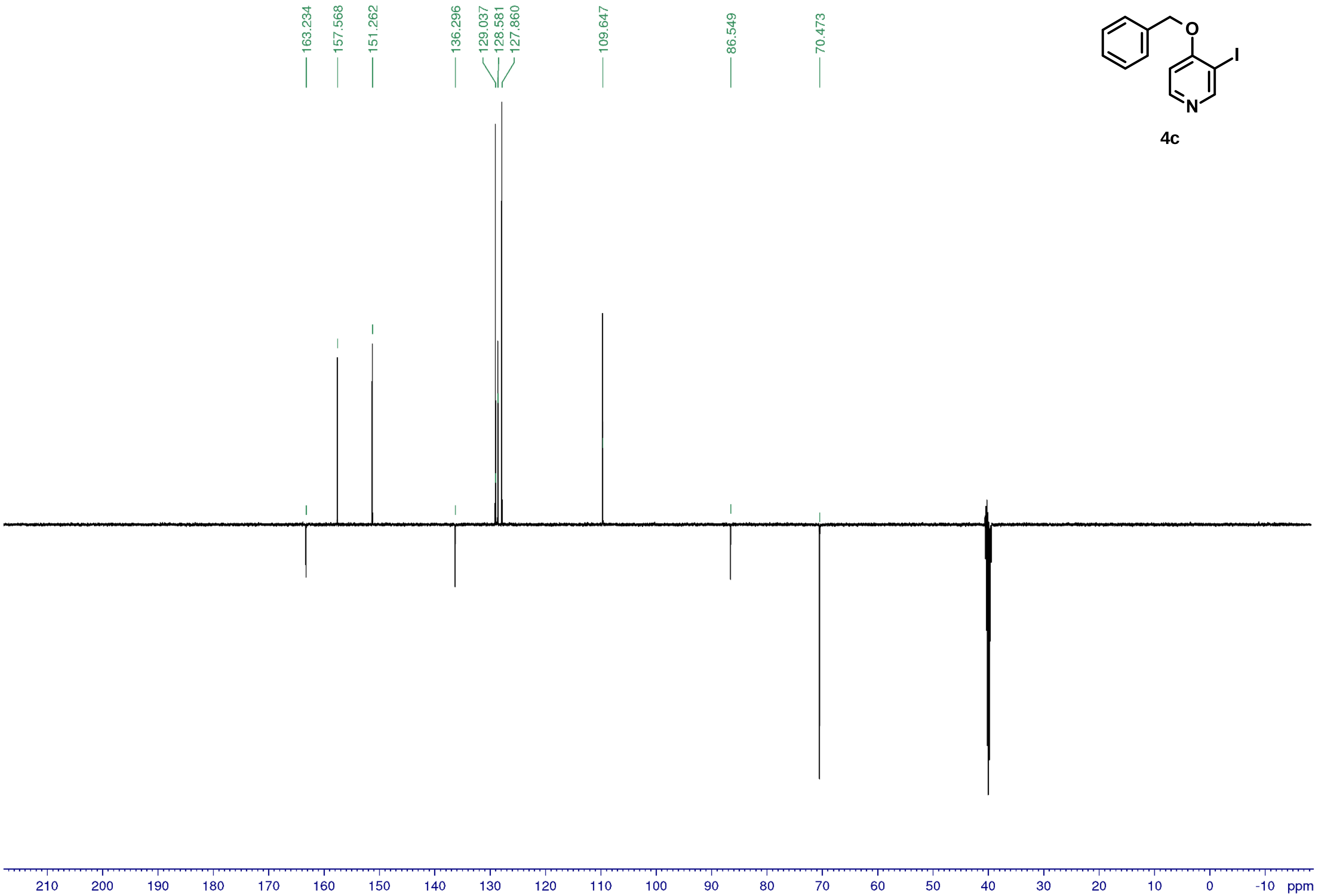
163.234
157.568
151.262
136.296
129.037
128.581
127.860
109.647
86.549
70.473
210
200
190
180
170
160
150
140
130
120
110
100
90
80
70
60
50
40
30
20
10
0
-10
ppm
O
I
N

4c

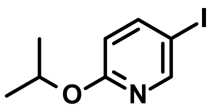

6b

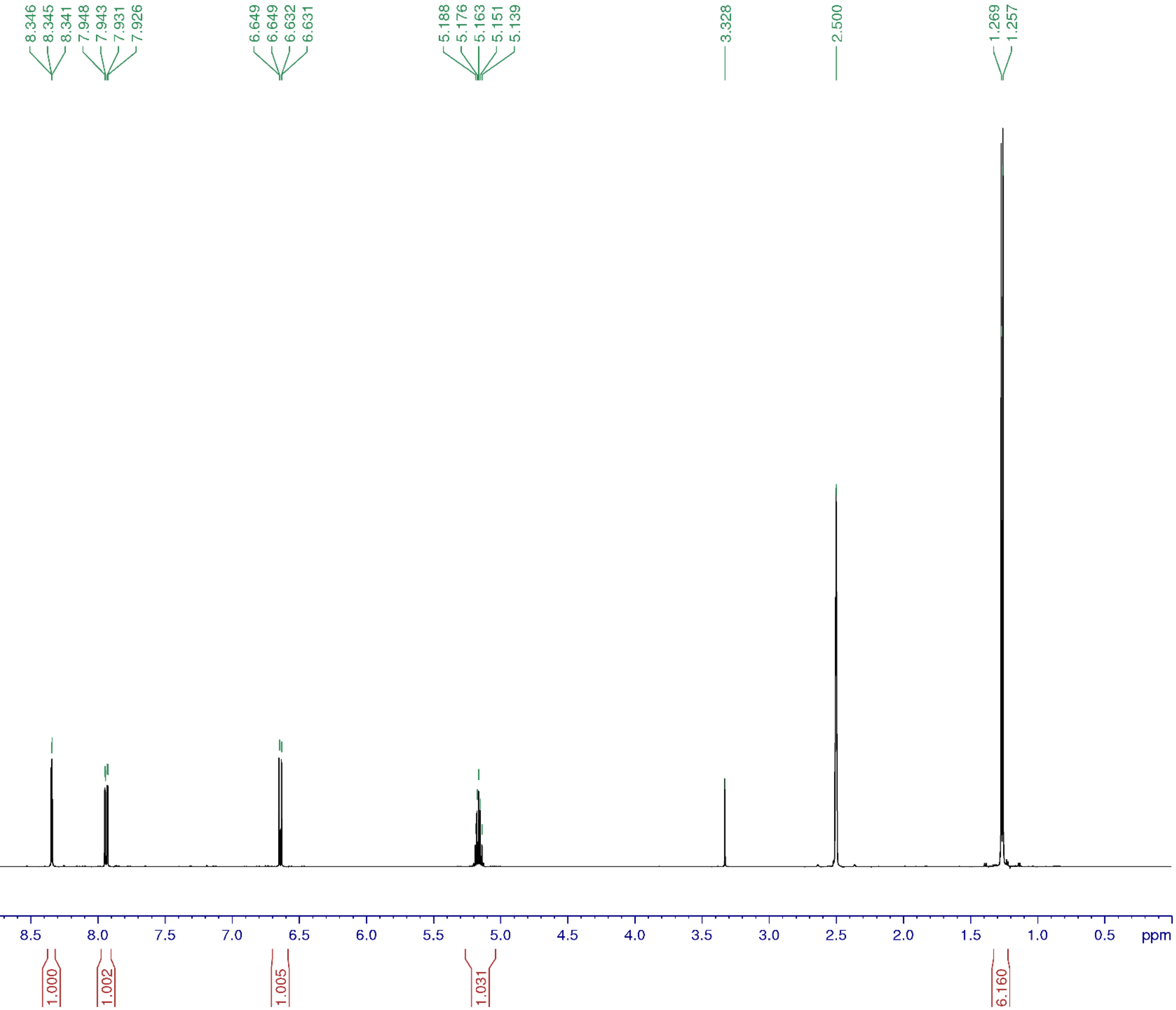

8.346
8.345
8.341
7.948
7.943
7.931
7.926
6.649
6.649
6.632
6.631
5.188
5.176
5.163
5.151
5.139
3.328
2.500
1.269
1.257
10.5
10.0
9.5
9.0
8.5
8.0
7.5
7.0
6.5
6.0
5.5
5.0
4.5
4.0
3.5
3.0
2.5
2.0
1.5
1.0
0.5
ppm
1.000
1.002
1.005
1.031
6.160

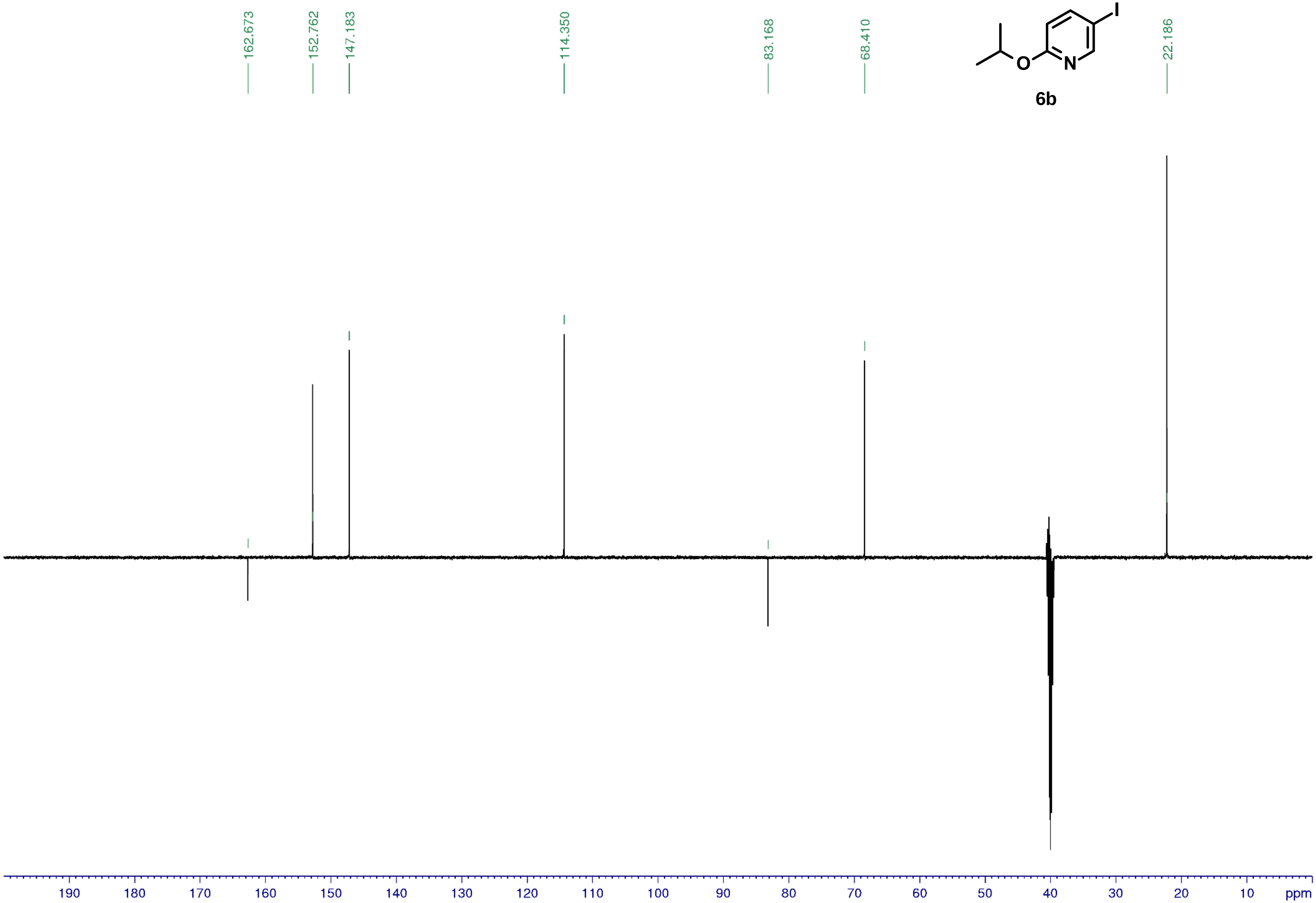
162.673
152.762
147.183
114.350
83.168
68.410
22.186
6b
190
180
170
160
150
140
130
120
110
100
90
80
70
60
50
40
30
20
10
ppm

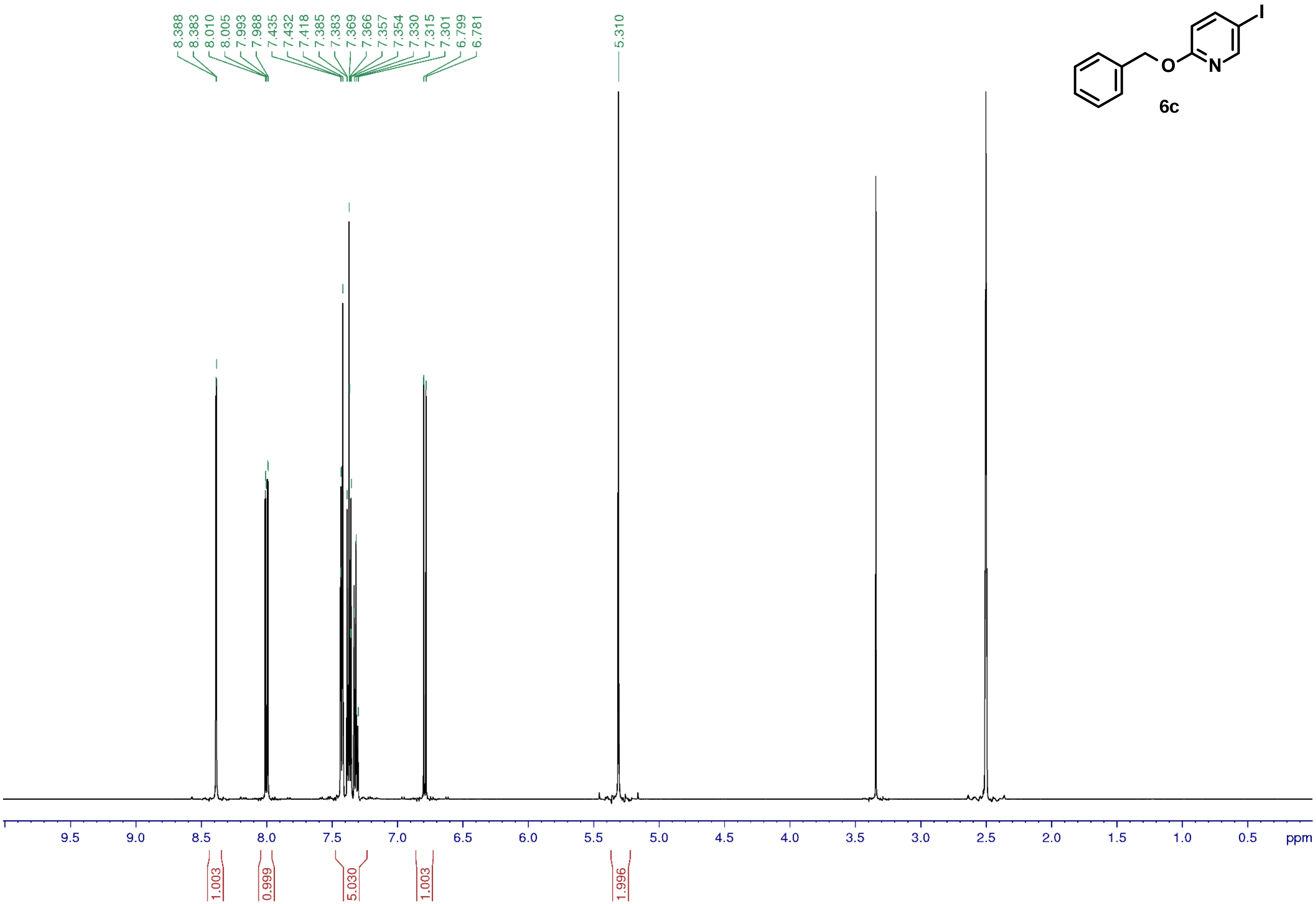
8.388
8.383
8.010
8.005
7.993
7.988
7.435
7.432
7.418
7.385
7.383
7.369
7.366
7.357
7.354
7.330
7.315
7.301
6.799
6.781
5.310
O
N
I
9.5 9.0 8.5 8.0 7.5 7.0 6.5 6.0 5.5 5.0 4.5 4.0 3.5 3.0 2.5 2.0 1.5 1.0 0.5 ppm
1.003
0.999
5.030
1.003
1.996

6c

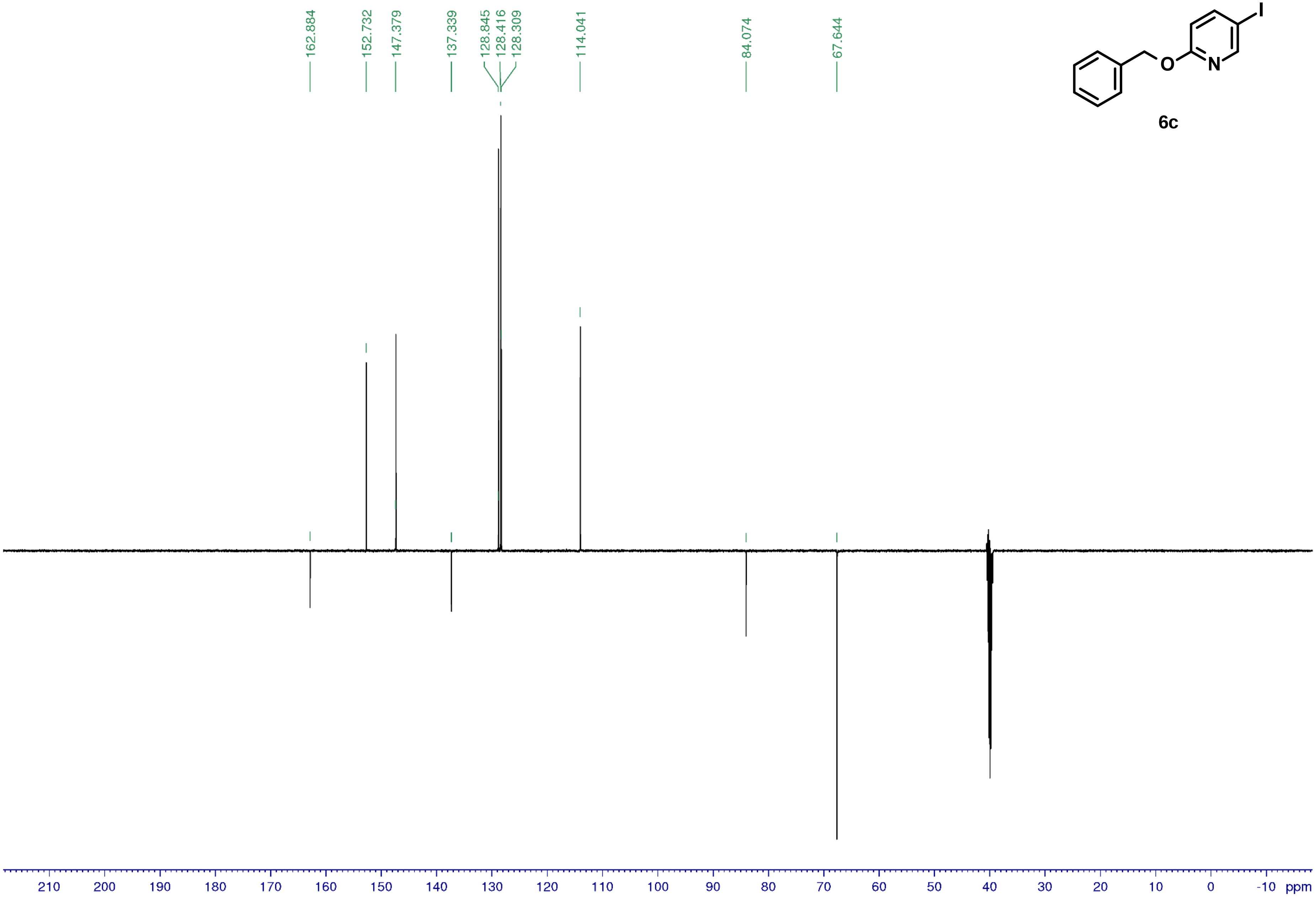

162.884
152.732
147.379
137.339
128.845
128.416
128.309
114.041
84.074
67.644
O
N
I
6c
210
200
190
180
170
160
150
140
130
120
110
100
90
80
70
60
50
40
30
20
10
0
-10
ppm

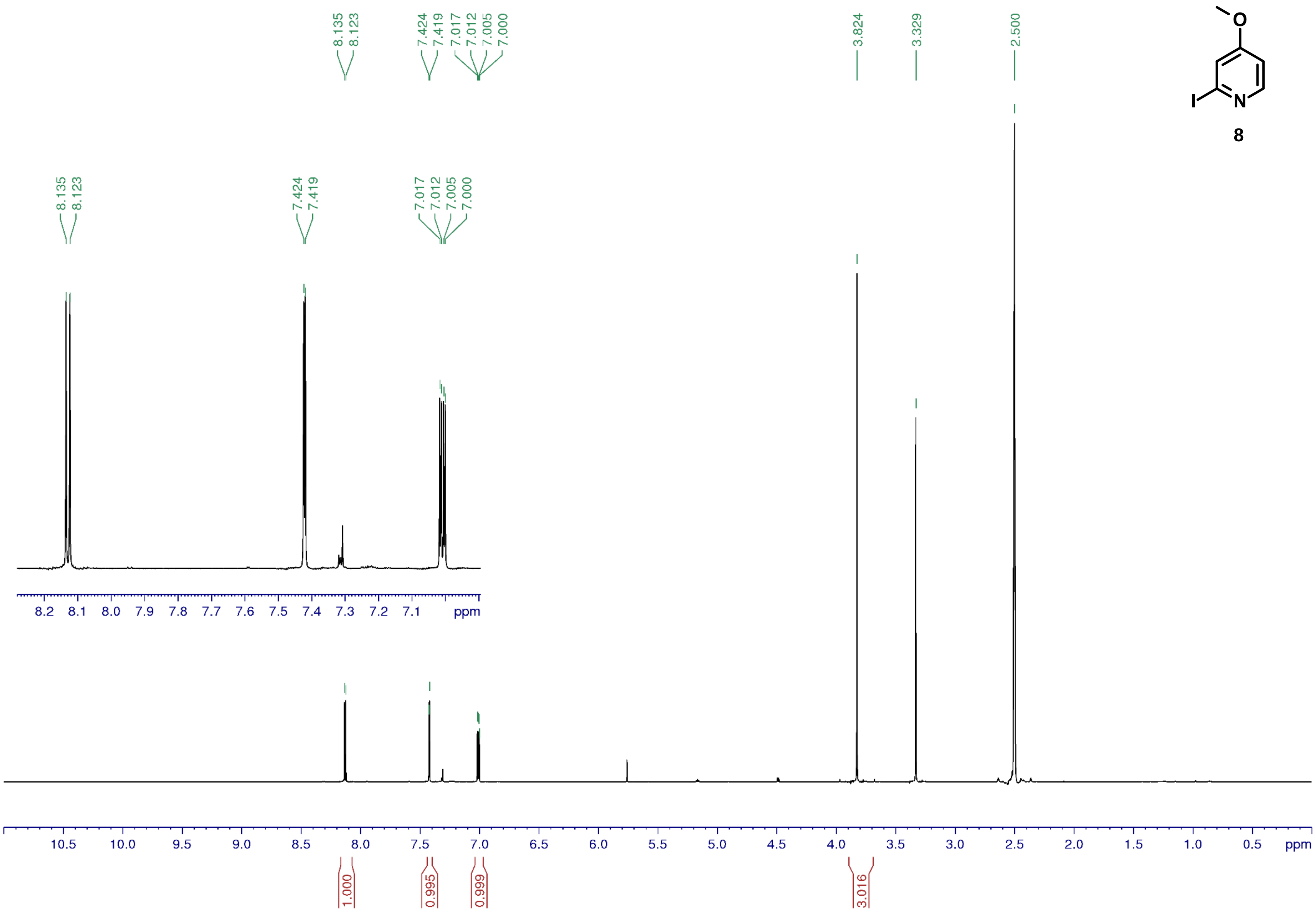
8.135
8.123
7.424
7.419
7.017
7.012
7.005
7.000
3.824
3.329
2.500
1.000
0.995
0.999
3.016
ppm
O
N
I

8

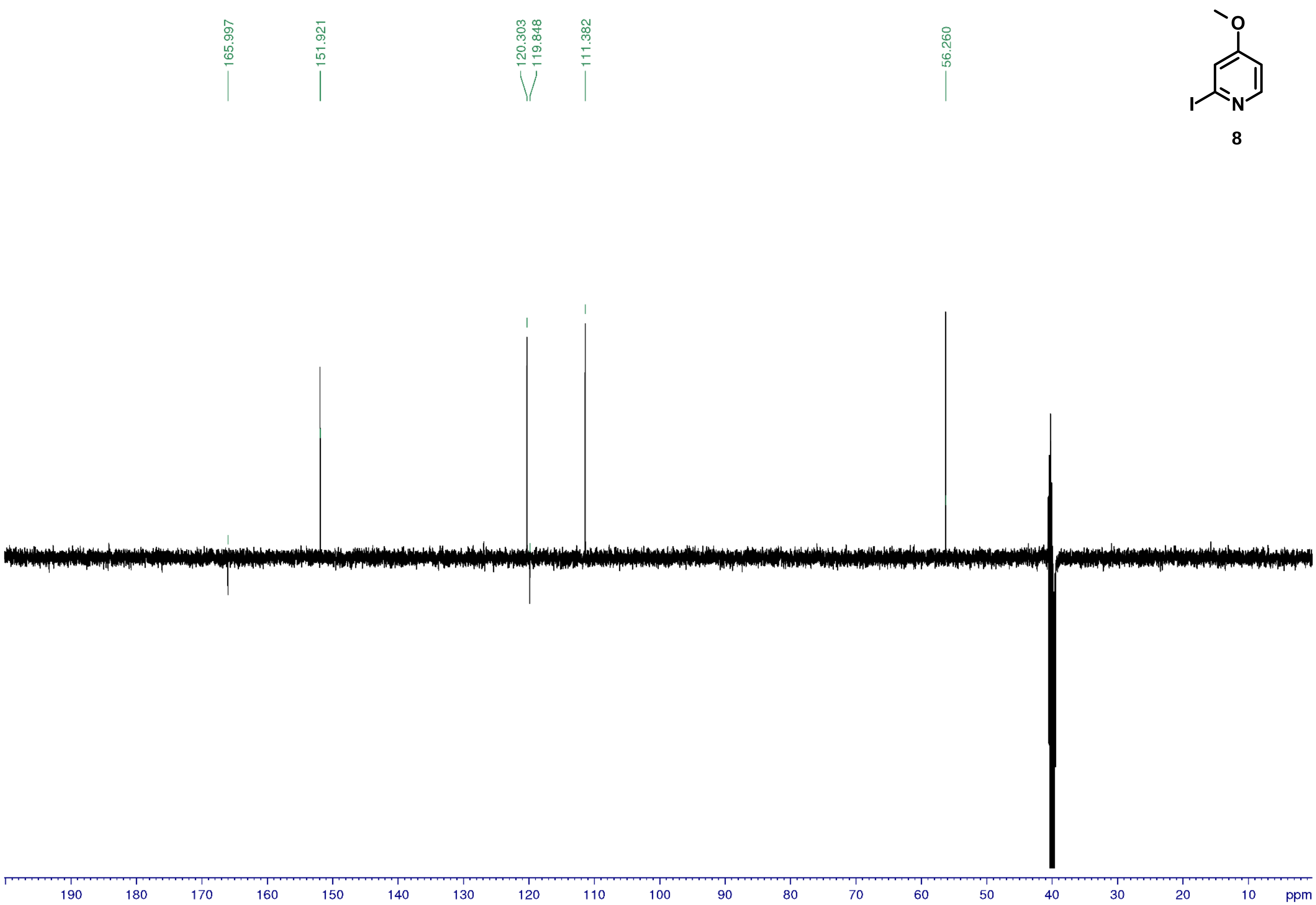

165.997
151.921
120.303
119.848
111.382
56.260
O
I
N
8
190
180
170
160
150
140
130
120
110
100
90
80
70
60
50
40
30
20
10
ppm

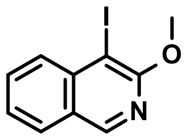

10

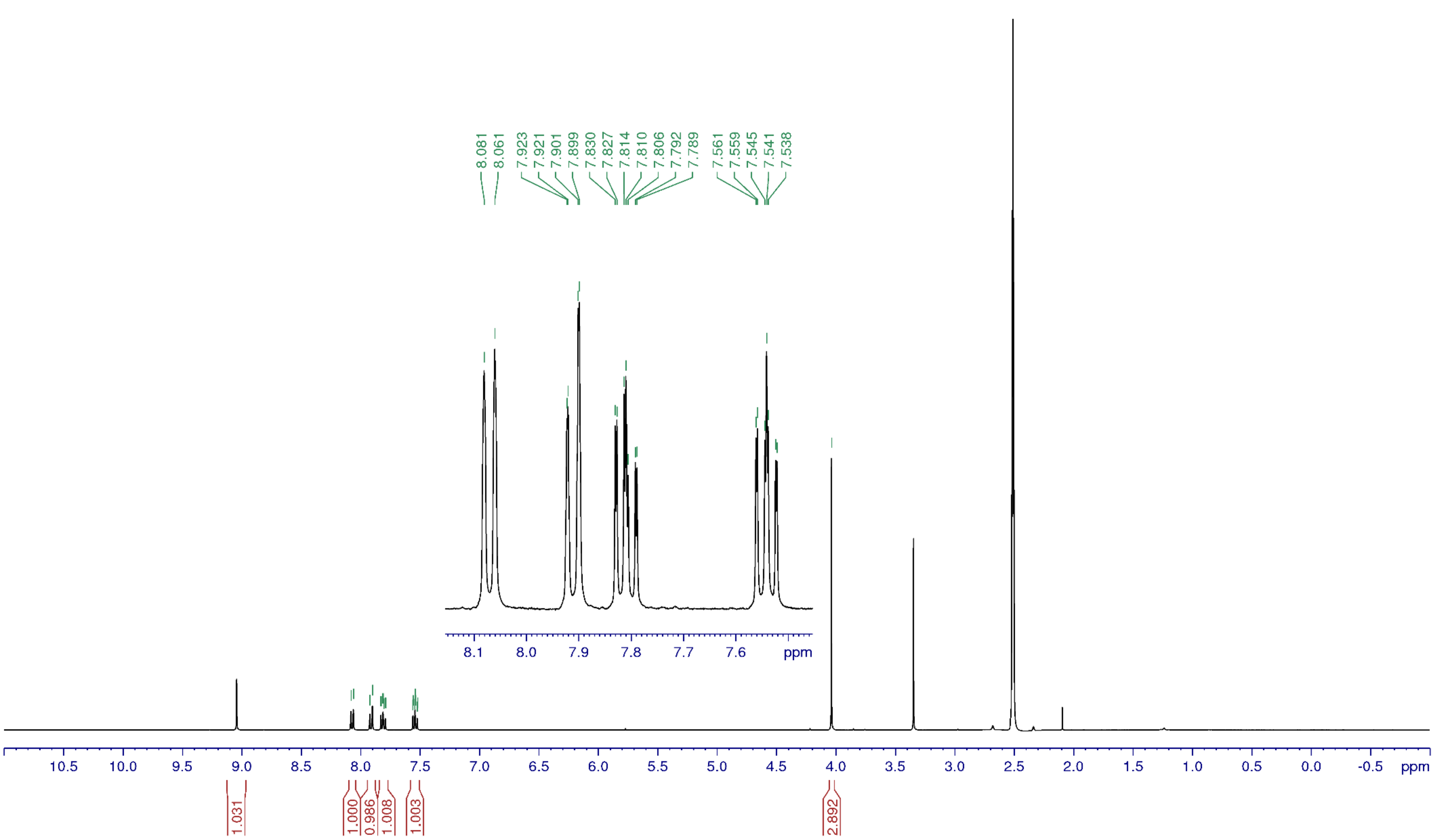

8.081
8.061
7.923
7.921
7.901
7.899
7.830
7.827
7.814
7.810
7.806
7.792
7.789
7.561
7.559
7.545
7.541
7.538
7.524
7.521
4.036
8.1 8.0 7.9 7.8 7.7 7.6 ppm
10.5 10.0 9.5 9.0 8.5 8.0 7.5 7.0 6.5 6.0 5.5 5.0 4.5 4.0 3.5 3.0 2.5 2.0 1.5 1.0 0.5 0.0 -0.5 ppm
1.031
1.000
0.986
1.008
1.003
2.892

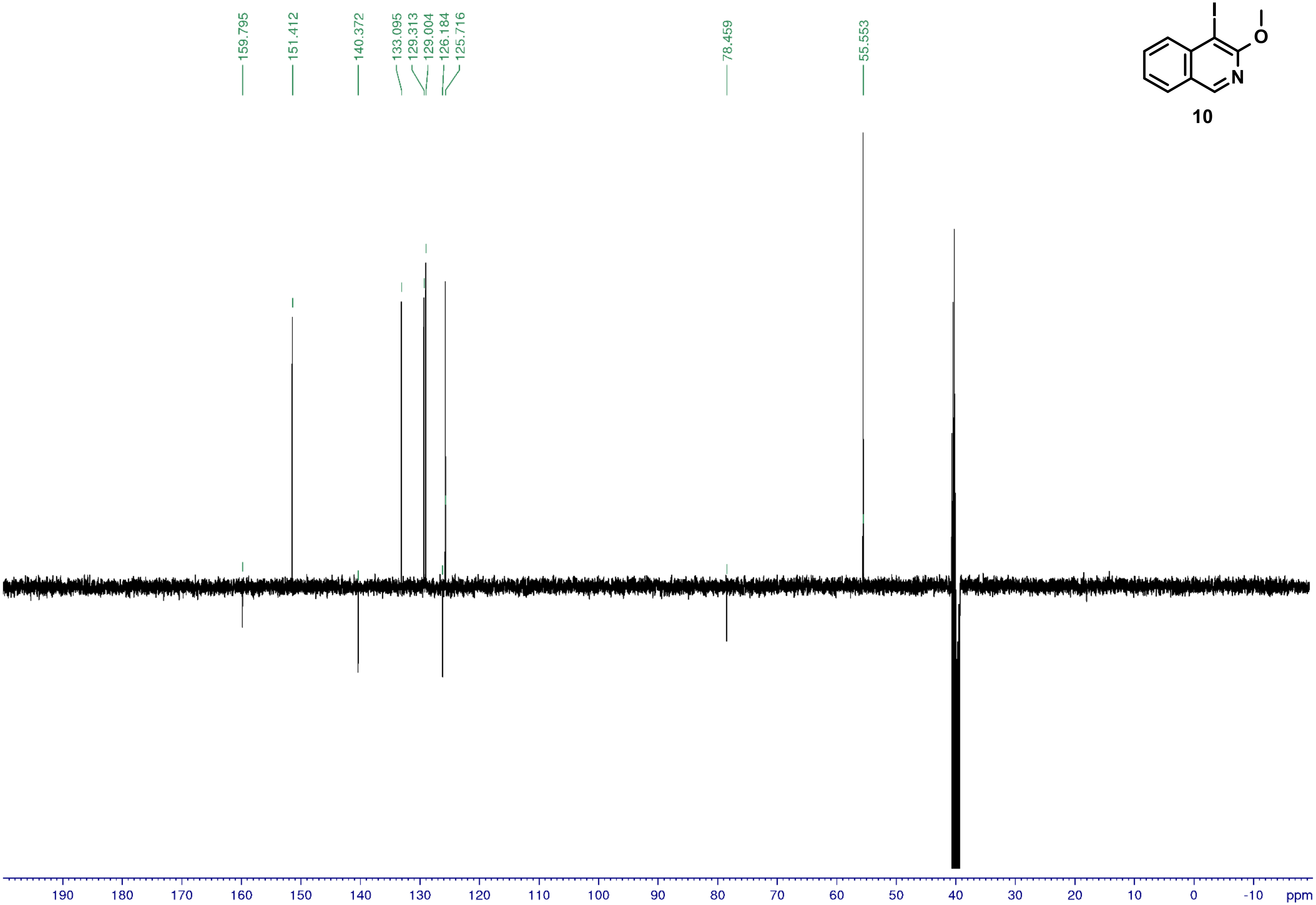
159.795
151.412
140.372
133.095
129.313
129.004
126.184
125.716
78.459
55.553
I
O
N
10
190
180
170
160
150
140
130
120
110
100
90
80
70
60
50
40
30
20
10
0
-10
ppm

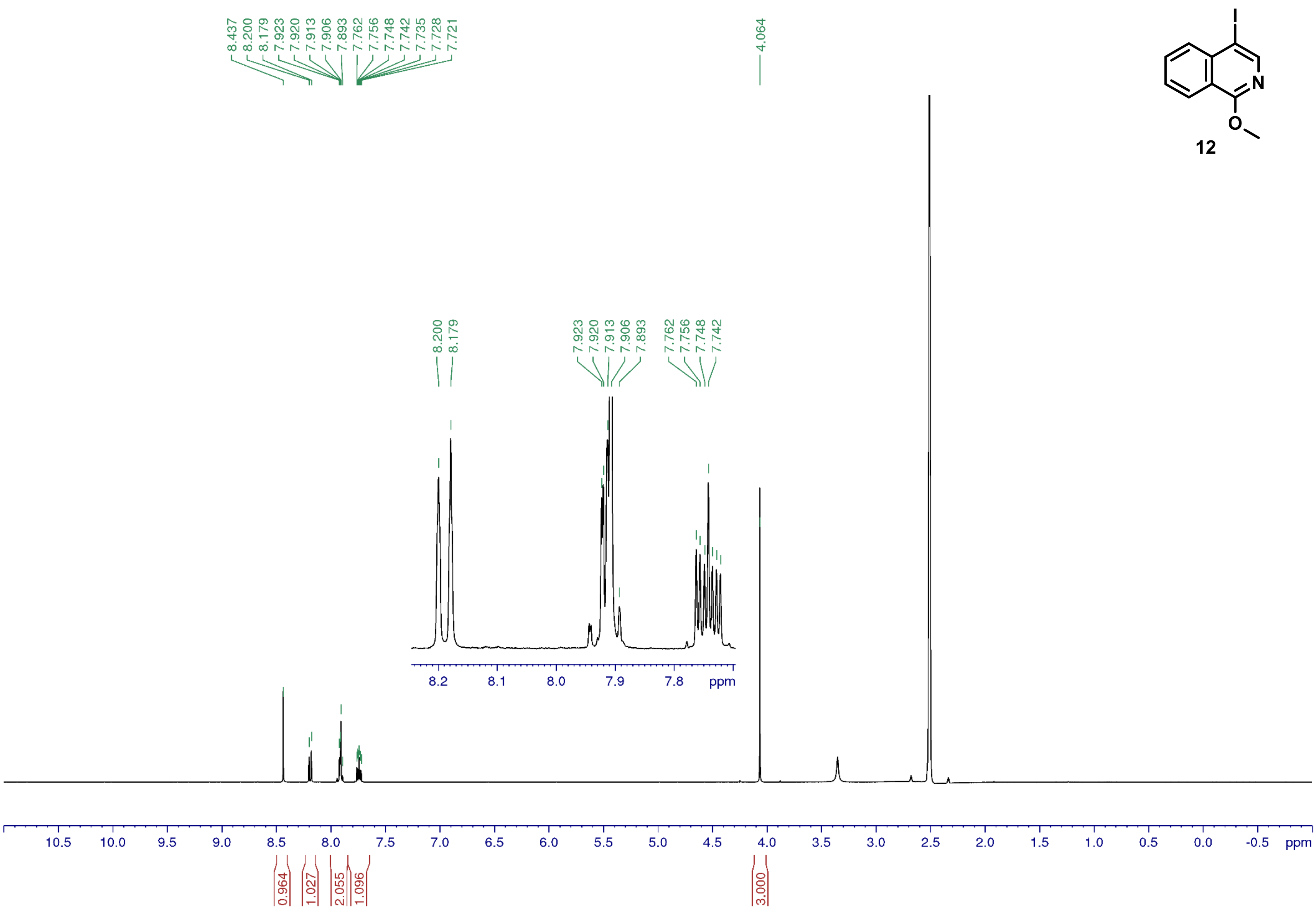

8.437
8.200
8.179
7.923
7.920
7.913
7.906
7.893
7.762
7.756
7.748
7.742
7.735
7.728
7.721
4.064
N
O
I
12
8.2
8.1
8.0
7.9
7.8
ppm
10.5
10.0
9.5
9.0
8.5
8.0
7.5
7.0
6.5
6.0
5.5
5.0
4.5
4.0
3.5
3.0
2.5
2.0
1.5
1.0
0.5
0.0
-0.5
0.964
1.027
2.055
1.096
3.000

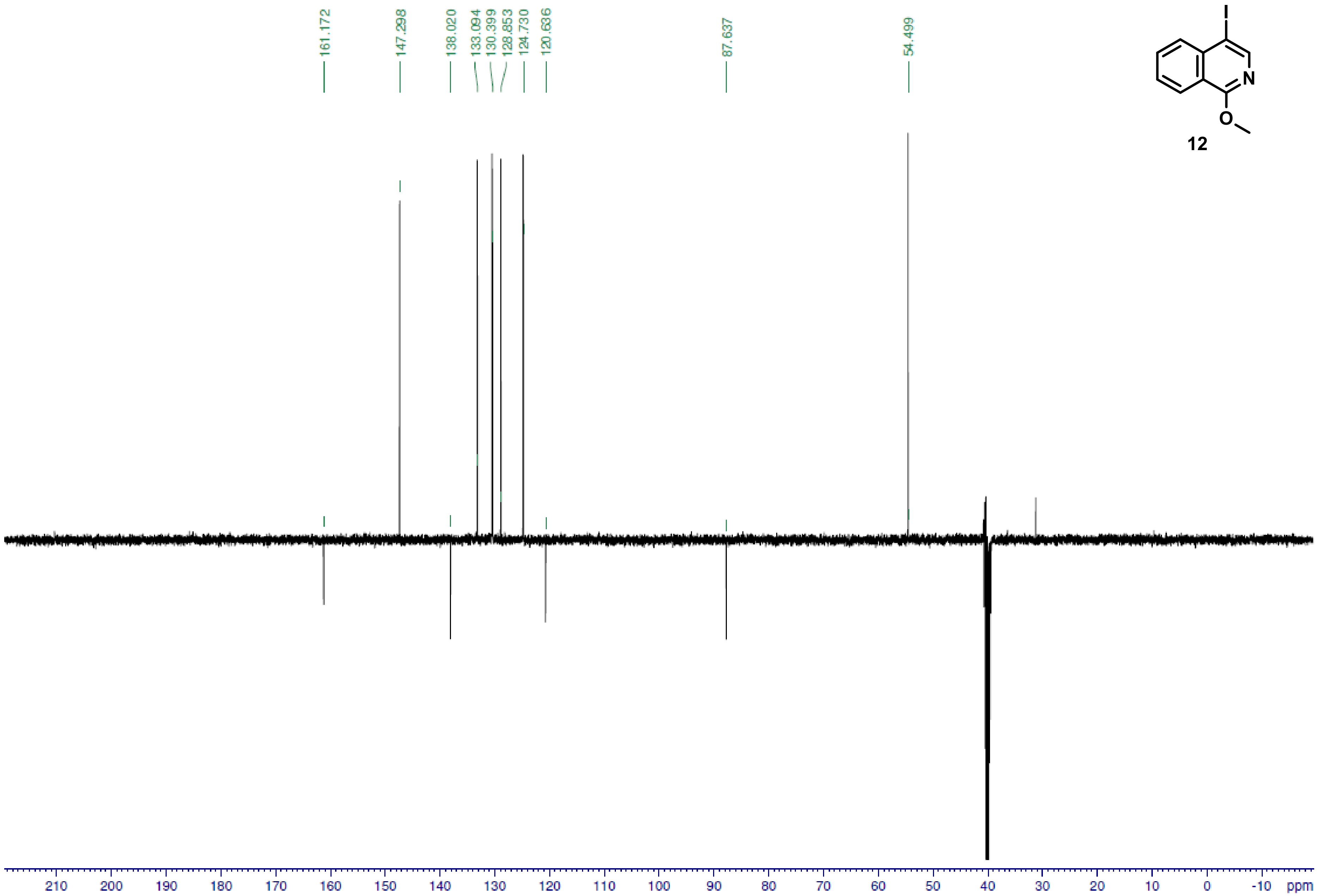
161.172
147.298
138.020
133.094
130.399
128.853
124.730
120.636
87.637
54.499
210 200 190 180 170 160 150 140 130 120 110 100 90 80 70 60 50 40 30 20 10 0 -10 ppm
I
N
O

12

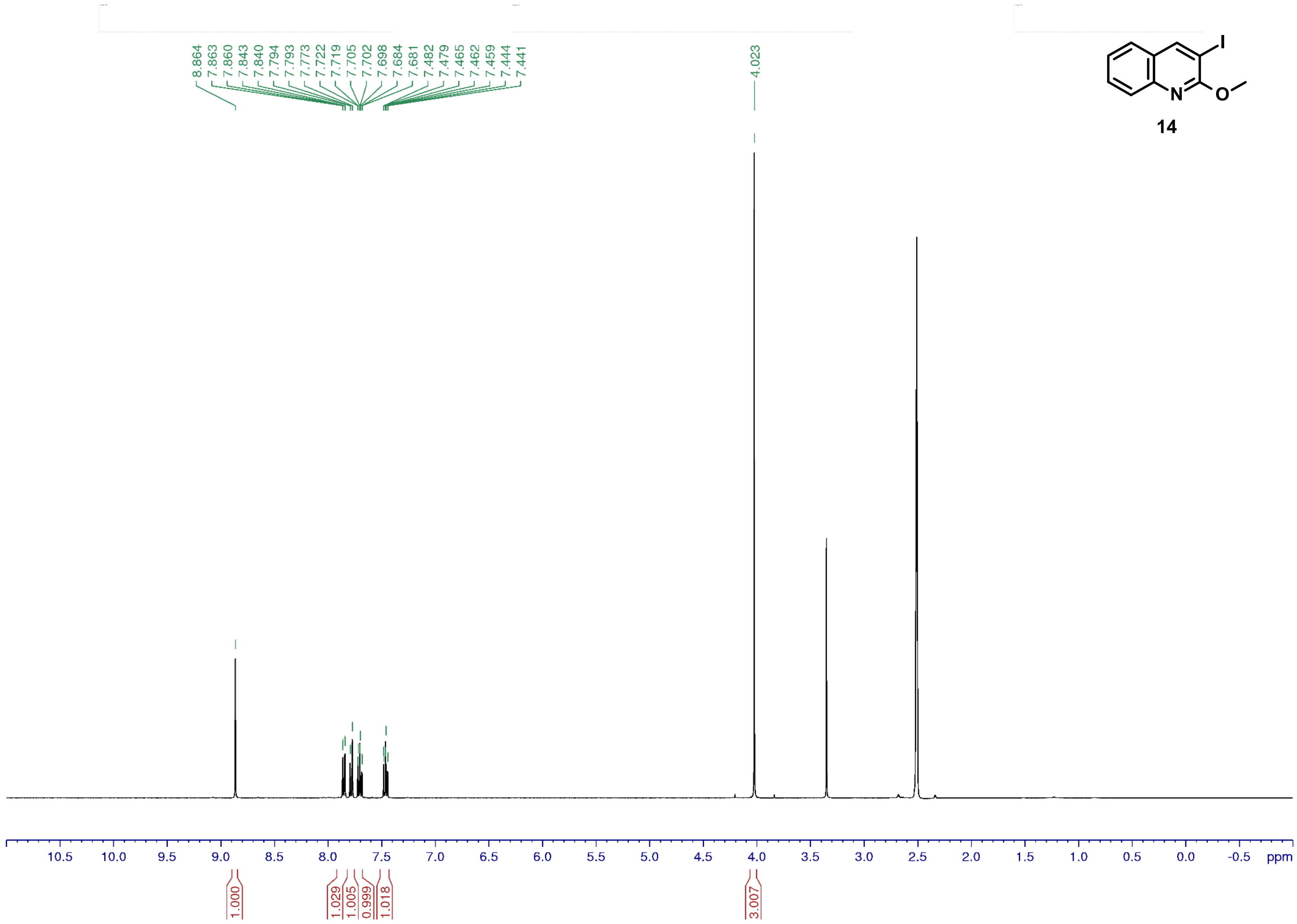

8.864
7.863
7.860
7.843
7.840
7.794
7.793
7.773
7.722
7.719
7.705
7.702
7.698
7.684
7.681
7.482
7.479
7.465
7.462
7.459
7.444
7.441
4.023
10.5 10.0 9.5 9.0 8.5 8.0 7.5 7.0 6.5 6.0 5.5 5.0 4.5 4.0 3.5 3.0 2.5 2.0 1.5 1.0 0.5 0.0 -0.5 ppm
1.000
1.029
1.005
0.999
1.018
3.007
I
N
O

14

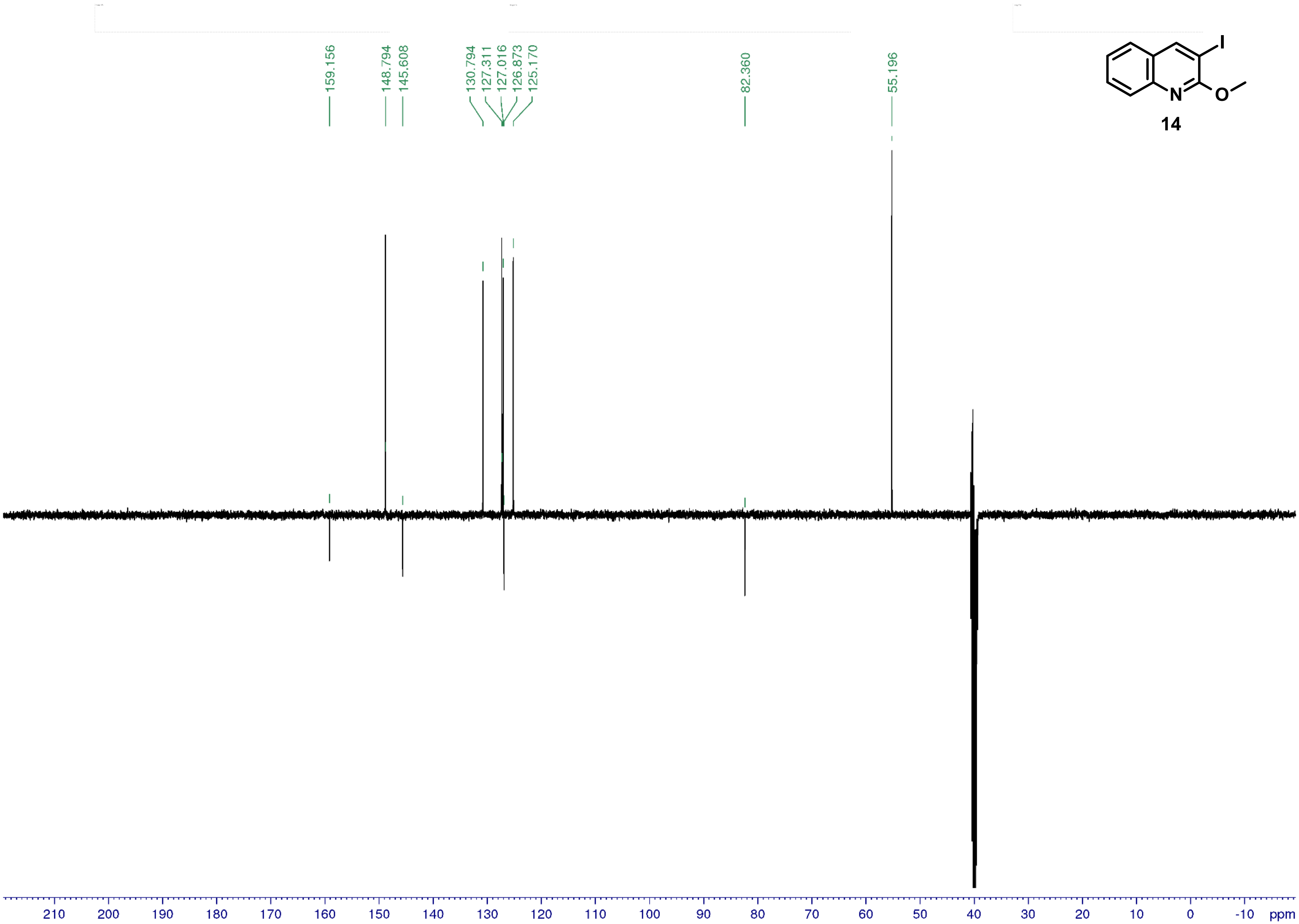

159.156
148.794
145.608
130.794
127.311
127.016
126.873
125.170
82.360
55.196
I
N
O
210
200
190
180
170
160
150
140
130
120
110
100
90
80
70
60
50
40
30
20
10
0
-10
ppm

14

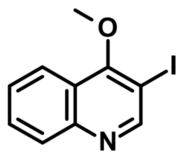
O
I
N

16

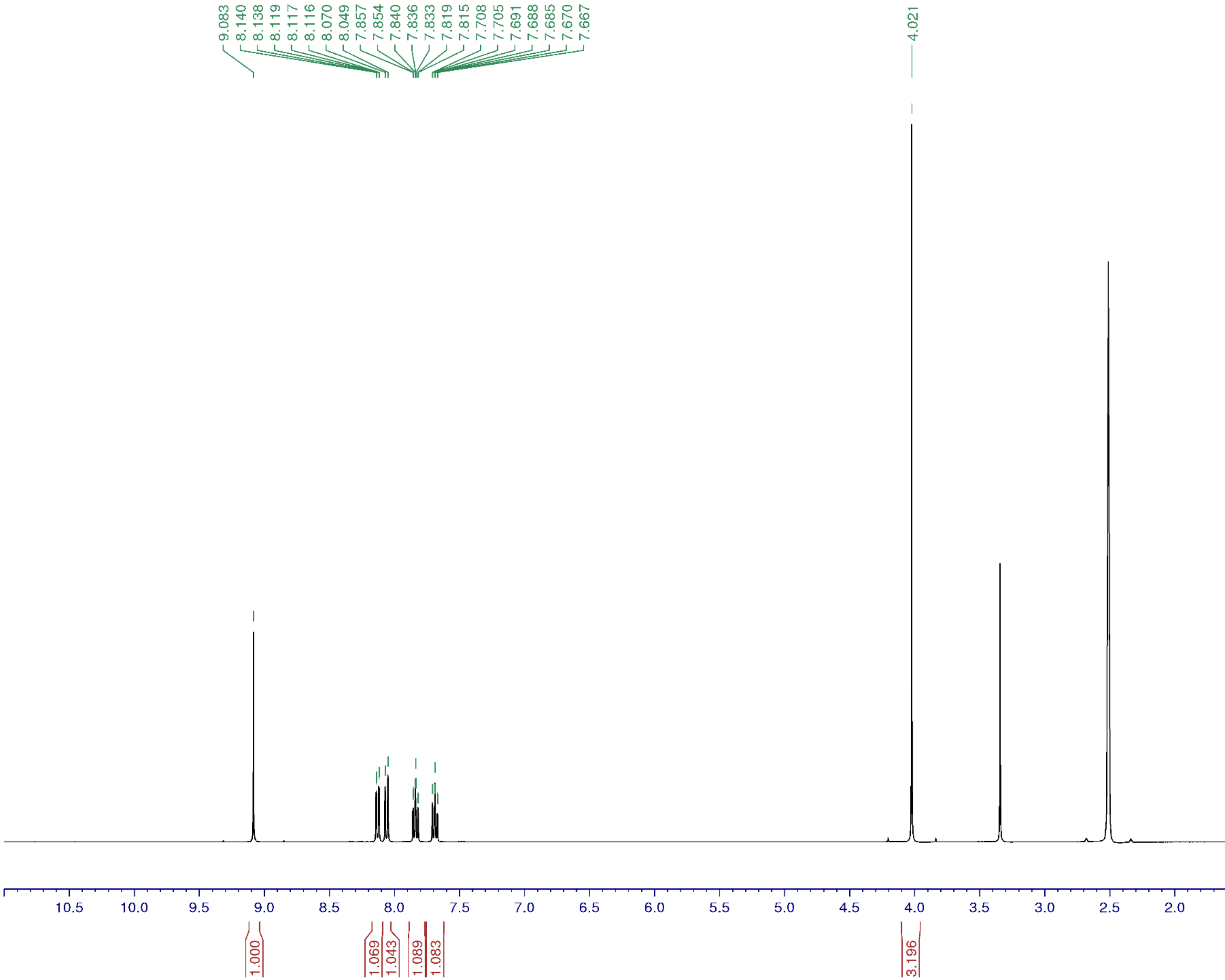
9.083
8.140
8.138
8.119
8.117
8.116
8.070
8.049
7.857
7.854
7.840
7.836
7.833
7.819
7.815
7.708
7.705
7.691
7.688
7.685
7.670
7.667
4.021
10.5
10.0
9.5
9.0
8.5
8.0
7.5
7.0
6.5
6.0
5.5
5.0
4.5
4.0
3.5
3.0
2.5
2.0
1.5
1.0
0.5
ppm
1.000
1.069
1.043
1.089
1.083
3.196

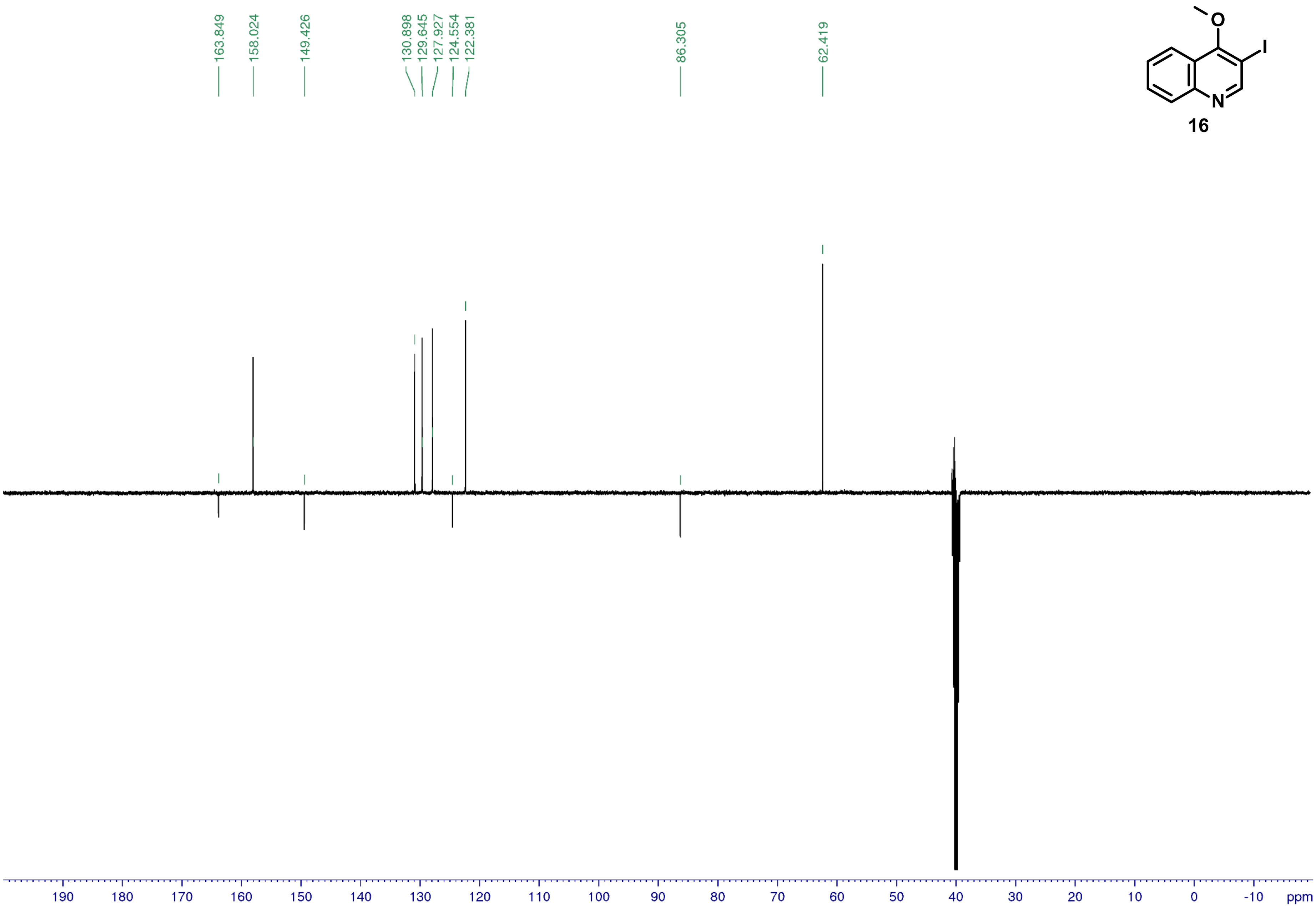
163.849
158.024
149.426
130.898
129.645
127.927
124.554
122.381
86.305
62.419
190
180
170
160
150
140
130
120
110
100
90
80
70
60
50
40
30
20
10
0
-10
ppm
O
I
N

16

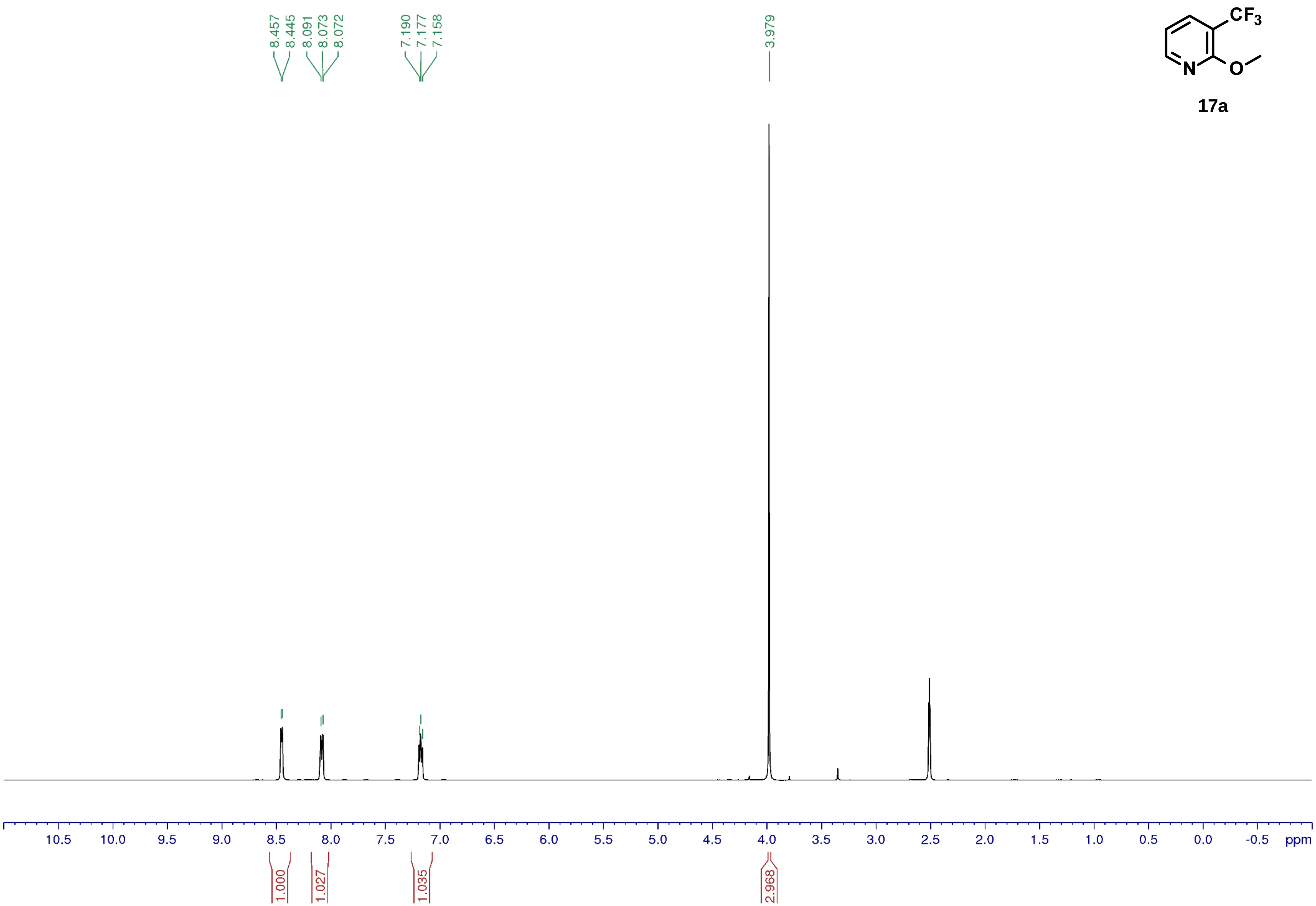
8.457
8.445
8.091
8.073
8.072
7.190
7.177
7.158
3.979
10.5
10.0
9.5
9.0
8.5
8.0
7.5
7.0
6.5
6.0
5.5
5.0
4.5
4.0
3.5
3.0
2.5
2.0
1.5
1.0
0.5
0.0
-0.5
ppm
1.000
1.027
1.035
2.968
CF3
N
O

17a

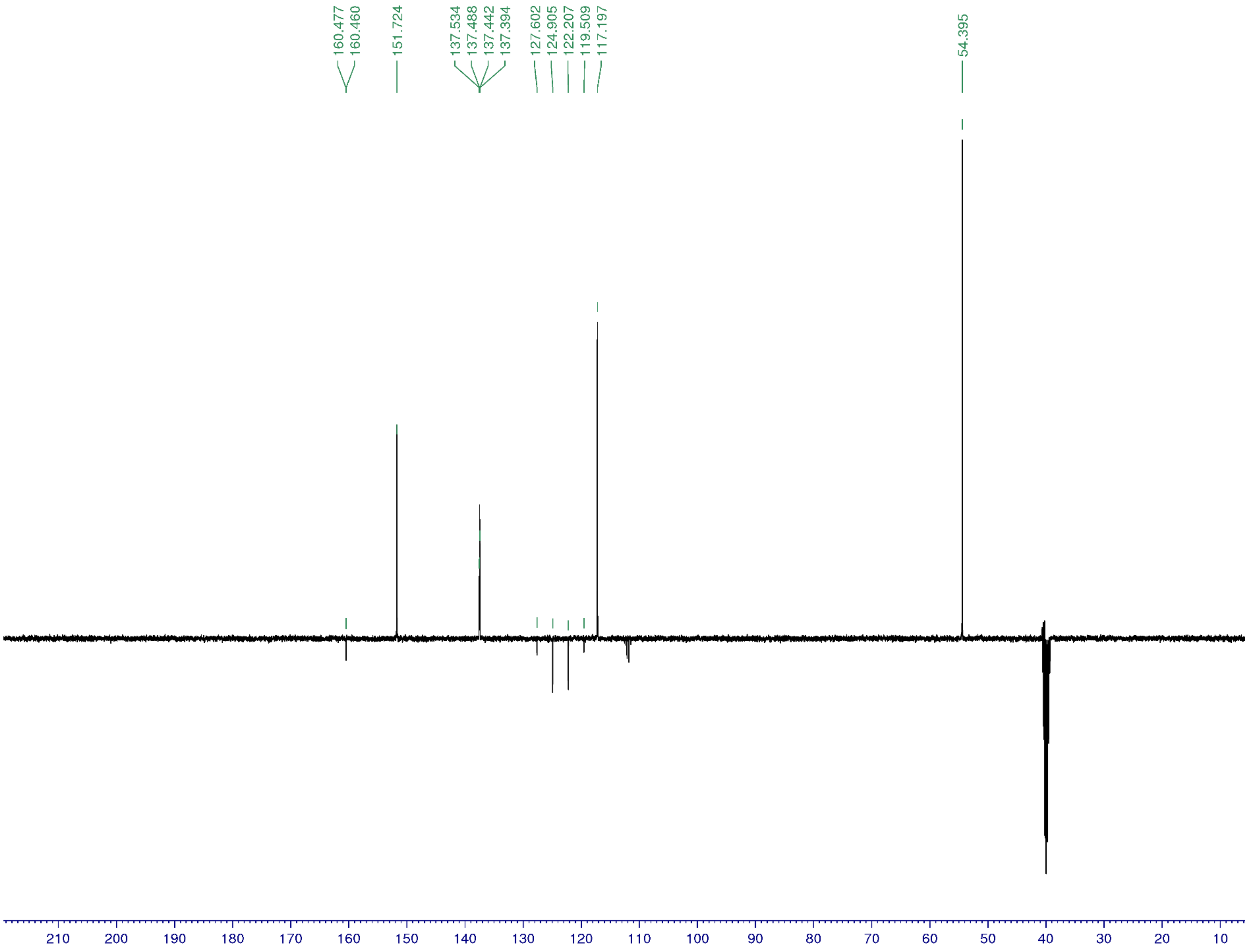
160.477
160.460
151.724
137.534
137.488
137.442
137.394
127.602
124.905
122.207
119.509
117.197
54.395
210 200 190 180 170 160 150 140 130 120 110 100 90 80 70 60 50 40 30 20 10 0 -10 ppm

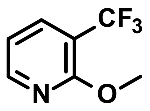
CF3
N
O

17a

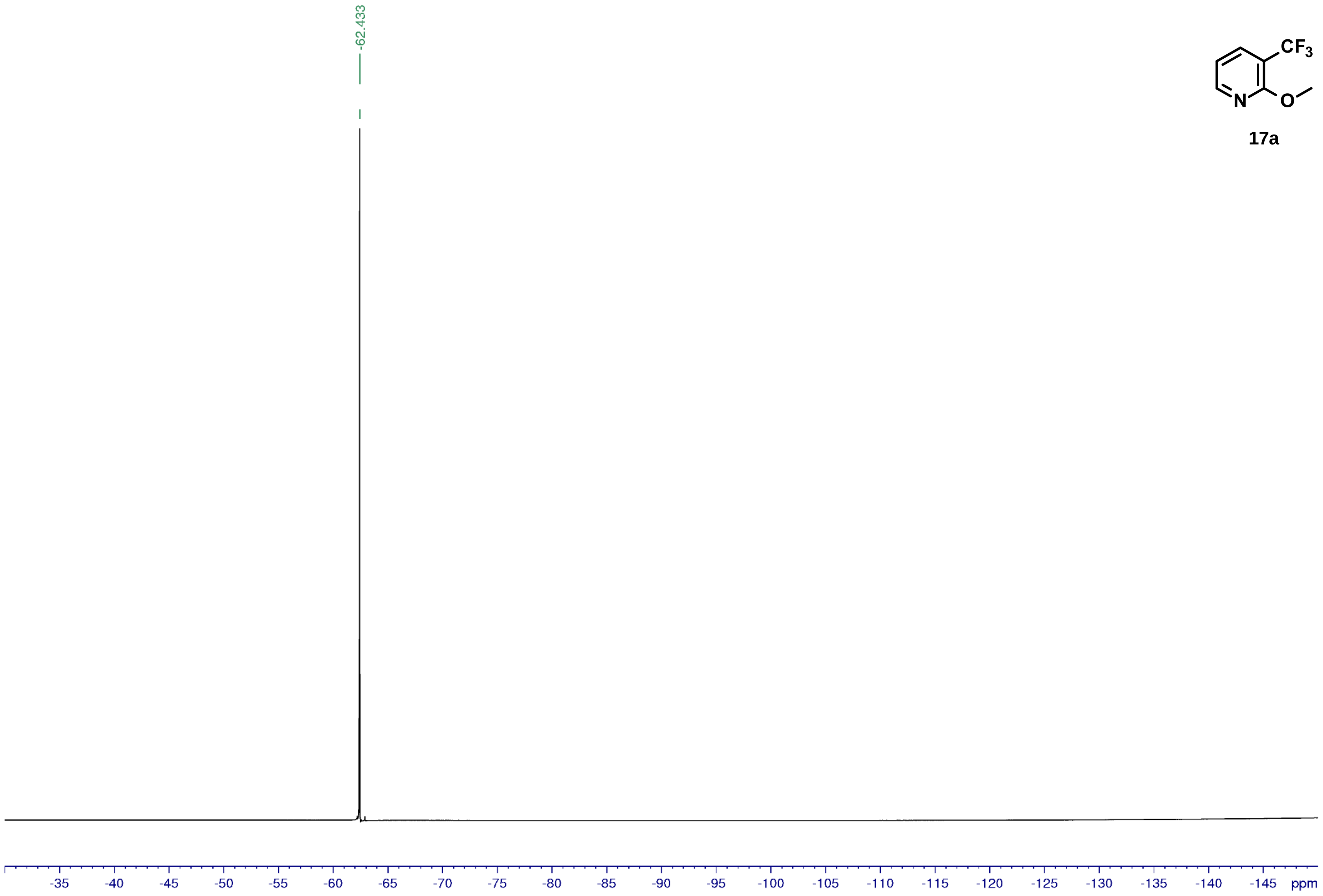

17a

-62.433
-35
-40
-45
-50
-55
-60
-65
-70
-75
-80
-85
-90
-95
-100
-105
-110
-115
-120
-125
-130
-135
-140
-145
ppm

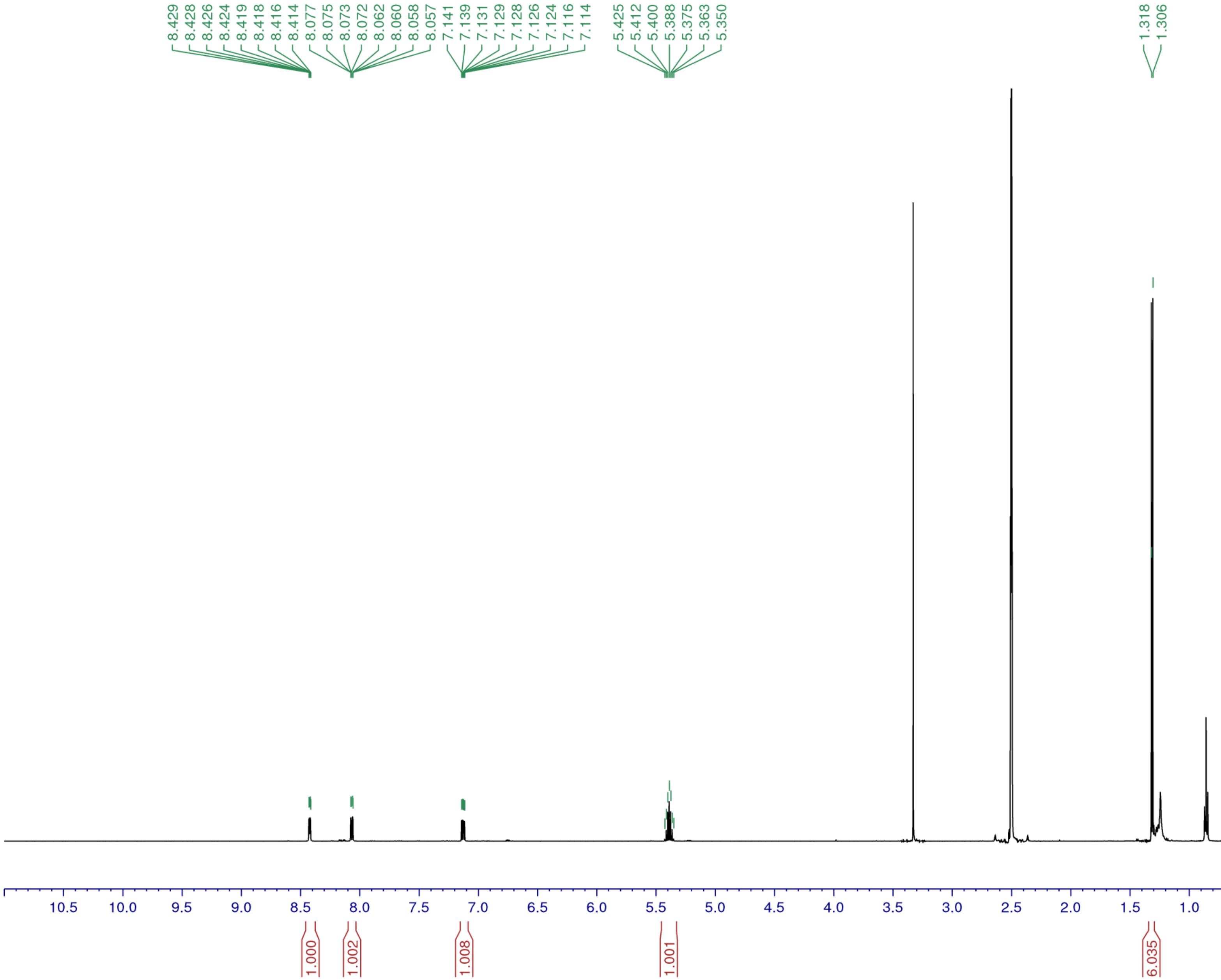
8.429
8.428
8.426
8.424
8.419
8.418
8.416
8.414
8.077
8.075
8.073
8.072
8.062
8.060
8.058
8.057
7.141
7.139
7.131
7.129
7.128
7.126
7.124
7.116
7.114
5.425
5.412
5.400
5.388
5.375
5.363
5.350
1.318
1.306
10.5
10.0
9.5
9.0
8.5
8.0
7.5
7.0
6.5
6.0
5.5
5.0
4.5
4.0
3.5
3.0
2.5
2.0
1.5
1.0
0.5
0.0
-0.5
ppm
1.000
1.002
1.008
1.001
6.035

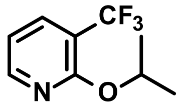
CF3
N
O

17b

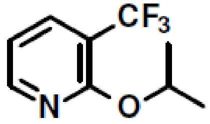
CF3
N
O

17b

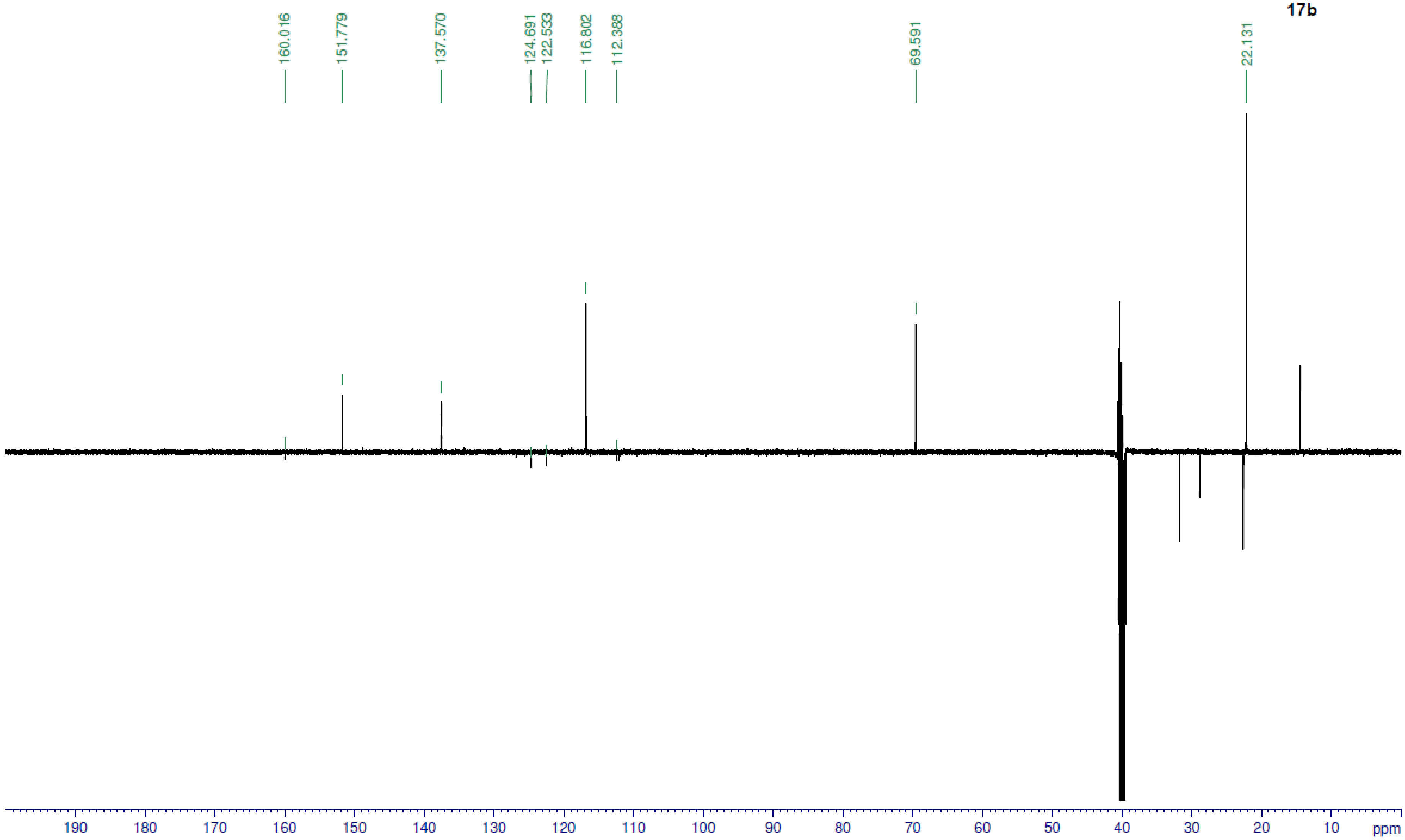
160.016
151.779
137.570
124.691
122.533
116.802
112.388
69.591
22.131
190
180
170
160
150
140
130
120
110
100
90
80
70
60
50
40
30
20
10
ppm

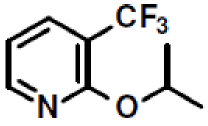

17b

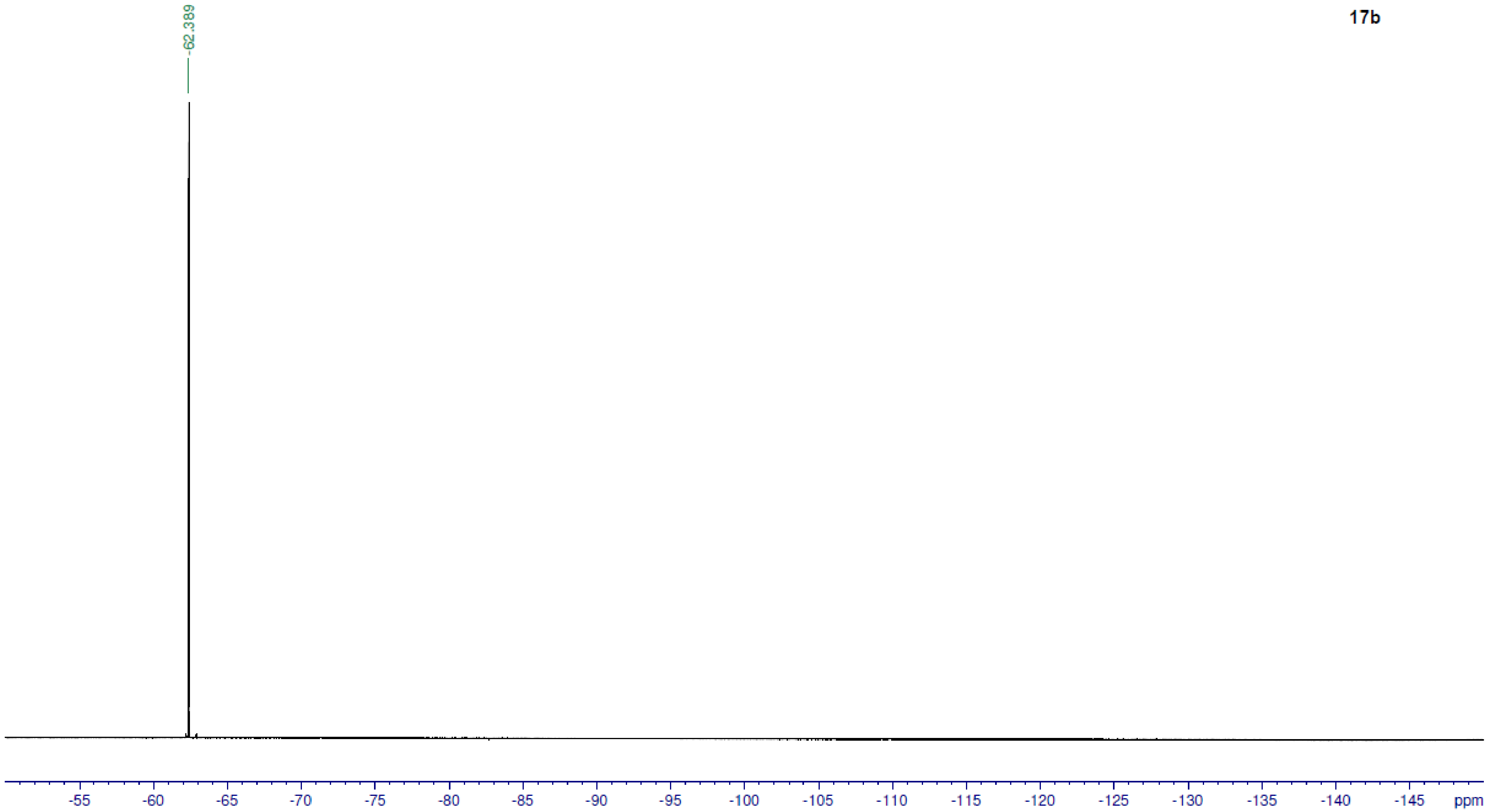

-62.389
-55
-60
-65
-70
-75
-80
-85
-90
-95
-100
-105
-110
-115
-120
-125
-130
-135
-140
-145
ppm

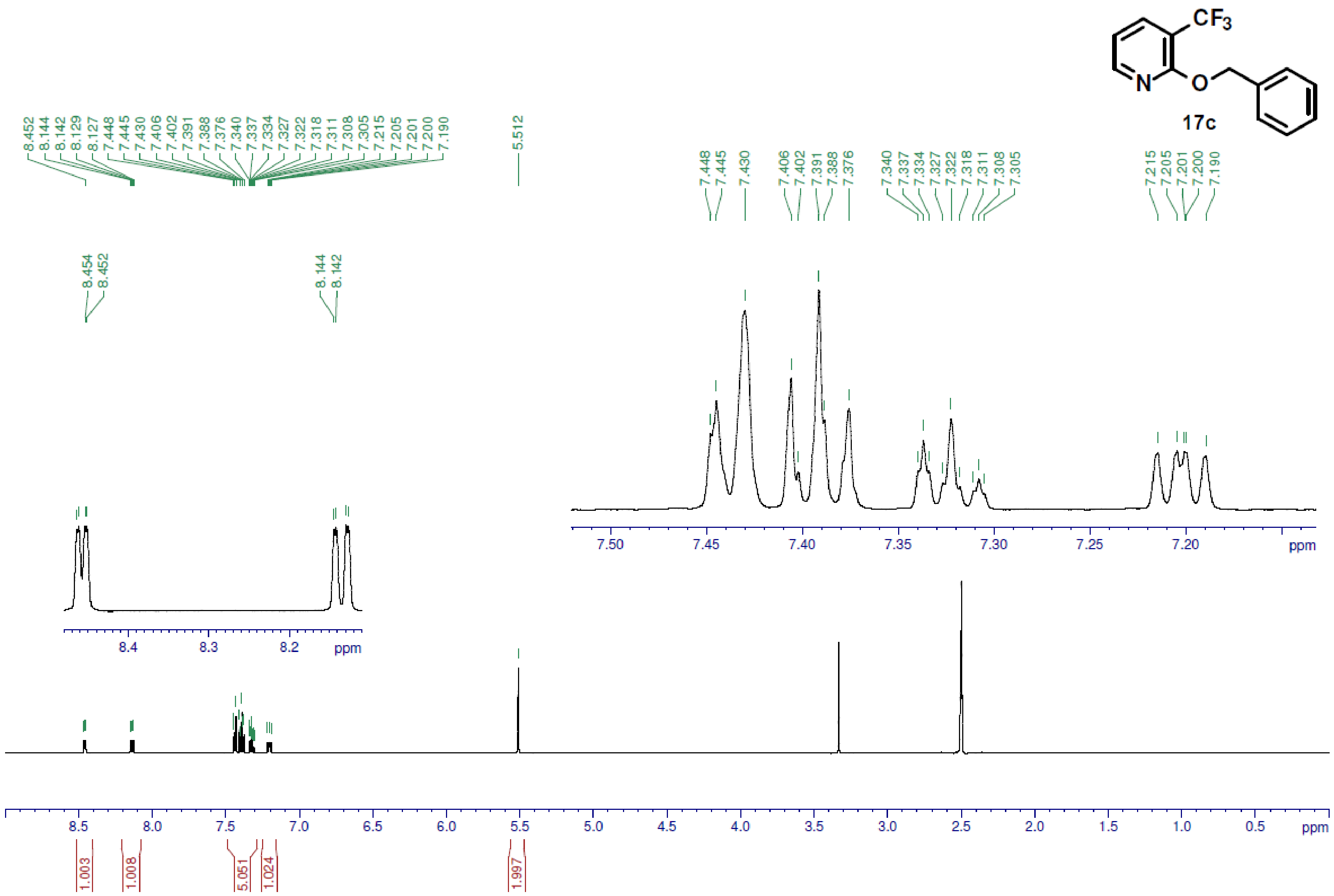

CF3
N
O
17c
8.452 8.144 8.142 8.129 8.127 7.448 7.445 7.430 7.406 7.402 7.391 7.388 7.376 7.340 7.337 7.334 7.327 7.322 7.318 7.311 7.308 7.305 7.215 7.205 7.201 7.200 7.190
5.512
7.448 7.445 7.430 7.406 7.402 7.391 7.388 7.376 7.340 7.337 7.334 7.327 7.322 7.318 7.311 7.308 7.305 7.215 7.205 7.201 7.200 7.190
8.454 8.452
8.144 8.142
7.50 7.45 7.40 7.35 7.30 7.25 7.20 ppm
8.4 8.3 8.2 ppm
8.5 8.0 7.5 7.0 6.5 6.0 5.5 5.0 4.5 4.0 3.5 3.0 2.5 2.0 1.5 1.0 0.5 ppm
1.003
1.008
5.051
1.024
1.997

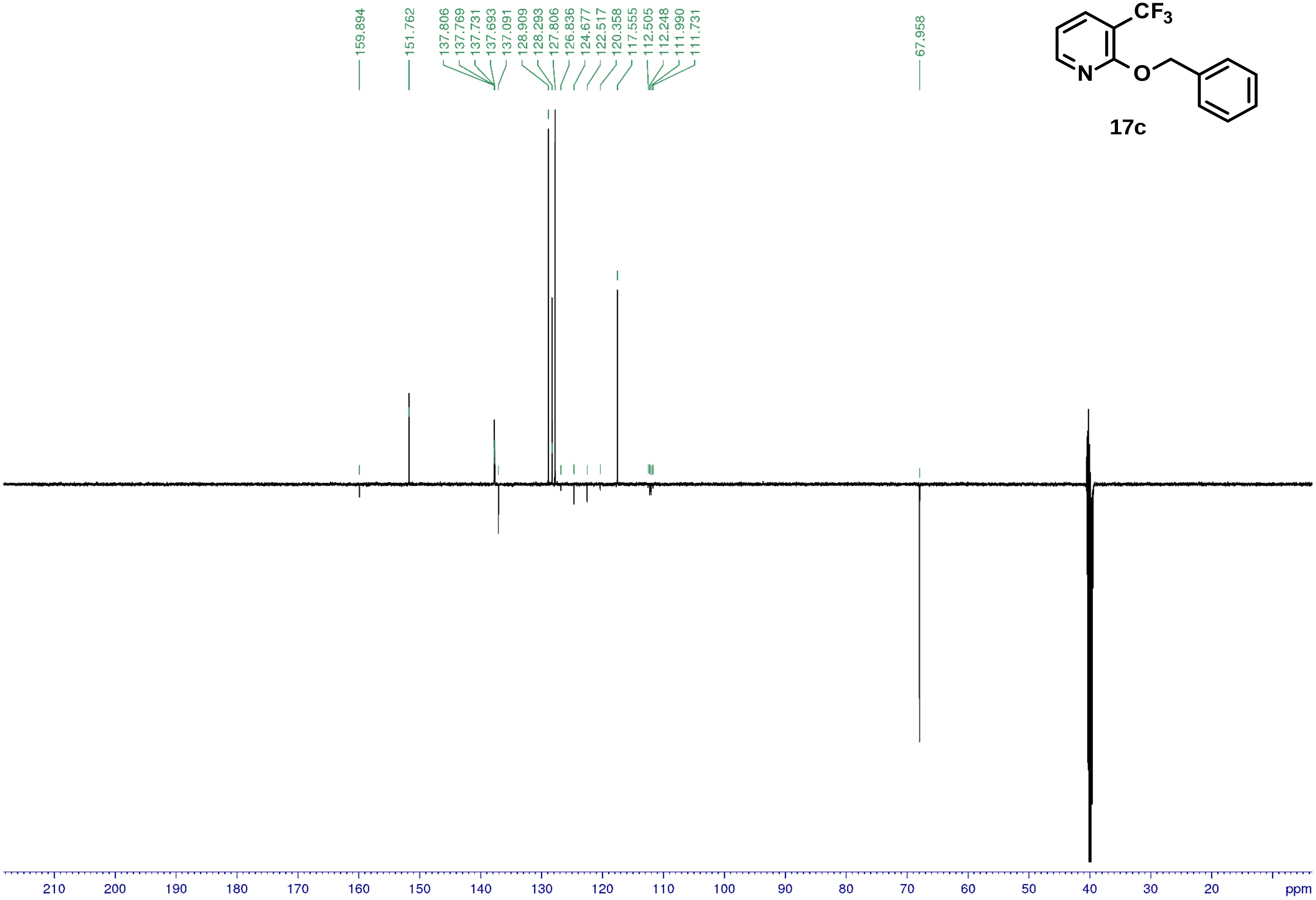
159.894
151.762
137.806
137.769
137.731
137.693
137.091
128.909
128.293
127.806
126.836
124.677
122.517
120.358
117.555
112.505
112.248
111.990
111.731
67.958
CF3
N
O
17c
210
200
190
180
170
160
150
140
130
120
110
100
90
80
70
60
50
40
30
20
ppm

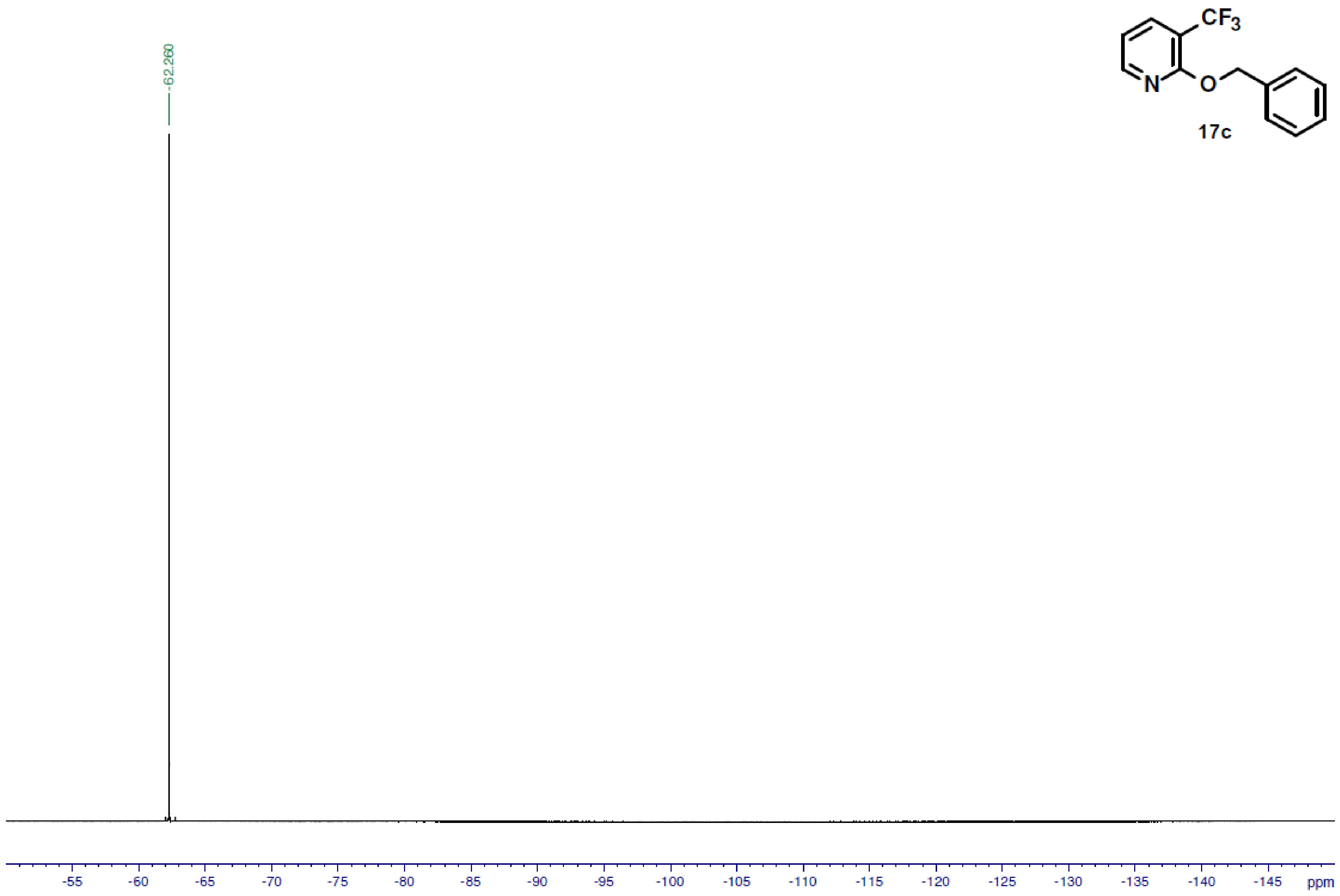
-62.260
CF3
N
O
17c
-55
-60
-65
-70
-75
-80
-85
-90
-95
-100
-105
-110
-115
-120
-125
-130
-135
-140
-145
ppm

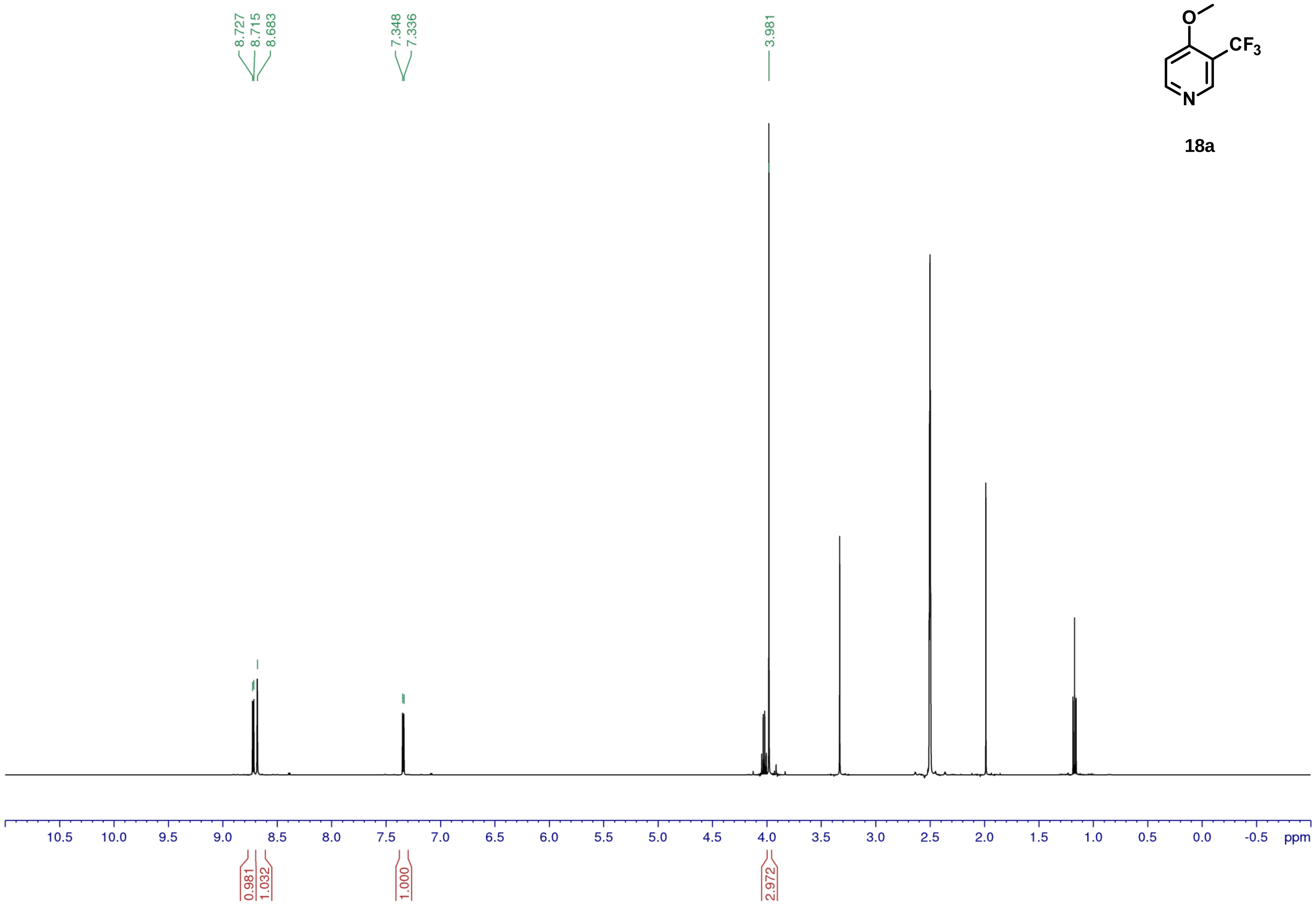

8.727
8.715
8.683
7.348
7.336
3.981
O
CF3
N
18a
10.5
10.0
9.5
9.0
8.5
8.0
7.5
7.0
6.5
6.0
5.5
5.0
4.5
4.0
3.5
3.0
2.5
2.0
1.5
1.0
0.5
0.0
-0.5
ppm
0.981
1.032
1.000
2.972

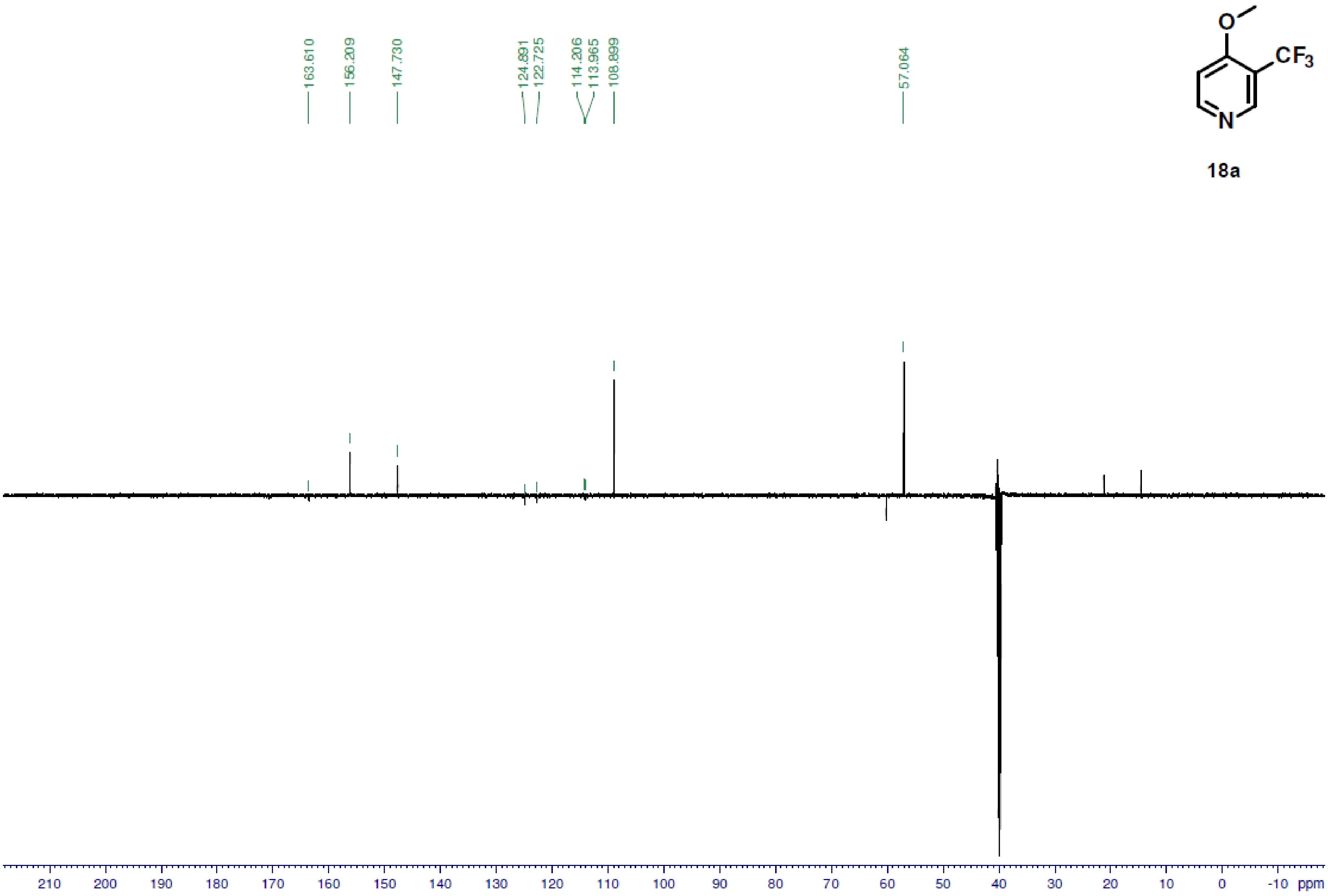
163.610
156.209
147.730
124.891
122.725
114.206
113.965
108.899
57.064
210 200 190 180 170 160 150 140 130 120 110 100 90 80 70 60 50 40 30 20 10 0 -10 ppm
O
CF3
N

18a

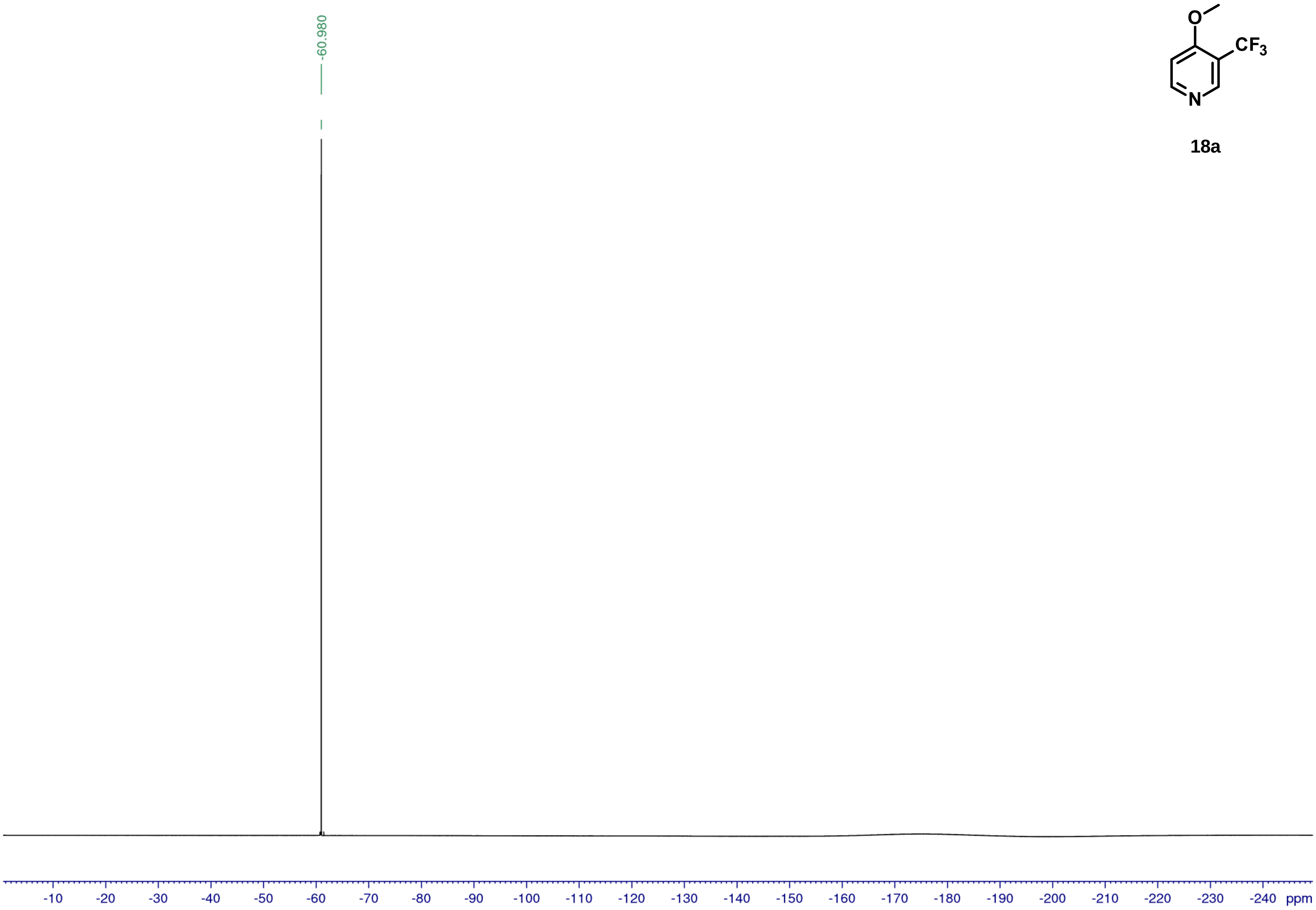
-60.980
-10
-20
-30
-40
-50
-60
-70
-80
-90
-100
-110
-120
-130
-140
-150
-160
-170
-180
-190
-200
-210
-220
-230
-240
ppm
O
CF3
N

18a

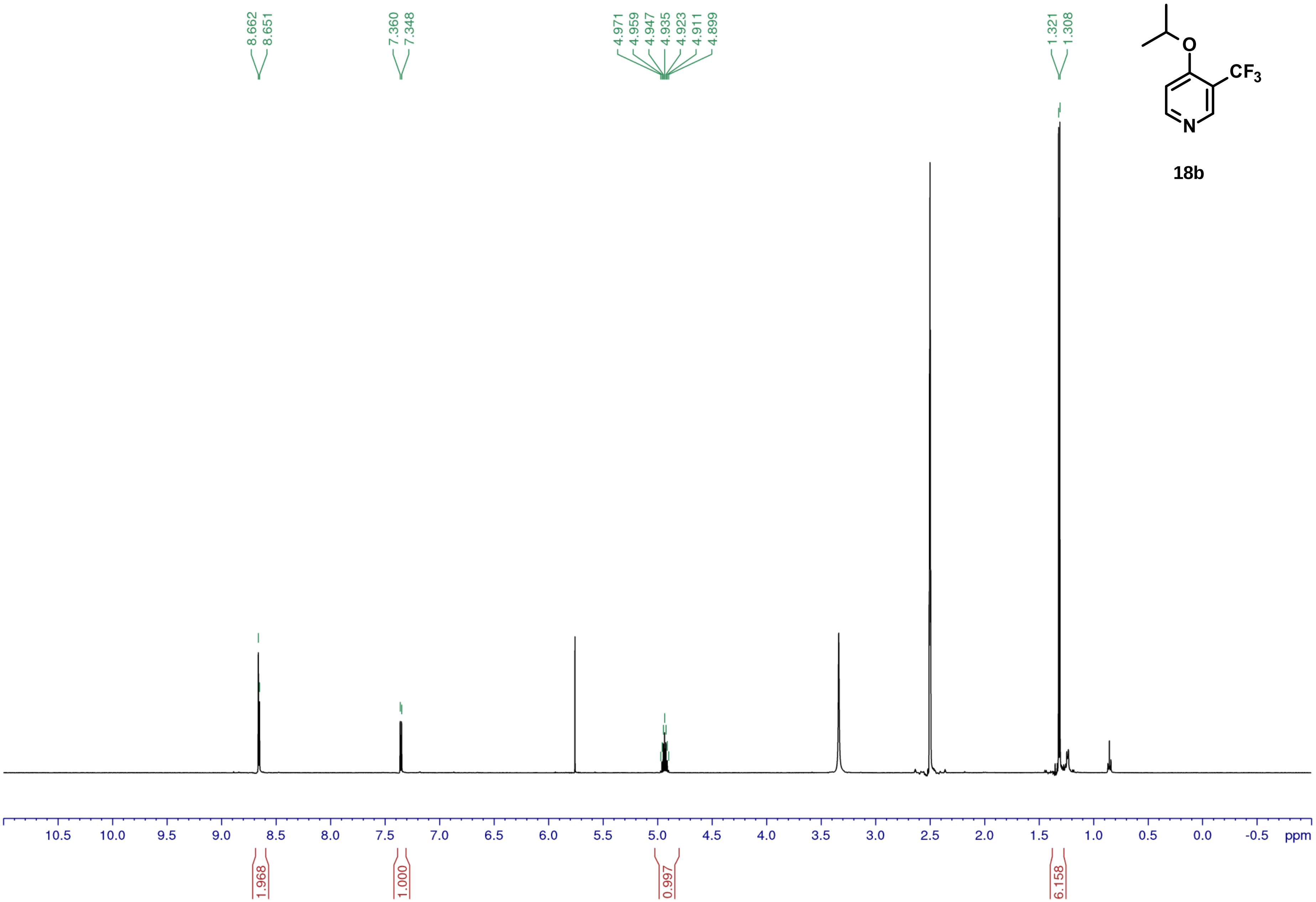
8.662
8.651
7.360
7.348
4.971
4.959
4.947
4.935
4.923
4.911
4.899
1.321
1.308
O
CF3
N
18b
10.5 10.0 9.5 9.0 8.5 8.0 7.5 7.0 6.5 6.0 5.5 5.0 4.5 4.0 3.5 3.0 2.5 2.0 1.5 1.0 0.5 0.0 -0.5 ppm
1.968
1.000
0.997
6.158

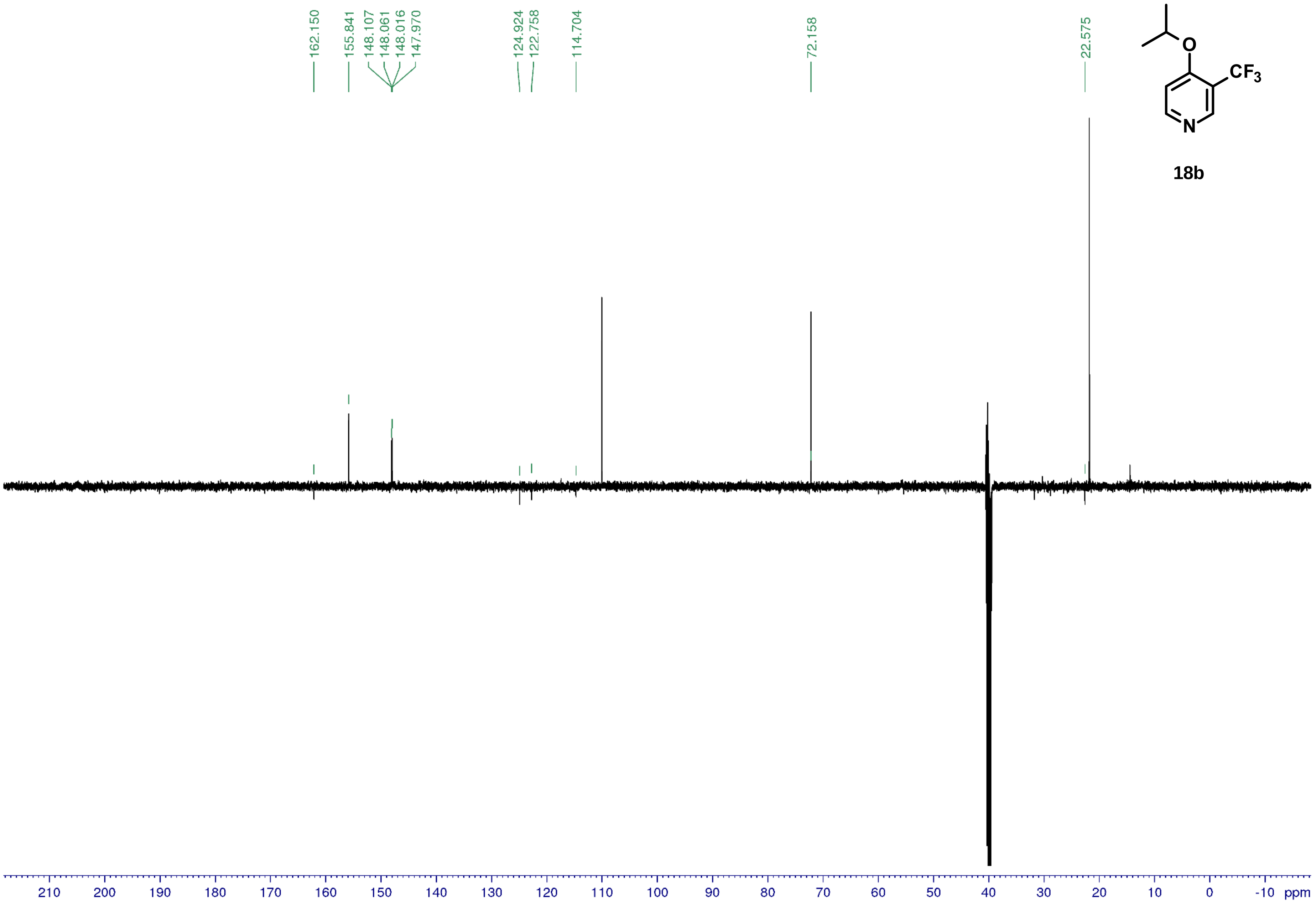
162.150
155.841
148.107
148.061
148.016
147.970
124.924
122.758
114.704
72.158
22.575
O
CF3
N
210
200
190
180
170
160
150
140
130
120
110
100
90
80
70
60
50
40
30
20
10
0
-10
ppm

18b

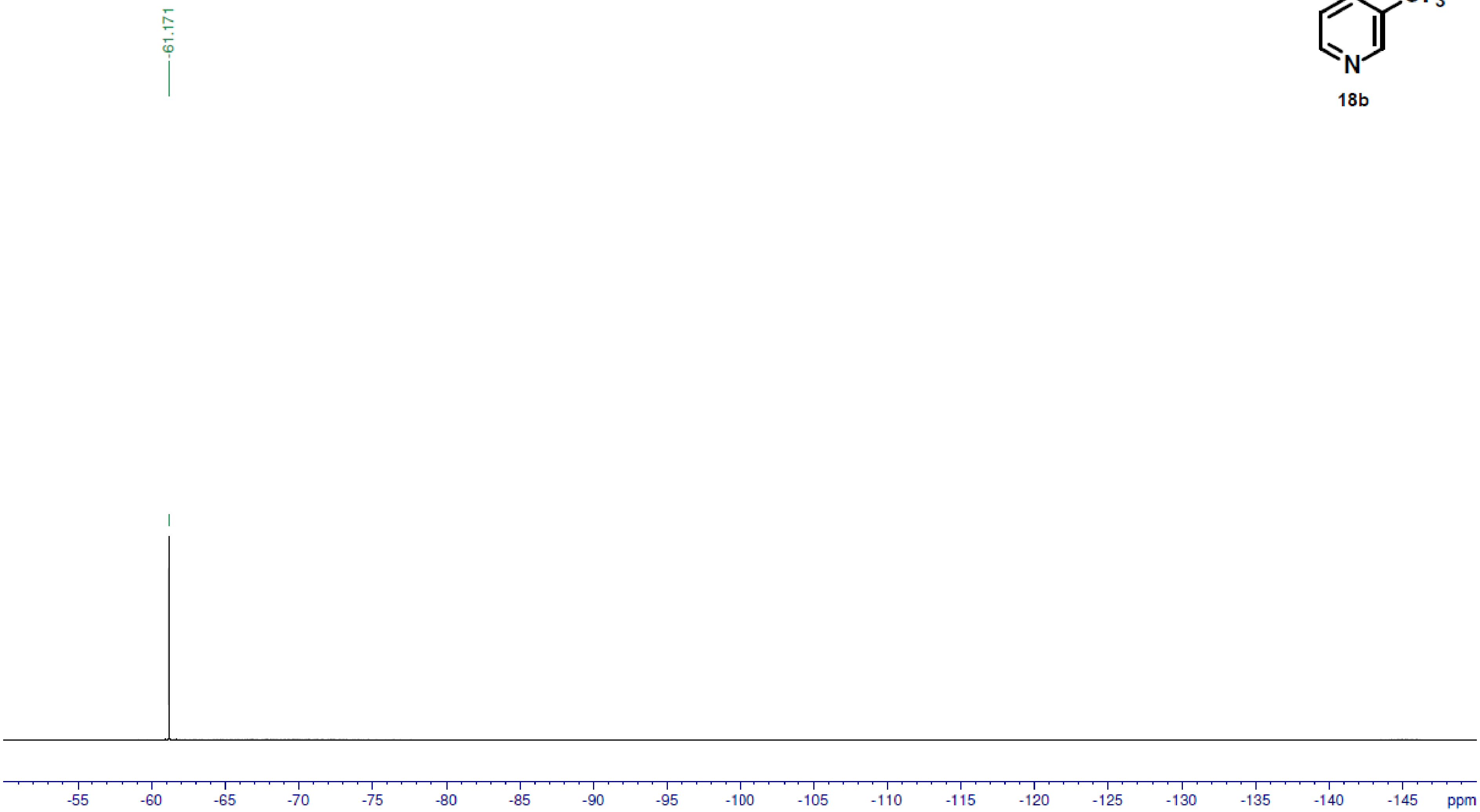
-61.171
-55
-60
-65
-70
-75
-80
-85
-90
-95
-100
-105
-110
-115
-120
-125
-130
-135
-140
-145
ppm

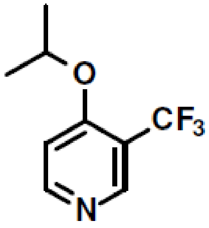
O
CF3
N

18b

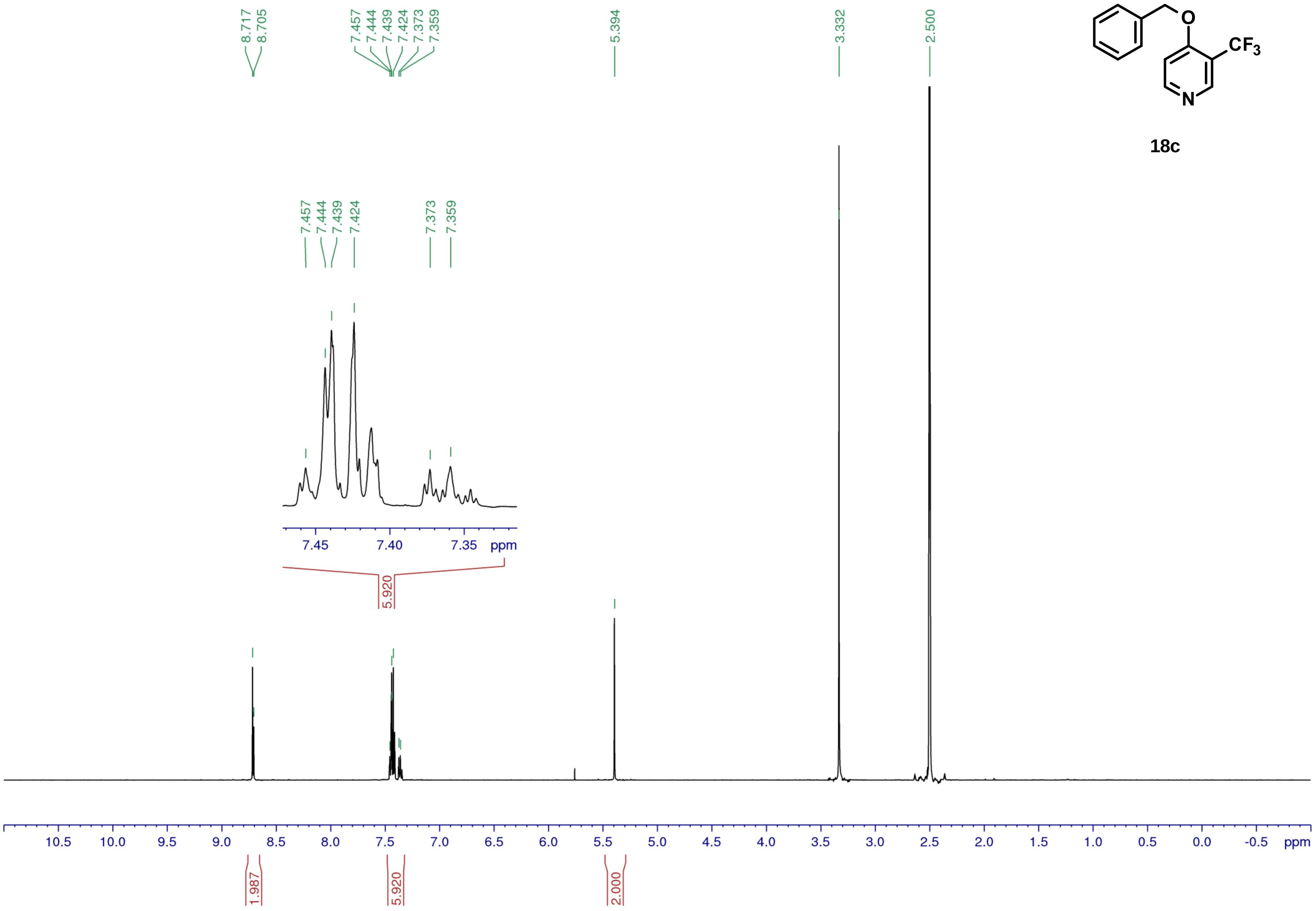

8.717
8.705
7.457
7.444
7.439
7.424
7.373
7.359
5.394
3.332
2.500
O
CF3
N
18c
7.45
7.40
7.35
ppm
5.920
10.5
10.0
9.5
9.0
8.5
8.0
7.5
7.0
6.5
6.0
5.5
5.0
4.5
4.0
3.5
3.0
2.5
2.0
1.5
1.0
0.5
0.0
-0.5
ppm
1.987
5.920
2.000

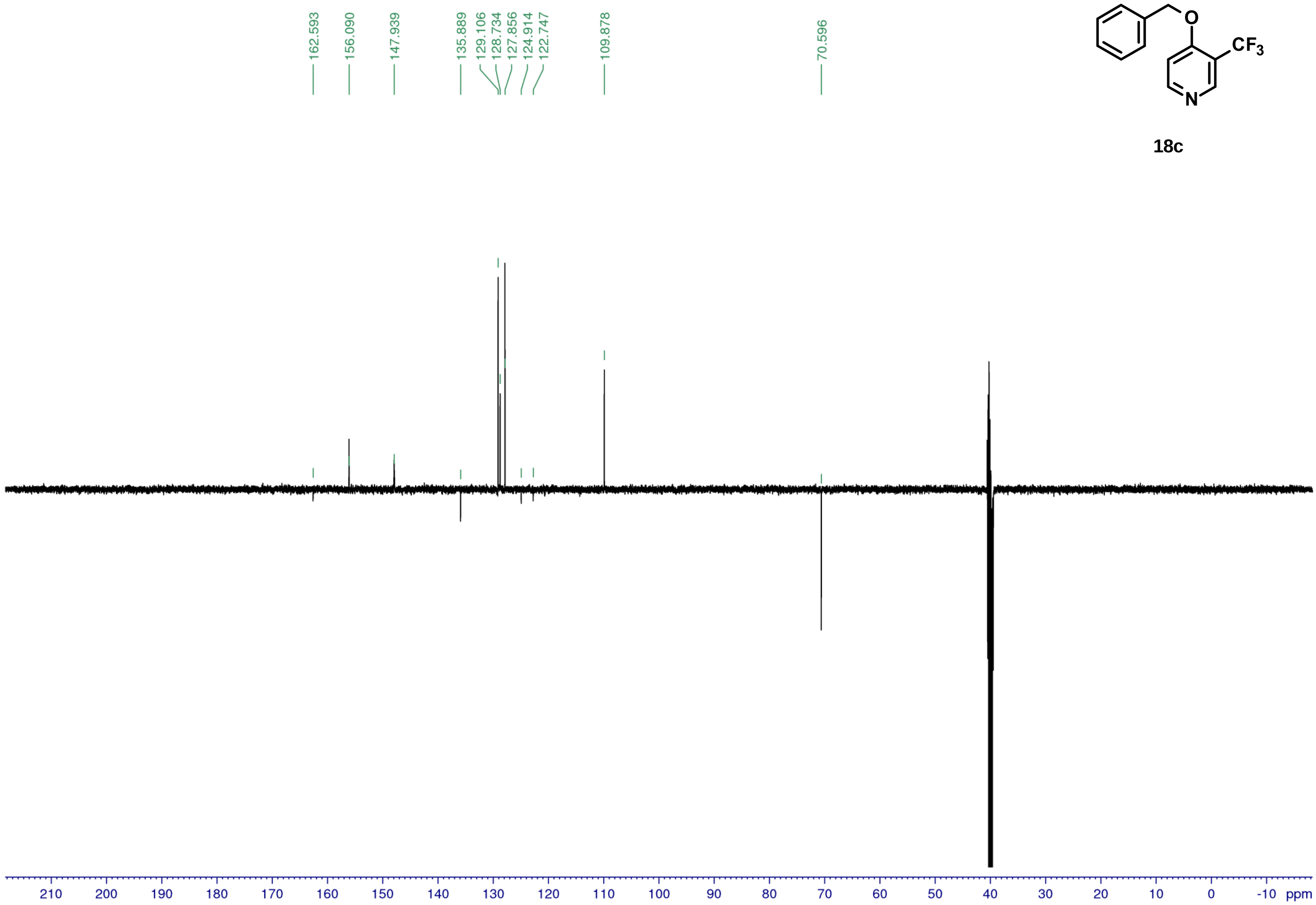

162.593
156.090
147.939
135.889
129.106
128.734
127.856
124.914
122.747
109.878
70.596
210
200
190
180
170
160
150
140
130
120
110
100
90
80
70
60
50
40
30
20
10
0
-10
ppm

18c

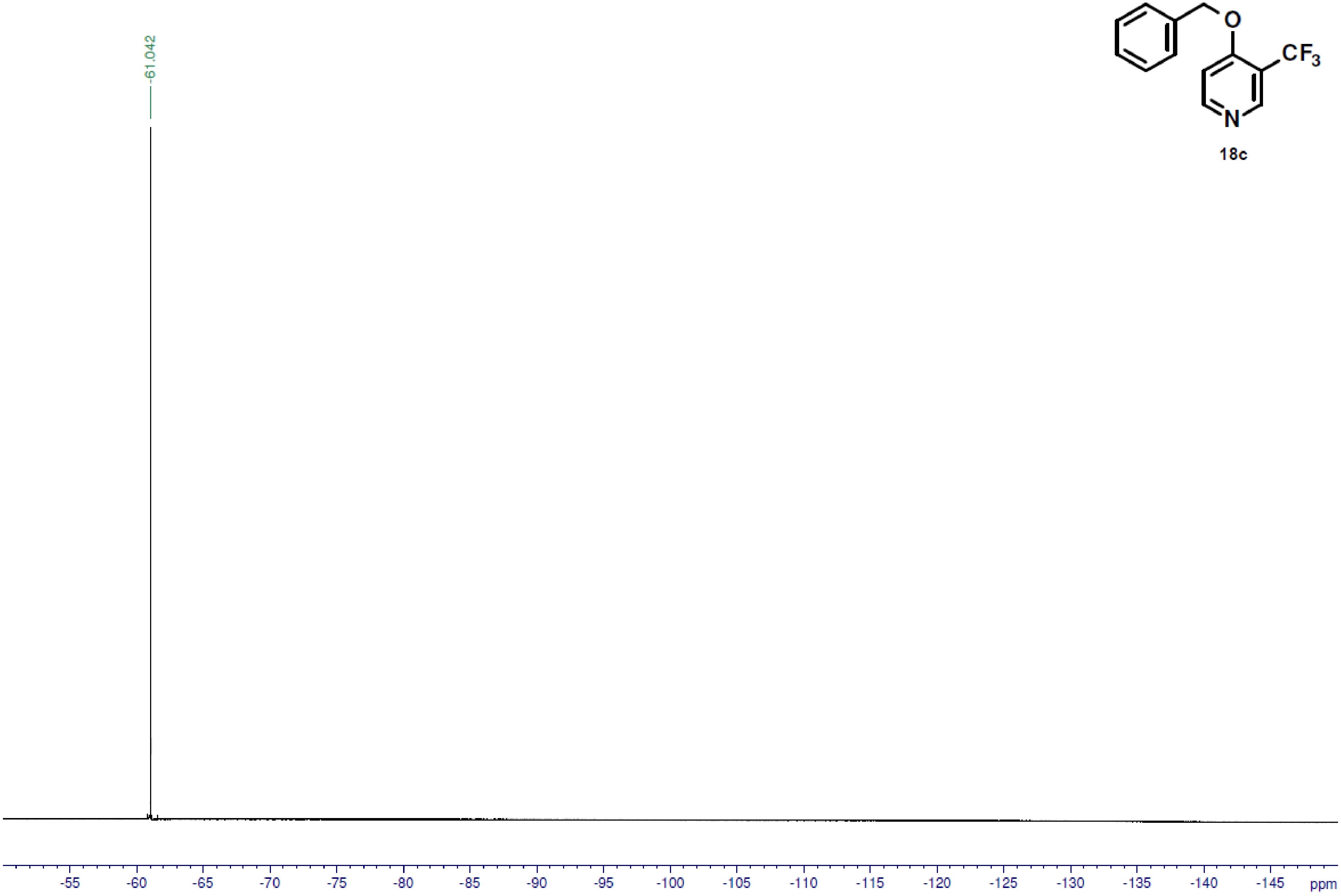
-61.042
-55
-60
-65
-70
-75
-80
-85
-90
-95
-100
-105
-110
-115
-120
-125
-130
-135
-140
-145
ppm
O
CF_3
N

18c

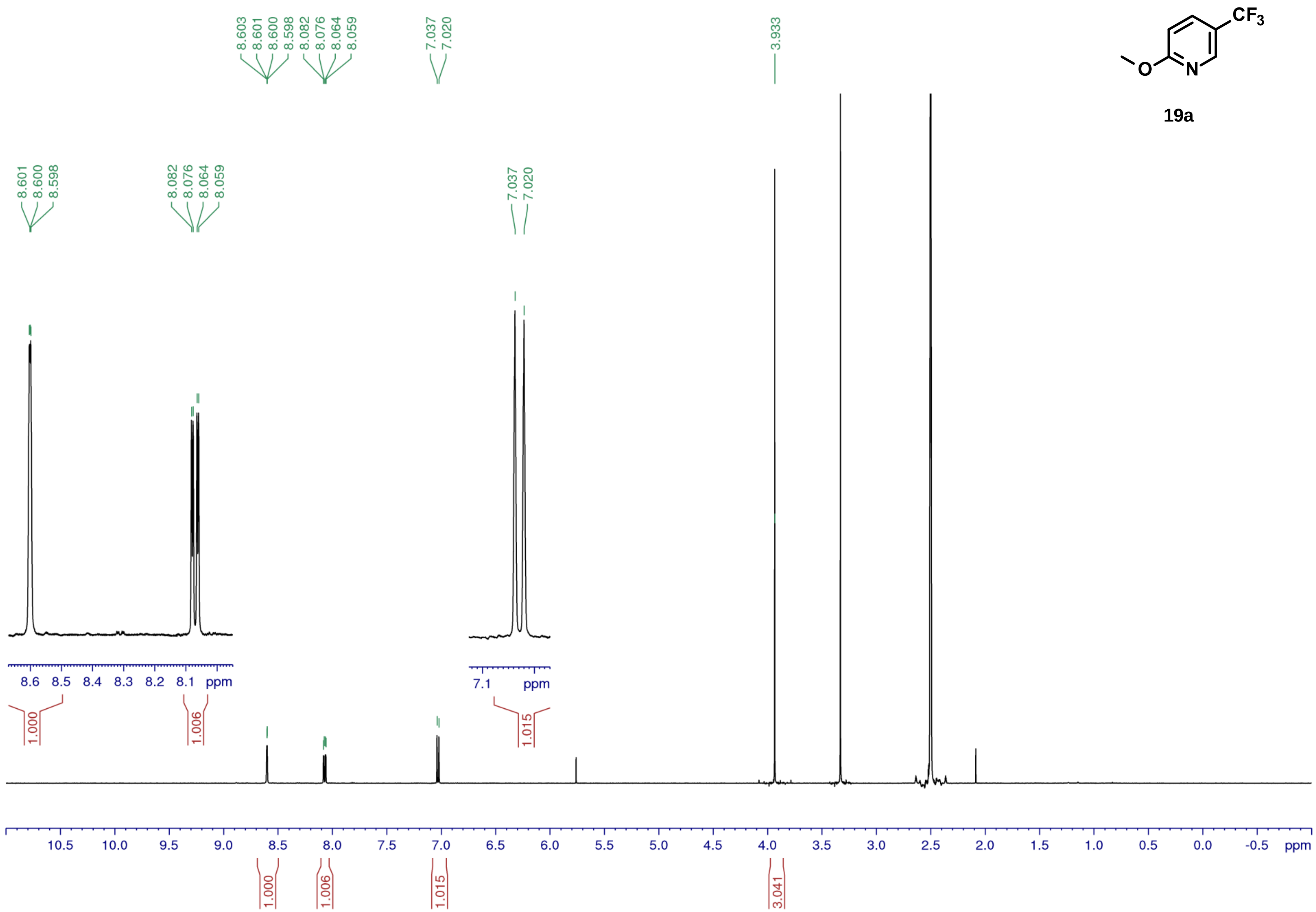

8.603
8.601
8.600
8.598
8.082
8.076
8.064
8.059
7.037
7.020
3.933
8.601
8.600
8.598
8.082
8.076
8.064
8.059
7.037
7.020
8.6 8.5 8.4 8.3 8.2 8.1 ppm
7.1 ppm
1.000
1.006
1.015
10.5 10.0 9.5 9.0 8.5 8.0 7.5 7.0 6.5 6.0 5.5 5.0 4.5 4.0 3.5 3.0 2.5 2.0 1.5 1.0 0.5 0.0 -0.5 ppm
1.000
1.006
1.015
3.041
CF3
O
N

19a

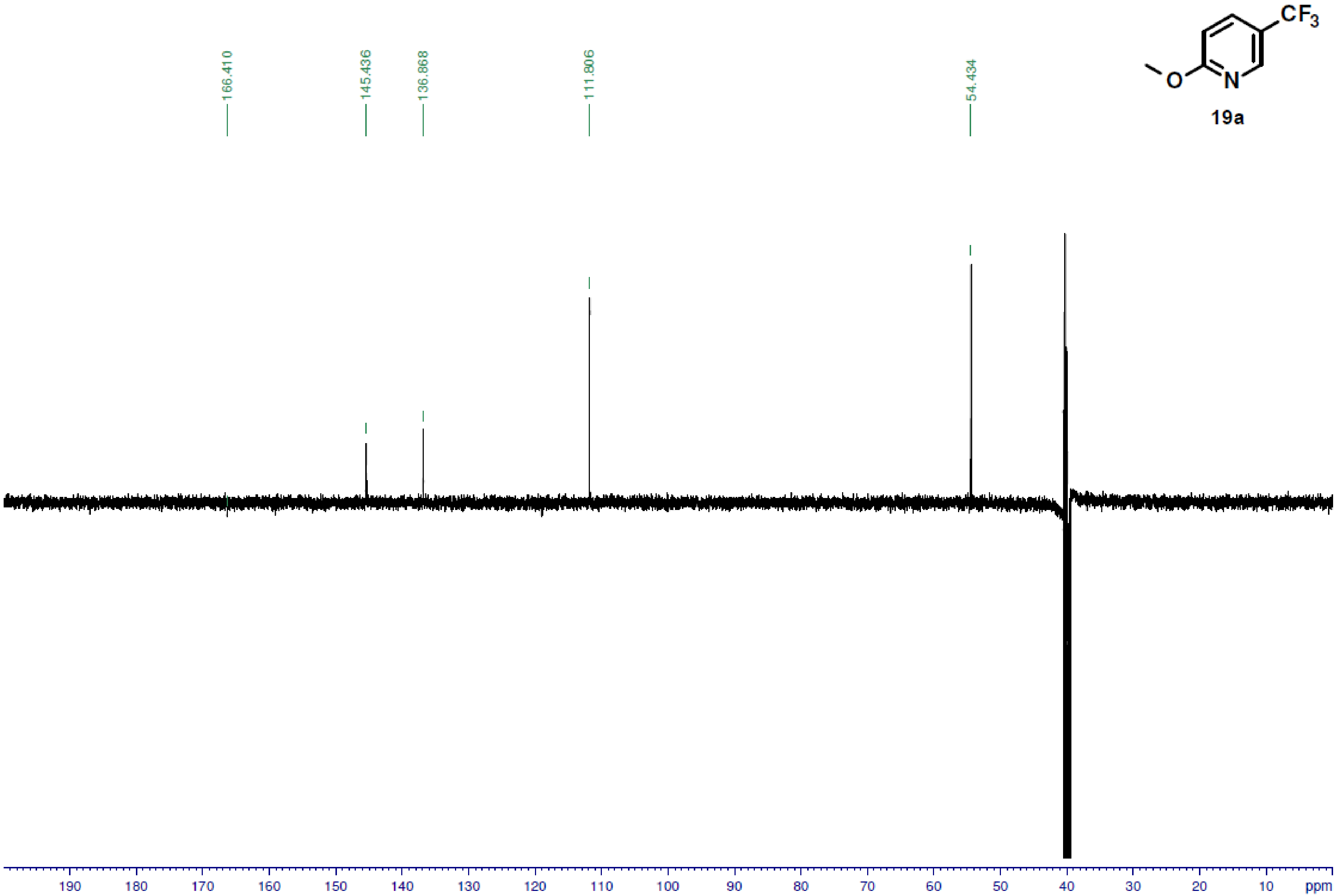

166.410
145.436
136.868
111.806
54.434
CF3
O
N
19a
190
180
170
160
150
140
130
120
110
100
90
80
70
60
50
40
30
20
10
ppm

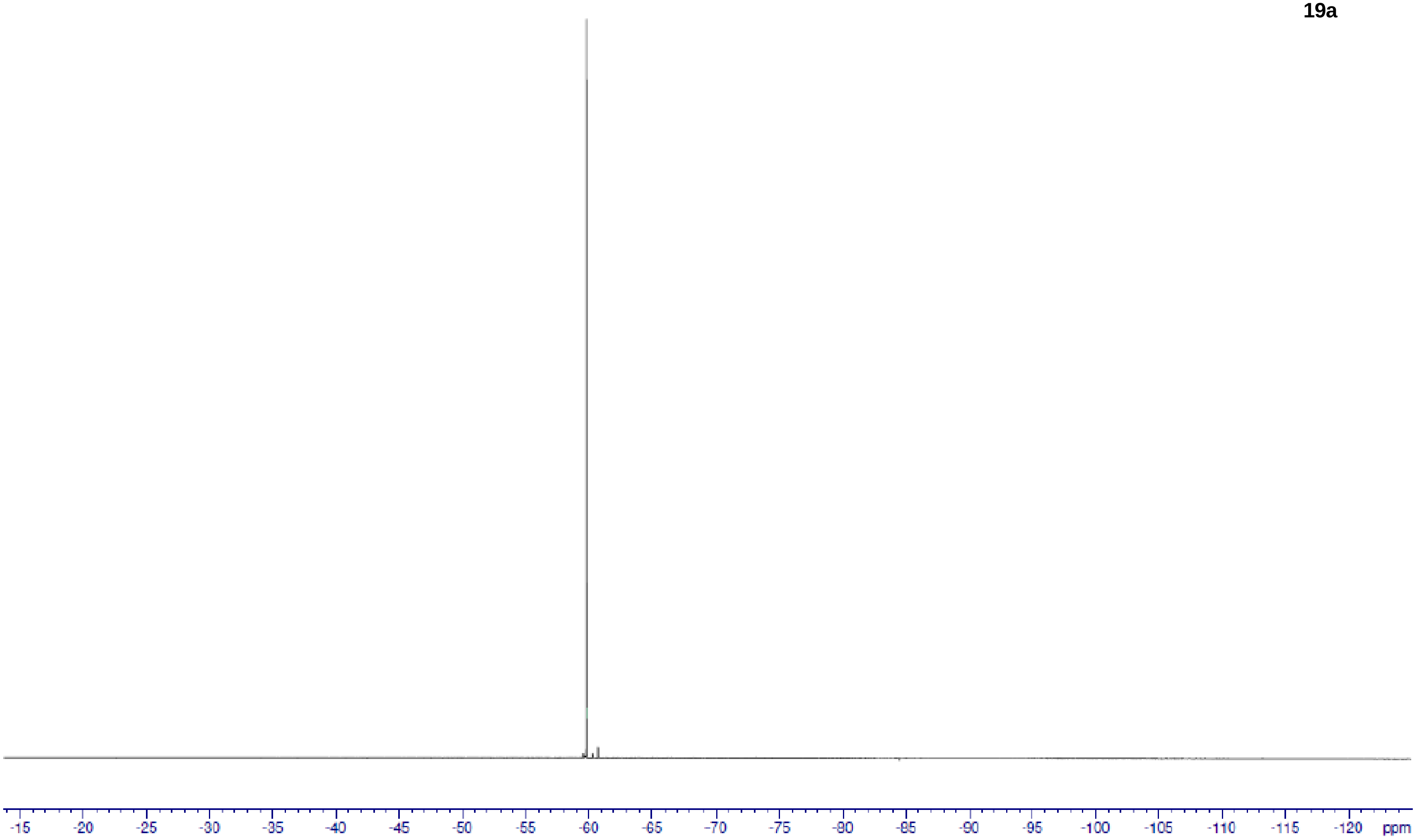
-59.850
-15
-20
-25
-30
-35
-40
-45
-50
-55
-60
-65
-70
-75
-80
-85
-90
-95
-100
-105
-110
-115
-120
ppm

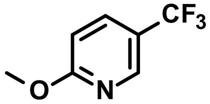
CF3
O
N

19a

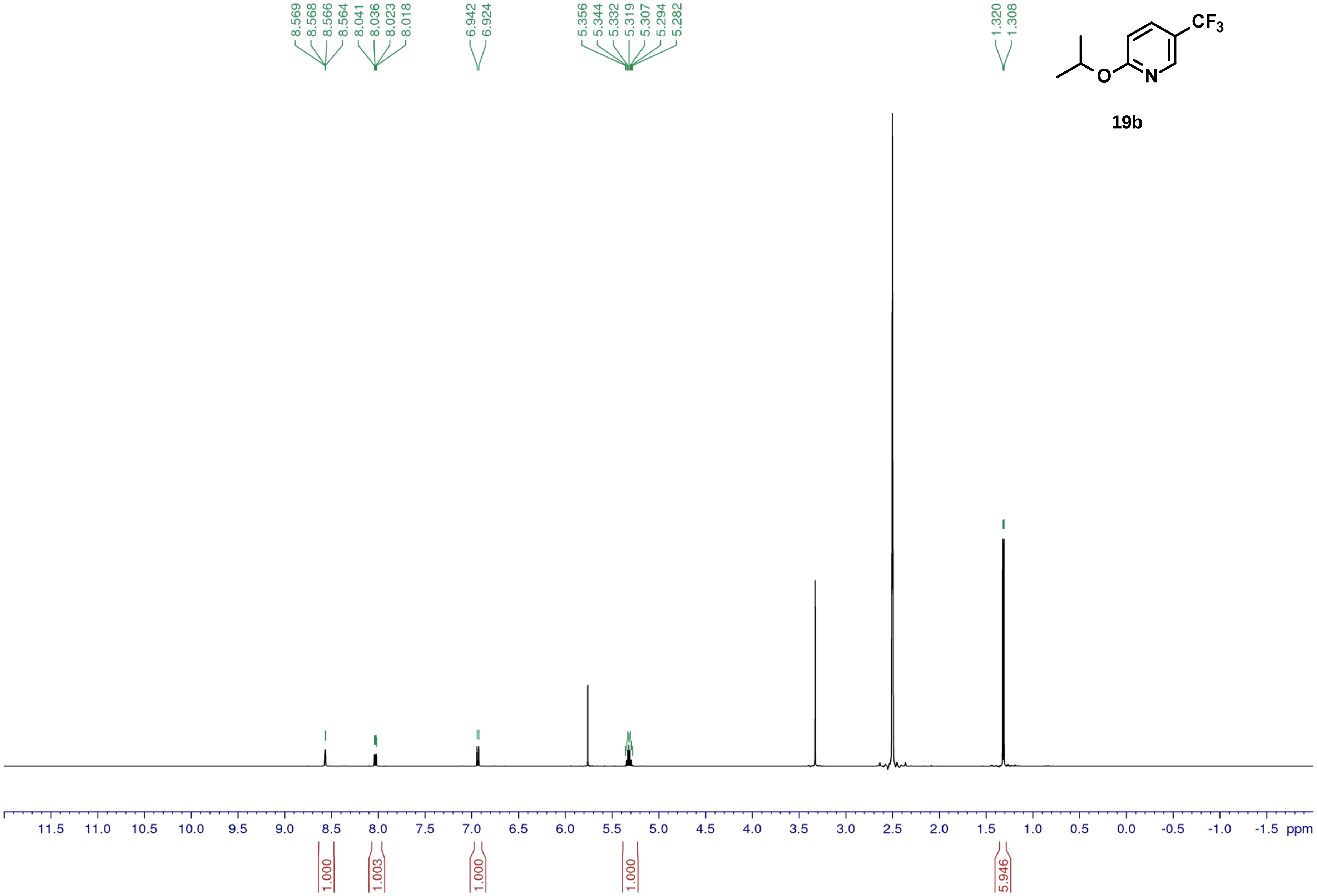
8.569
8.568
8.566
8.564
8.041
8.036
8.023
8.018
6.942
6.924
5.356
5.344
5.332
5.319
5.307
5.294
5.282
1.320
1.308
CF3
O
N
11.5 11.0 10.5 10.0 9.5 9.0 8.5 8.0 7.5 7.0 6.5 6.0 5.5 5.0 4.5 4.0 3.5 3.0 2.5 2.0 1.5 1.0 0.5 0.0 -0.5 -1.0 -1.5 ppm
1.000
1.003
1.000
1.000
5.946

19b

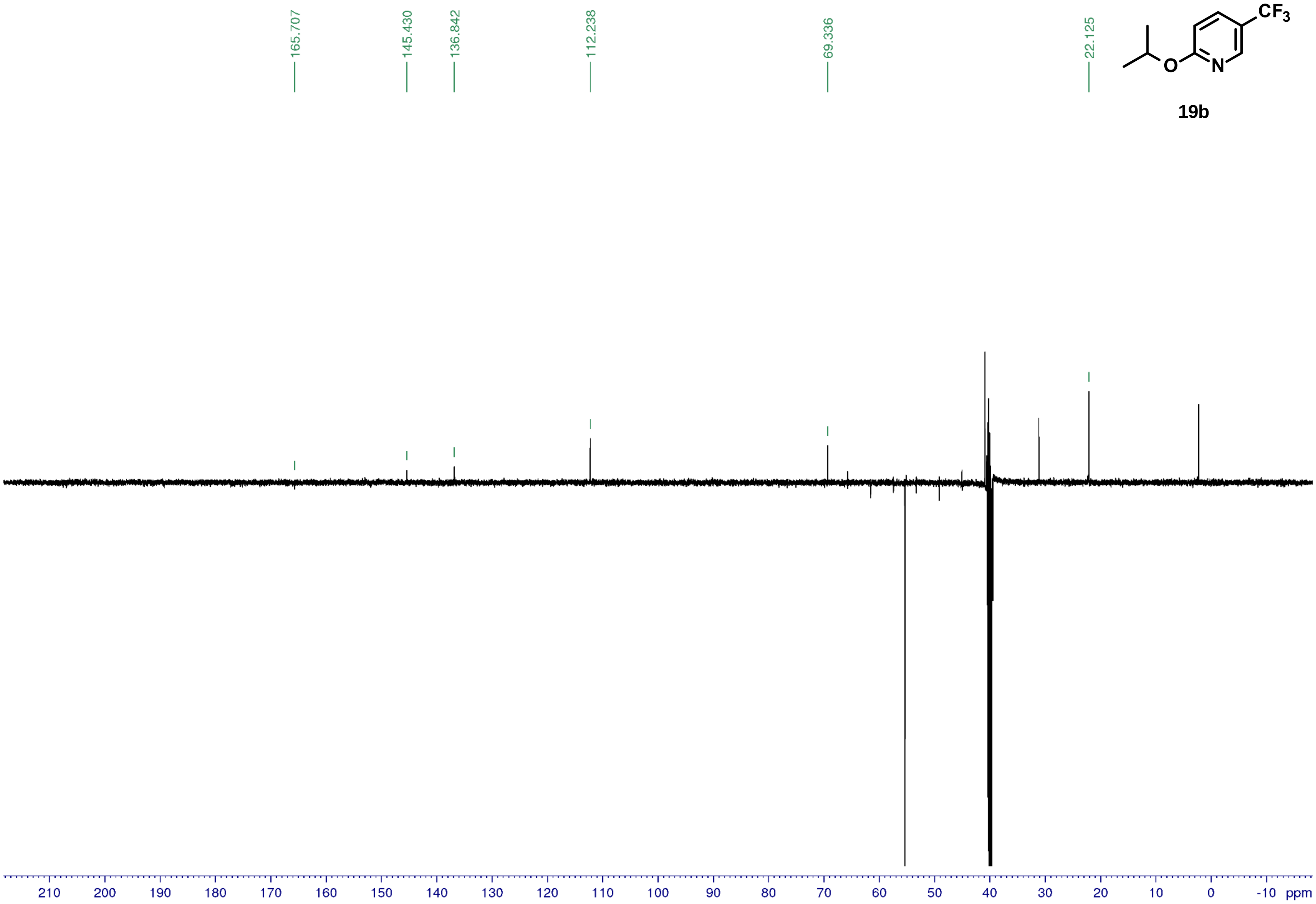
165.707
145.430
136.842
112.238
69.336
22.125
210
200
190
180
170
160
150
140
130
120
110
100
90
80
70
60
50
40
30
20
10
0
-10
ppm
CF3
O
N

19b

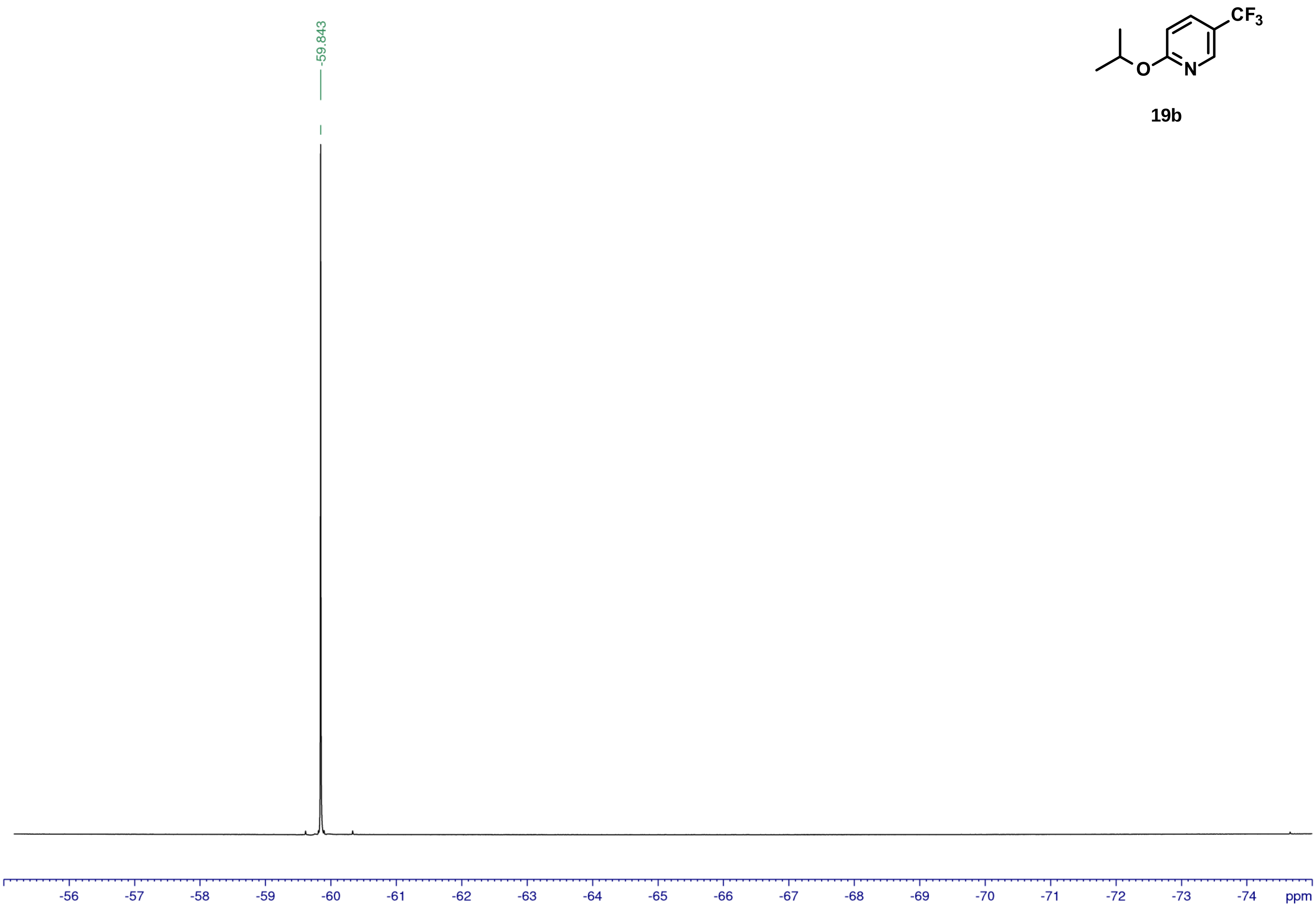

-59.843
CF3
O
N
19b
-56
-57
-58
-59
-60
-61
-62
-63
-64
-65
-66
-67
-68
-69
-70
-71
-72
-73
-74
ppm

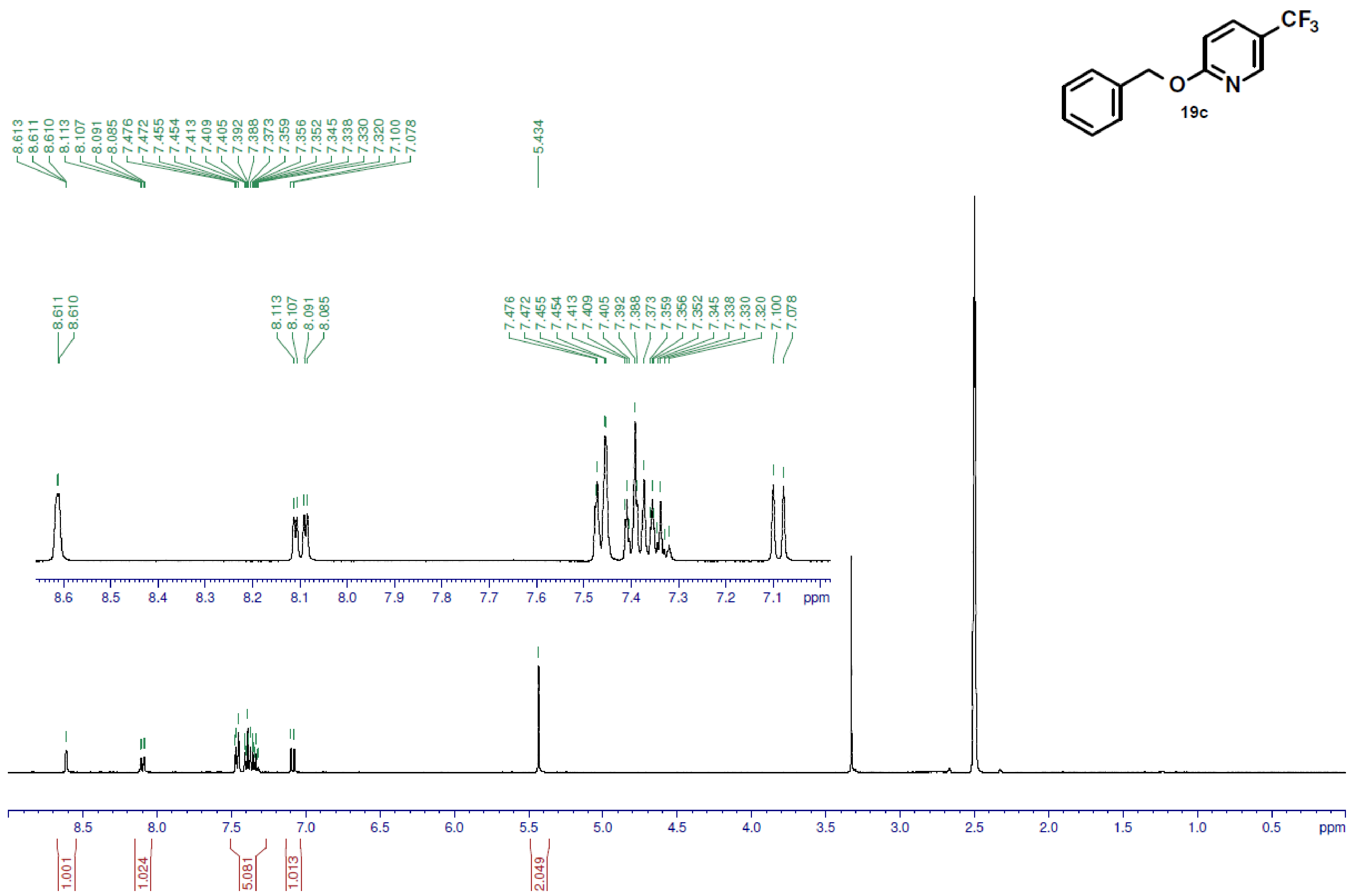

CF3
O
N
19c
8.613
8.611
8.610
8.113
8.107
8.091
8.085
7.476
7.472
7.455
7.454
7.413
7.409
7.405
7.392
7.388
7.373
7.359
7.356
7.352
7.345
7.338
7.330
7.320
7.100
7.078
5.434
ppm
1.001
1.024
5.081
1.013
2.049

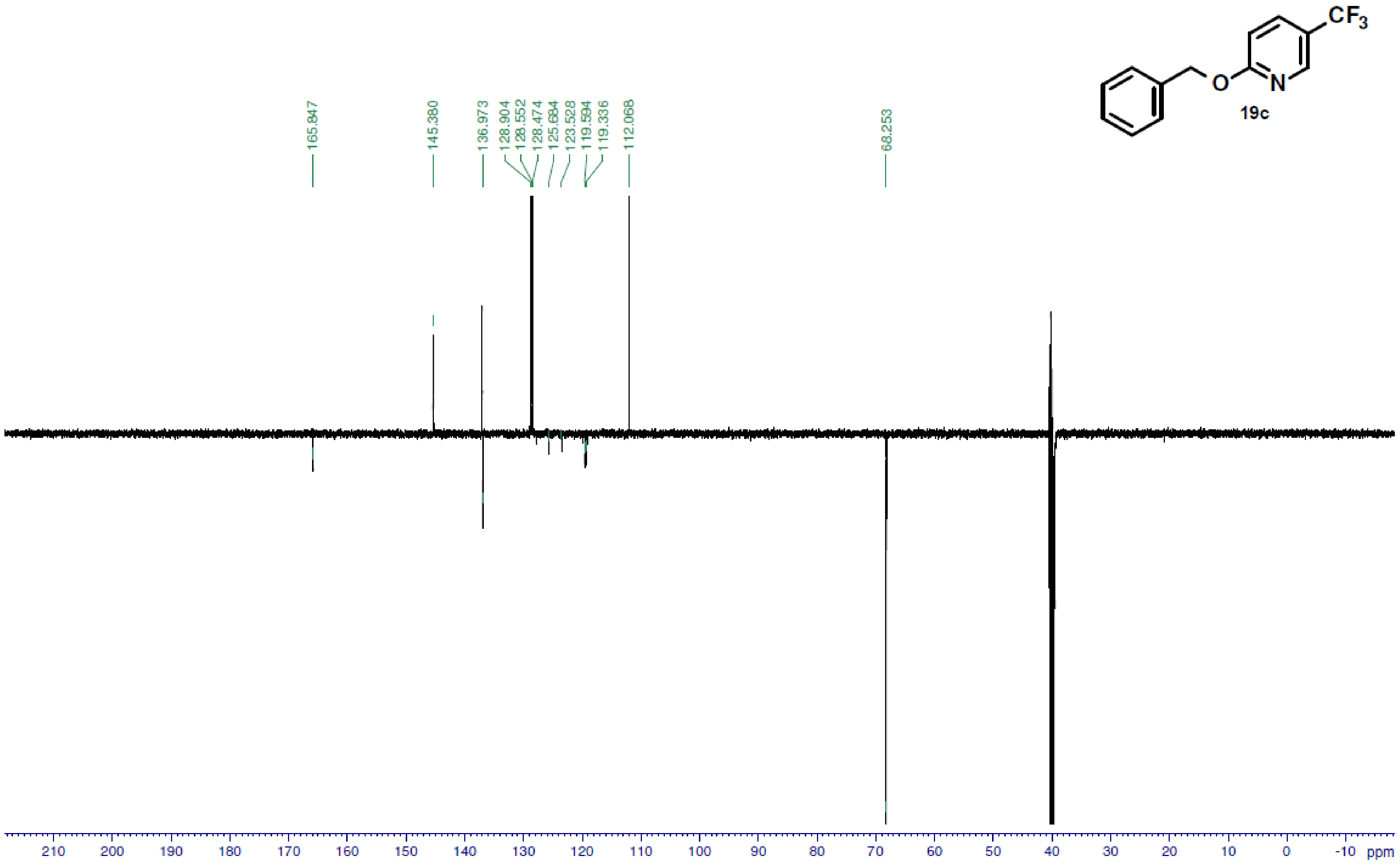

CF3
O
N
19c
165.847
145.380
136.973
128.904
128.552
128.474
125.684
123.528
119.594
119.336
112.068
68.253
210
200
190
180
170
160
150
140
130
120
110
100
90
80
70
60
50
40
30
20
10
0
-10
ppm

-59.867

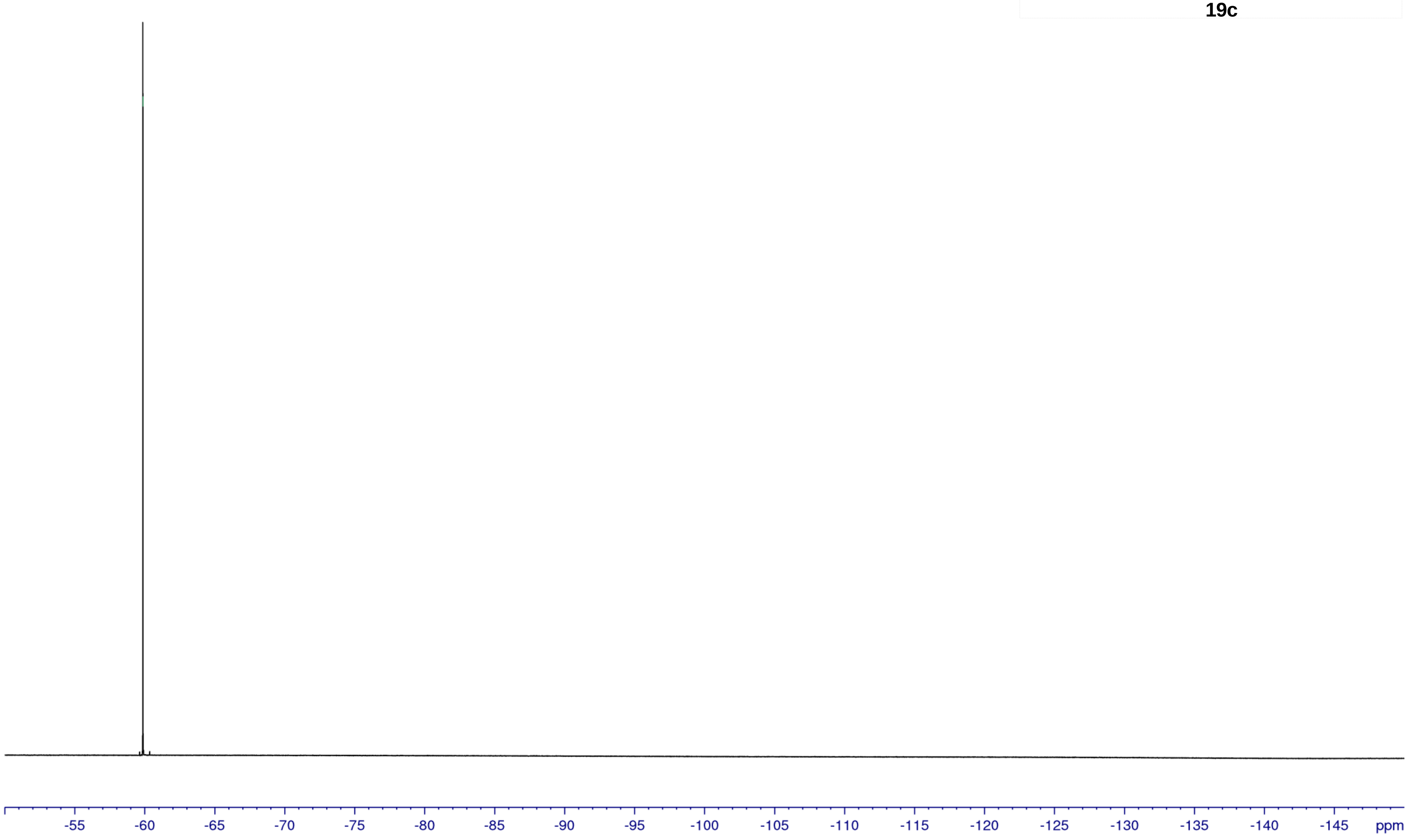

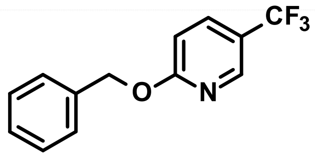

19c

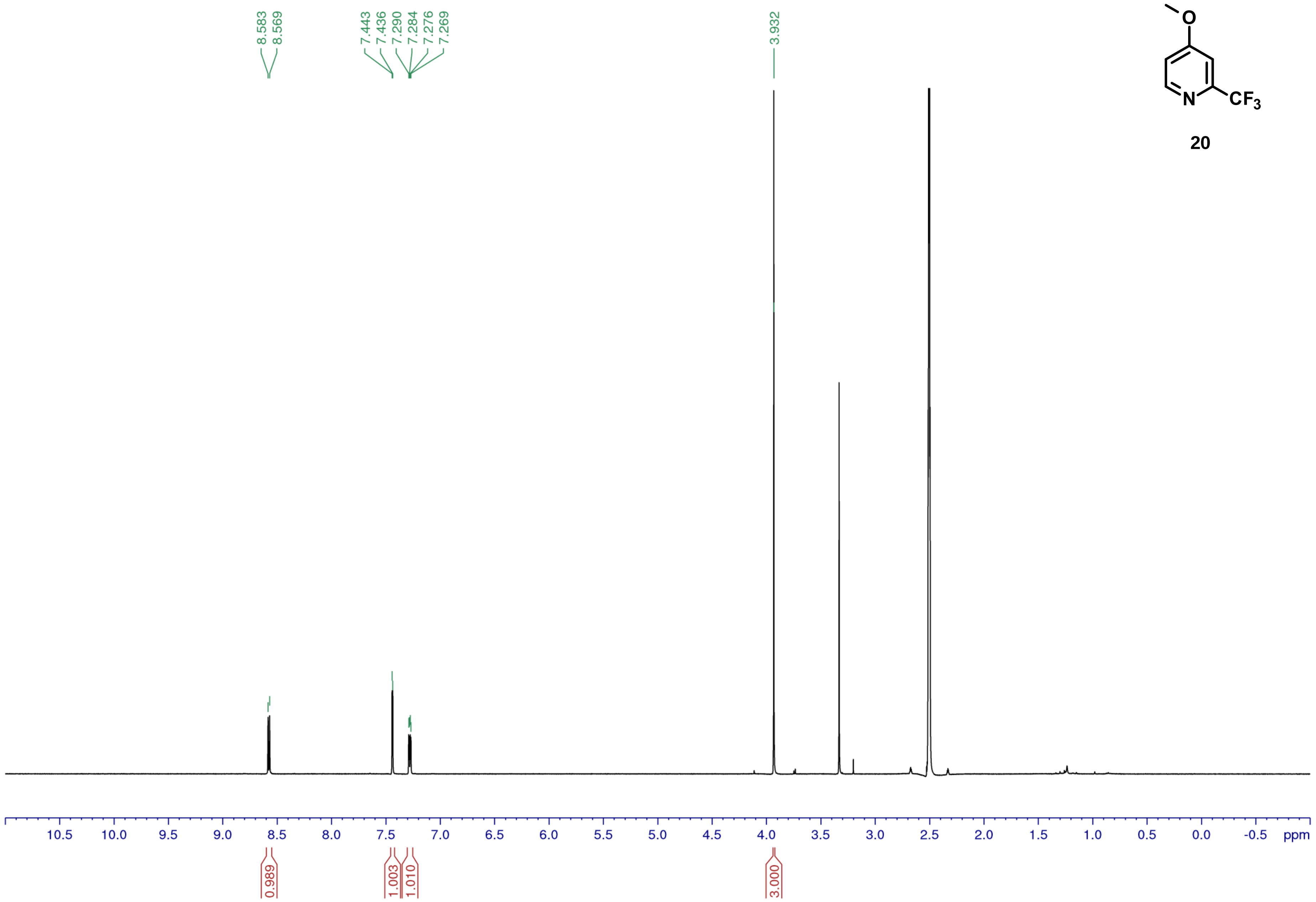
8.583
8.569
7.443
7.436
7.290
7.284
7.276
7.269
3.932
10.5 10.0 9.5 9.0 8.5 8.0 7.5 7.0 6.5 6.0 5.5 5.0 4.5 4.0 3.5 3.0 2.5 2.0 1.5 1.0 0.5 0.0 -0.5 ppm
0.989
1.003
1.010
3.000
O
N
CF3

20

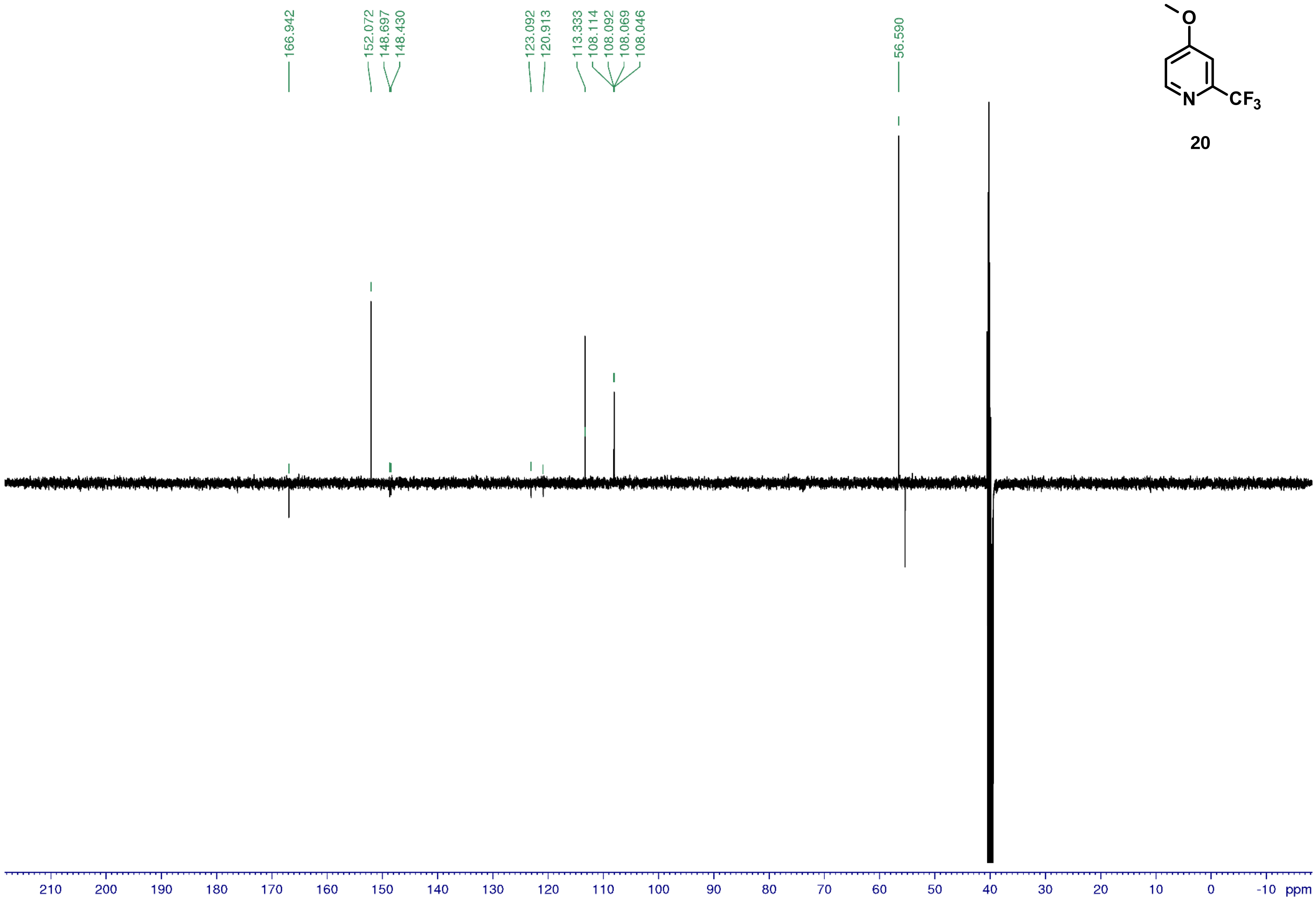
166.942
152.072
148.697
148.430
123.092
120.913
113.333
108.114
108.092
108.069
108.046
56.590
O
N
CF3
20
210
200
190
180
170
160
150
140
130
120
110
100
90
80
70
60
50
40
30
20
10
0
-10
ppm

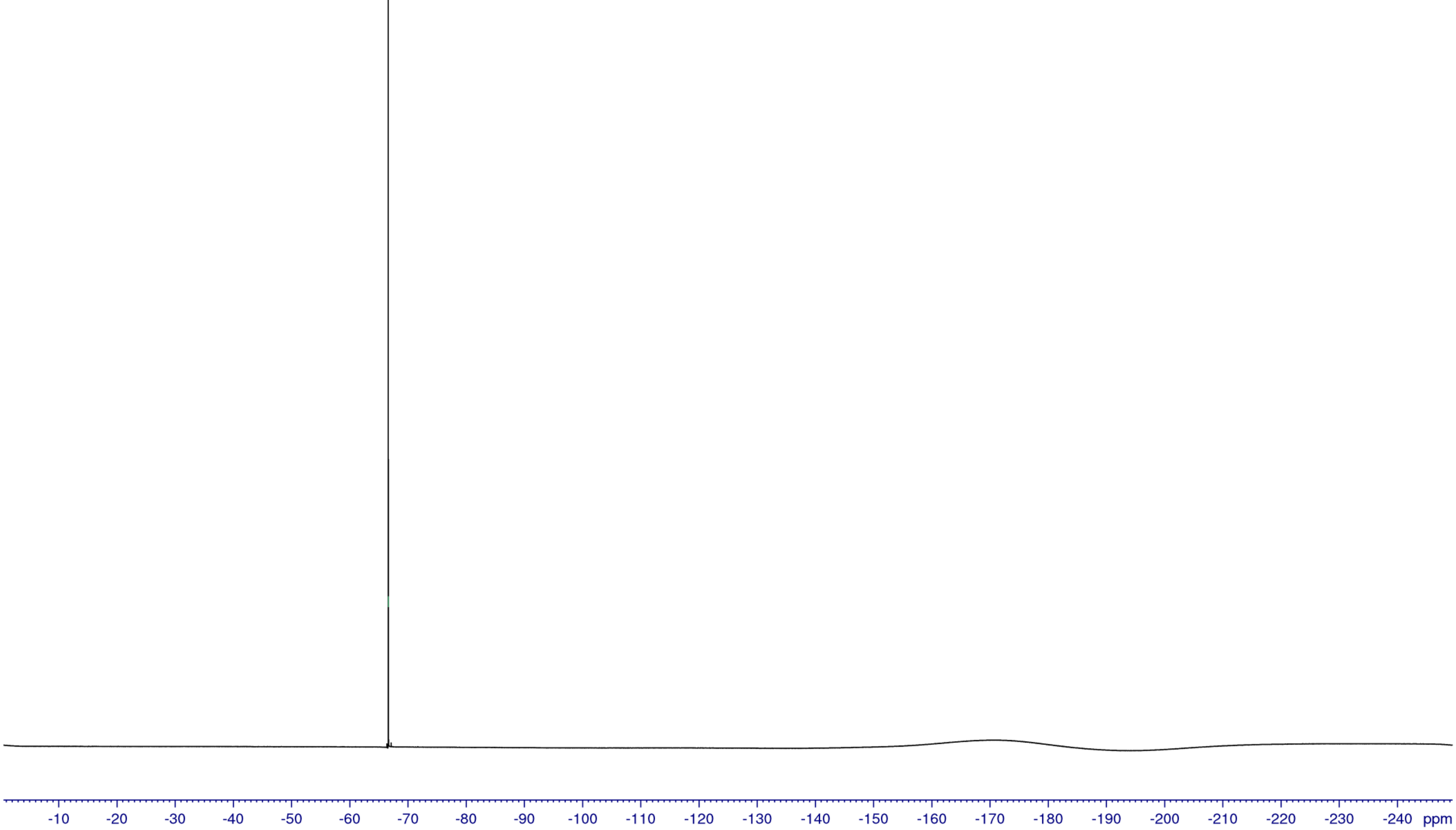
-66.627
-10 -20 -30 -40 -50 -60 -70 -80 -90 -100 -110 -120 -130 -140 -150 -160 -170 -180 -190 -200 -210 -220 -230 -240 ppm

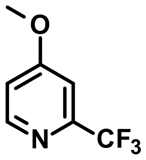
O
N
CF3

20

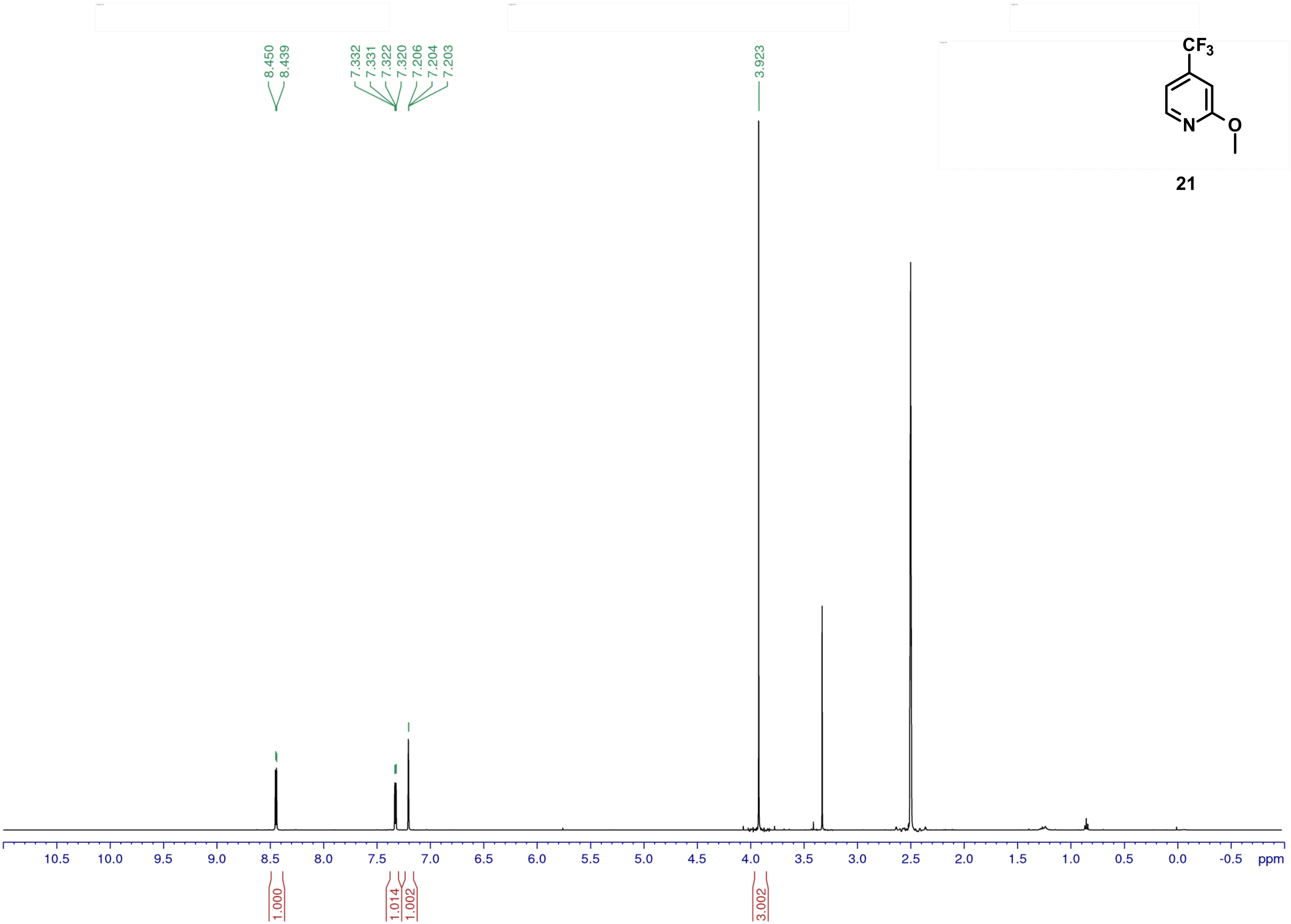
8.450
8.439
7.332
7.331
7.322
7.320
7.206
7.204
7.203
3.923
CF3
N
O
10.5 10.0 9.5 9.0 8.5 8.0 7.5 7.0 6.5 6.0 5.5 5.0 4.5 4.0 3.5 3.0 2.5 2.0 1.5 1.0 0.5 0.0 -0.5 ppm
1.000
1.014
1.002
3.002
21

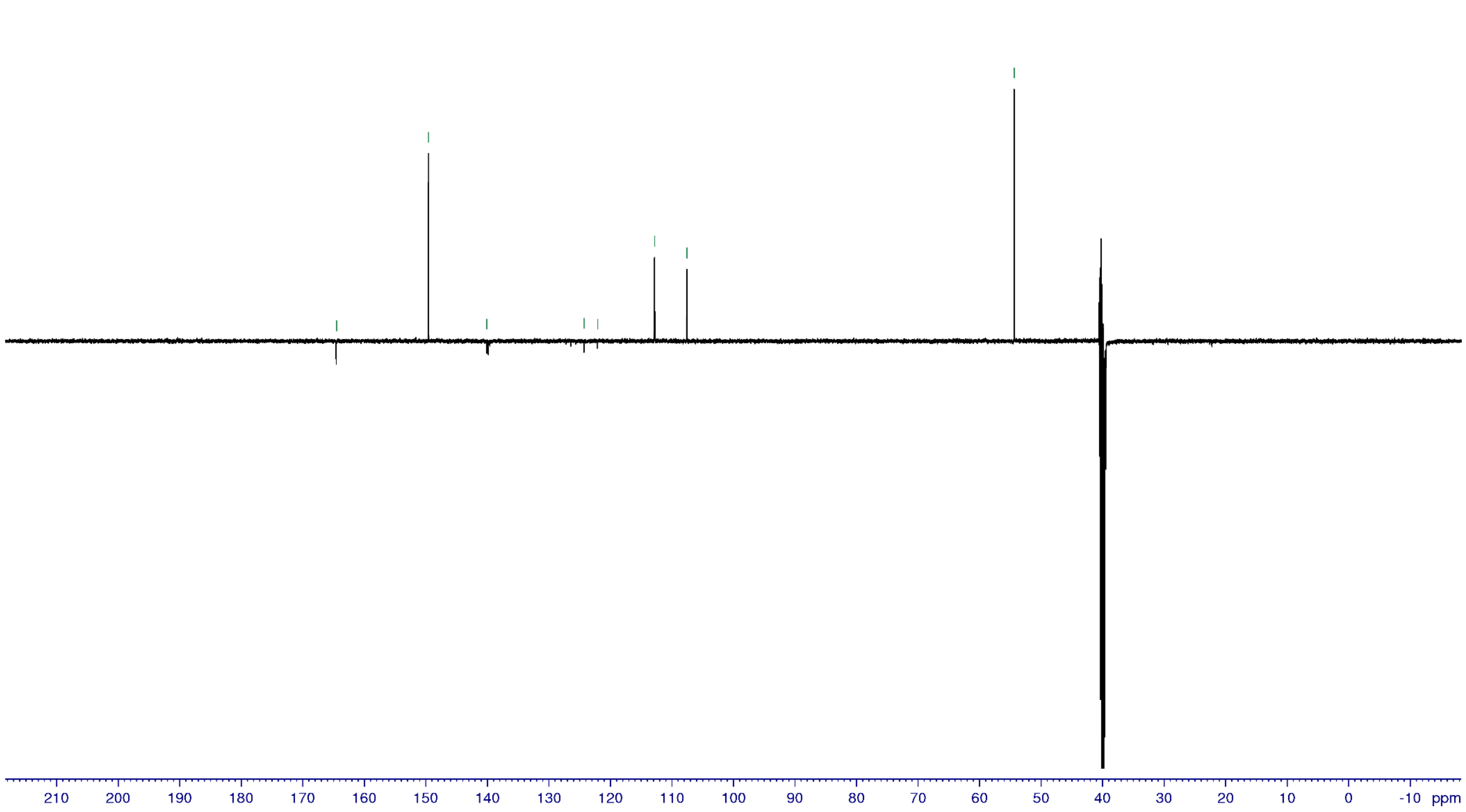

164.536
149.548
140.109
124.264
122.091
112.815
107.567
54.363
210 200 190 180 170 160 150 140 130 120 110 100 90 80 70 60 50 40 30 20 10 0 -10 ppm

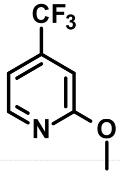

CF3
N
O

21

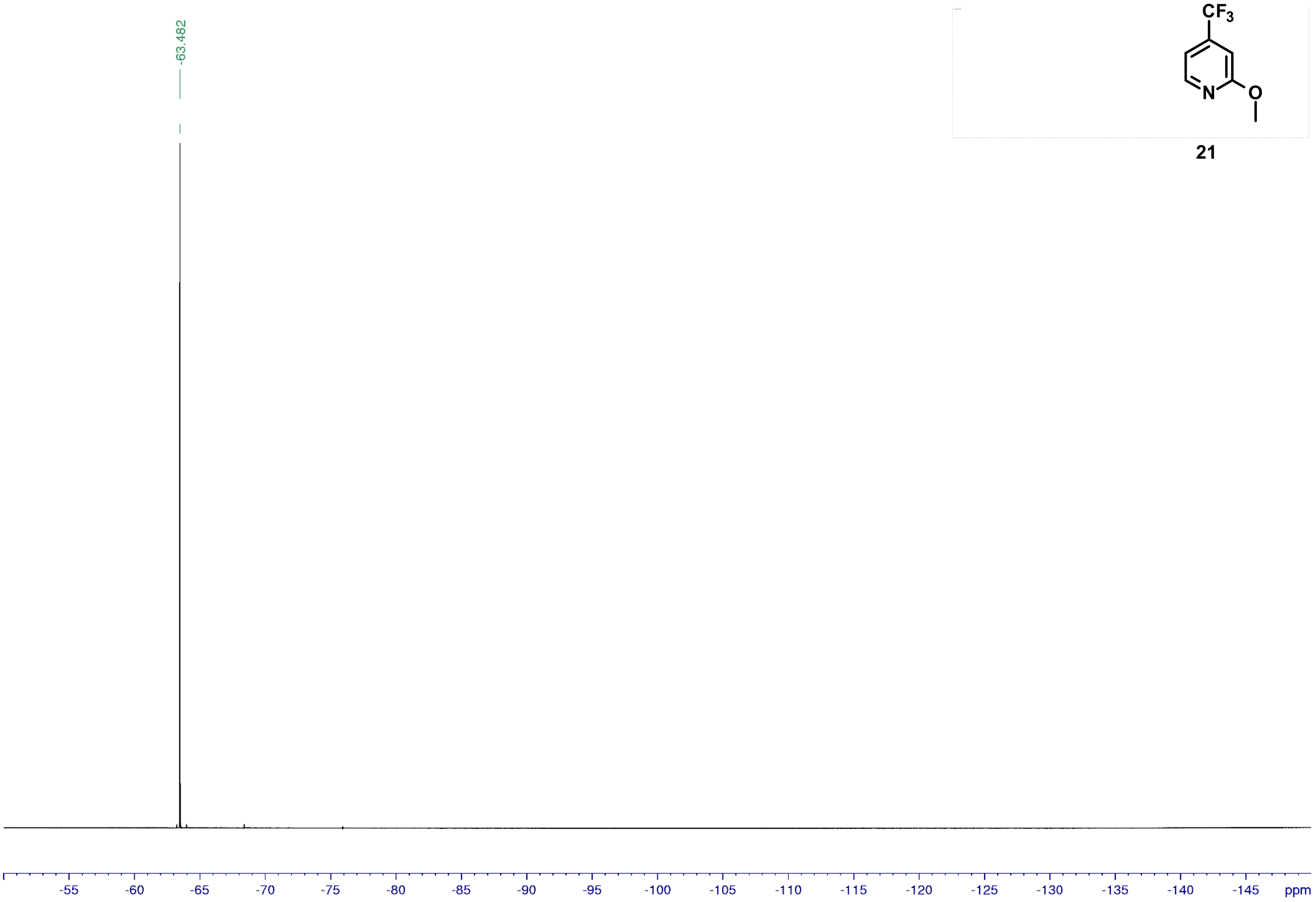
-63.482
-55
-60
-65
-70
-75
-80
-85
-90
-95
-100
-105
-110
-115
-120
-125
-130
-135
-140
-145
ppm
CF3
N
O

21

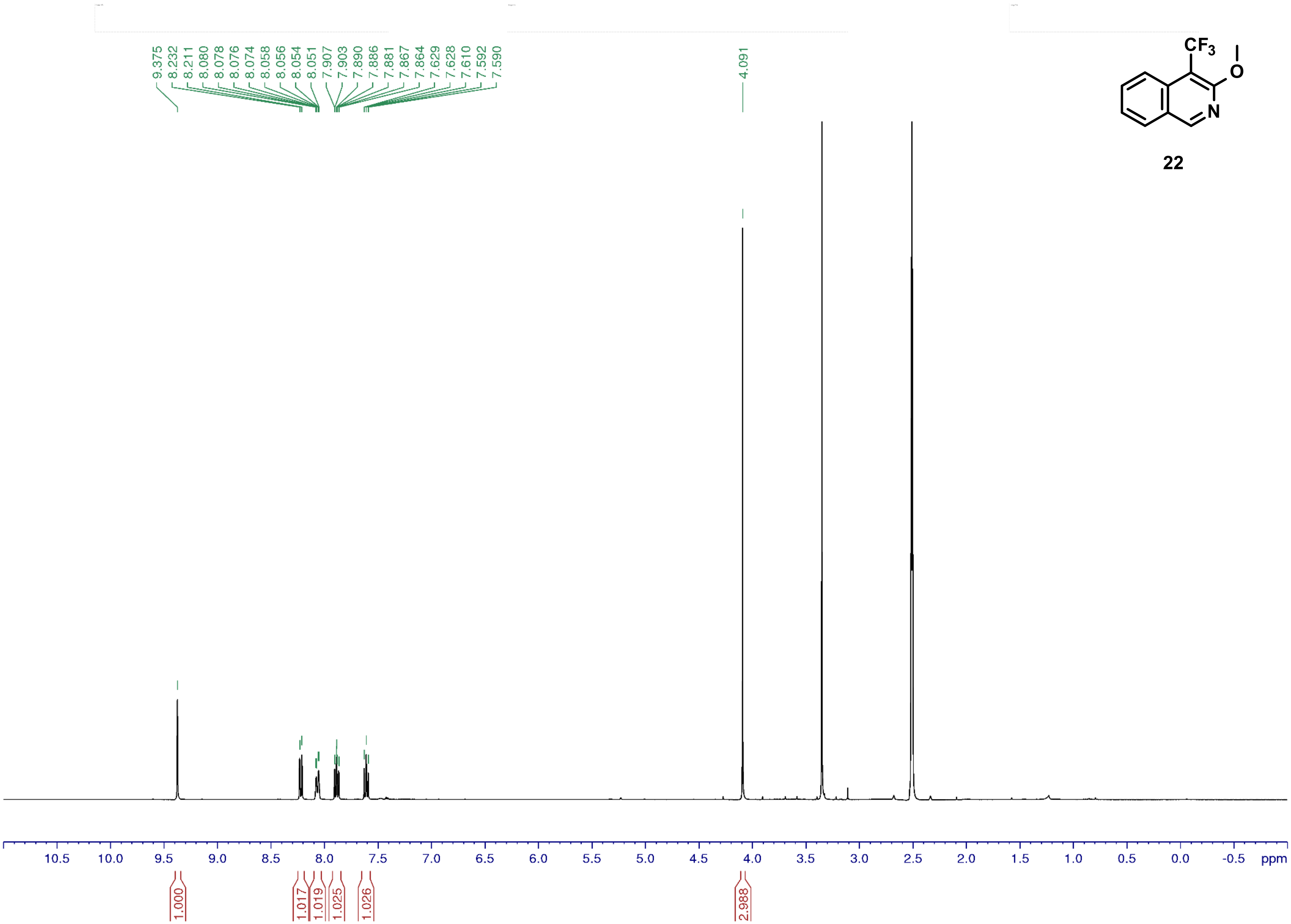
9.375
8.232
8.211
8.080
8.078
8.076
8.074
8.058
8.056
8.054
8.051
7.907
7.903
7.890
7.886
7.881
7.867
7.864
7.629
7.628
7.610
7.592
7.590
4.091
10.5
10.0
9.5
9.0
8.5
8.0
7.5
7.0
6.5
6.0
5.5
5.0
4.5
4.0
3.5
3.0
2.5
2.0
1.5
1.0
0.5
0.0
-0.5
ppm
1.000
1.017
1.019
1.025
1.026
2.988
CF3
O
N

22

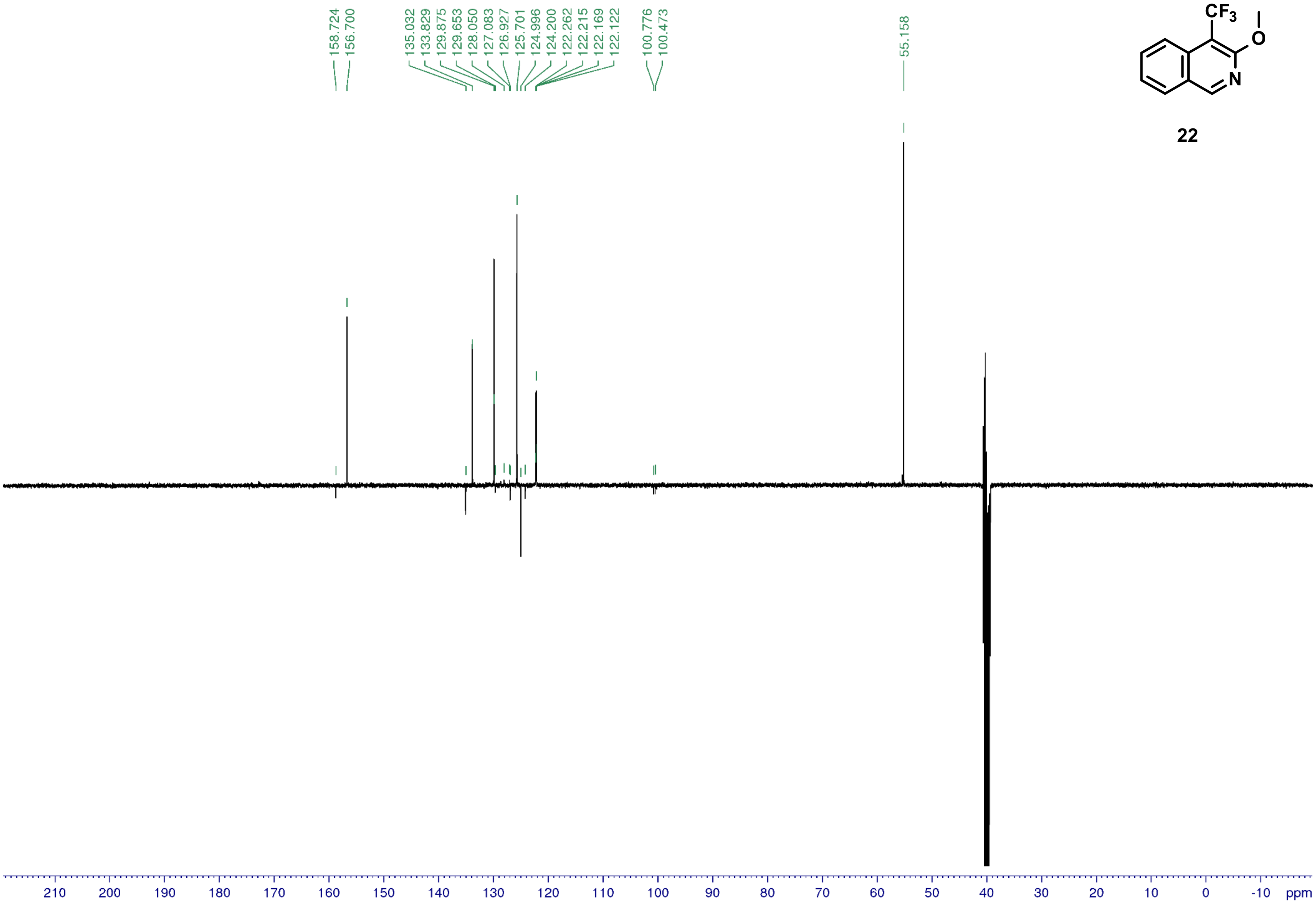
158.724
156.700
135.032
133.829
129.875
129.653
128.050
127.083
126.927
125.701
124.996
124.200
122.262
122.215
122.169
122.122
100.776
100.473
55.158
CF3
O
N
210
200
190
180
170
160
150
140
130
120
110
100
90
80
70
60
50
40
30
20
10
0
-10
ppm

22

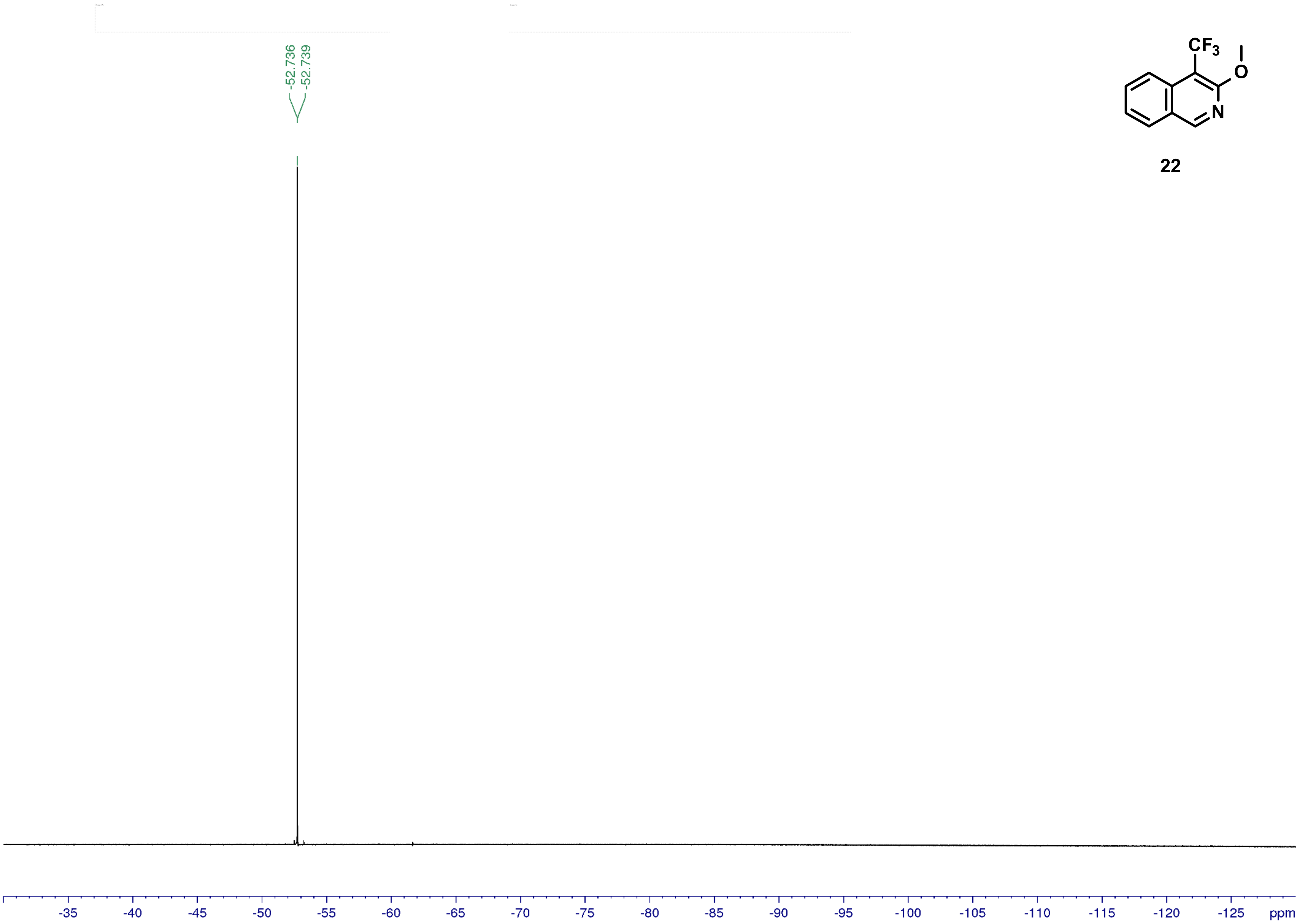
-52.736
-52.739
CF3
O
N
-35
-40
-45
-50
-55
-60
-65
-70
-75
-80
-85
-90
-95
-100
-105
-110
-115
-120
-125
ppm

22

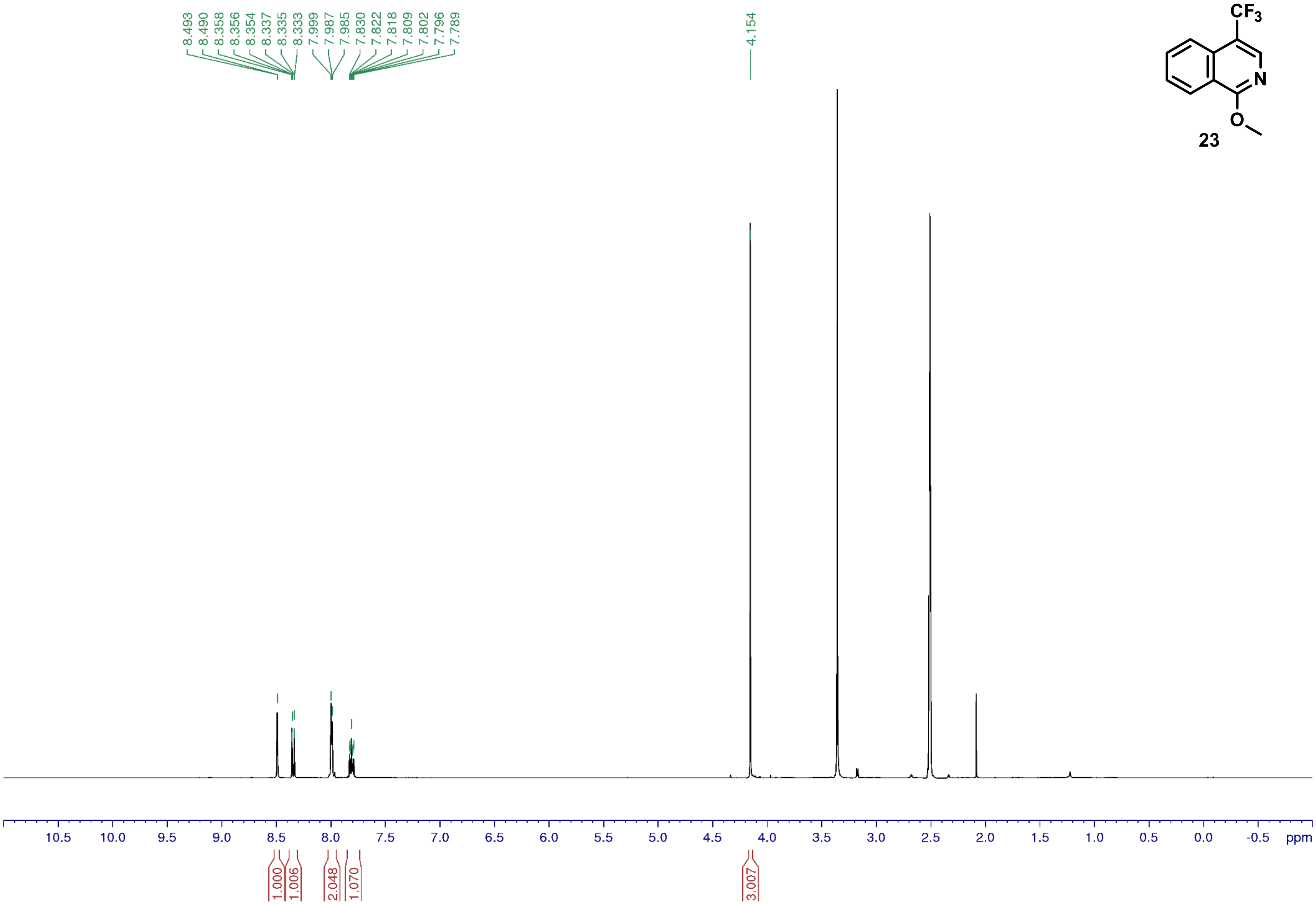

8.493
8.490
8.358
8.356
8.354
8.337
8.335
8.333
7.999
7.987
7.985
7.830
7.822
7.818
7.809
7.802
7.796
7.789
4.154
10.5
10.0
9.5
9.0
8.5
8.0
7.5
7.0
6.5
6.0
5.5
5.0
4.5
4.0
3.5
3.0
2.5
2.0
1.5
1.0
0.5
0.0
-0.5
ppm
1.000
1.006
2.048
1.070
3.007
CF3
N
O

23

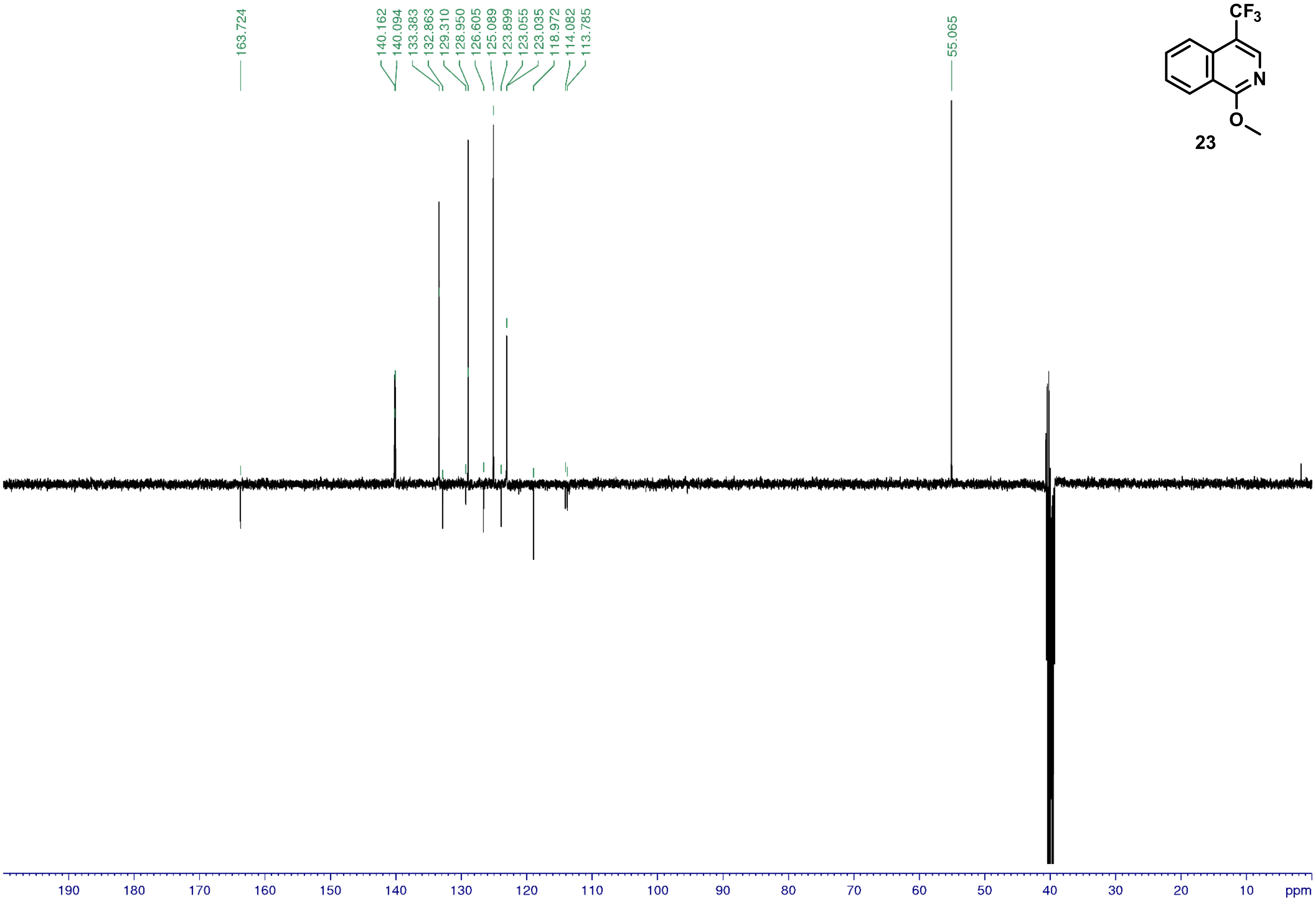

163.724
140.162
140.094
133.383
132.863
129.310
128.950
126.605
125.089
123.899
123.055
123.035
118.972
114.082
113.785
55.065
CF3
N
O
23
190
180
170
160
150
140
130
120
110
100
90
80
70
60
50
40
30
20
10
ppm

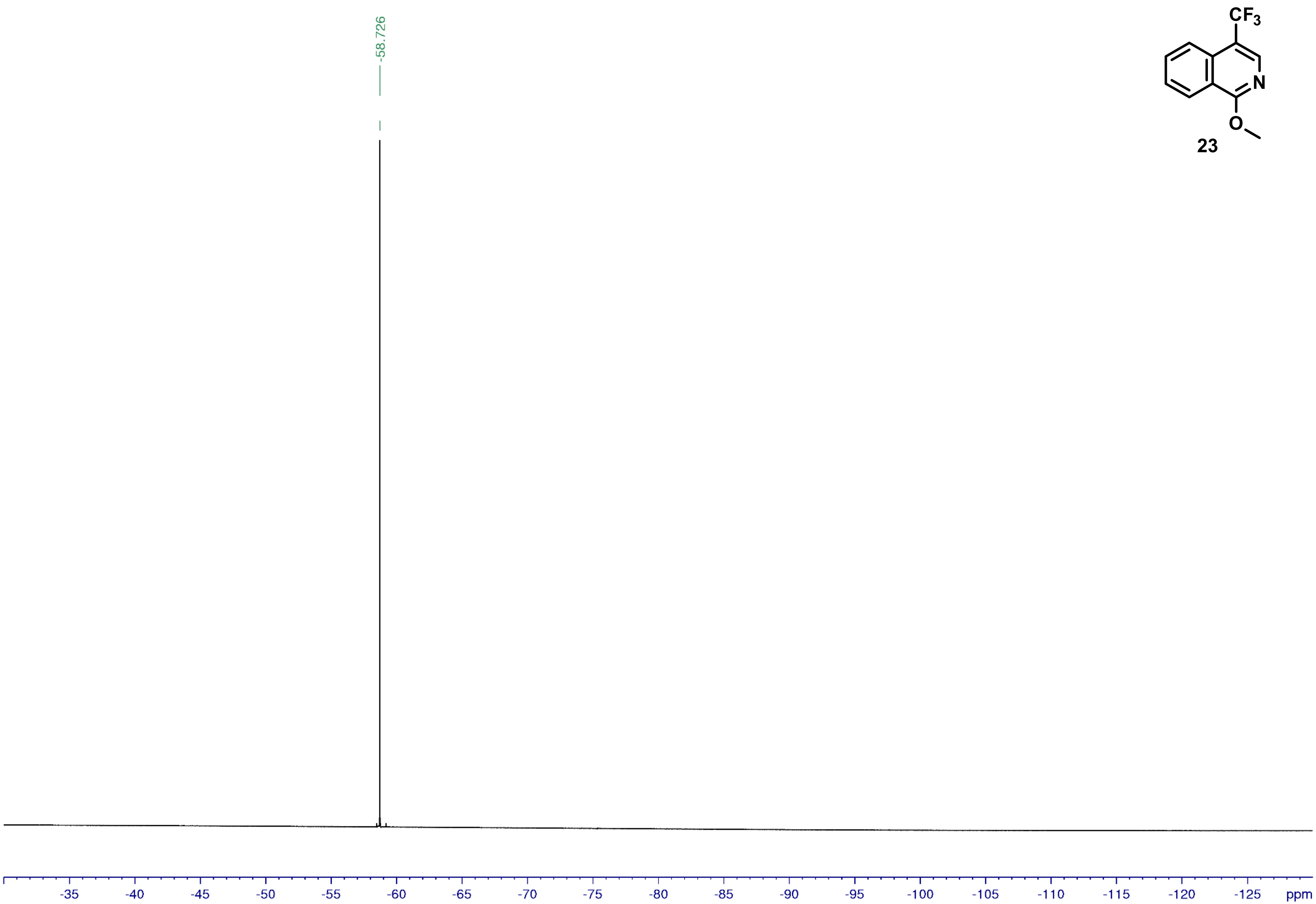
-58.726
-35
-40
-45
-50
-55
-60
-65
-70
-75
-80
-85
-90
-95
-100
-105
-110
-115
-120
-125
ppm
CF3
N
O

23

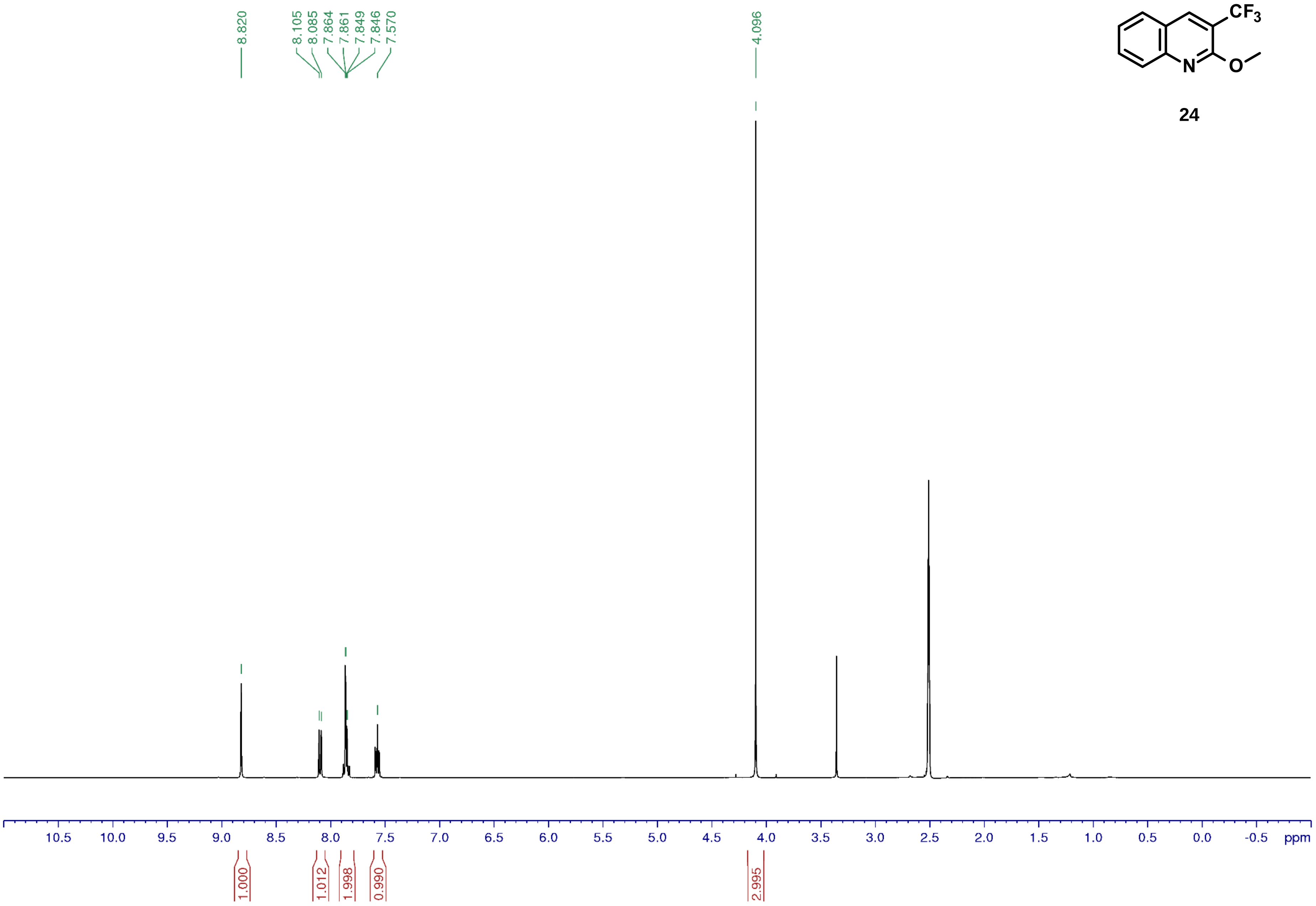

8.820
8.105
8.085
7.864
7.861
7.849
7.846
7.570
4.096
10.5
10.0
9.5
9.0
8.5
8.0
7.5
7.0
6.5
6.0
5.5
5.0
4.5
4.0
3.5
3.0
2.5
2.0
1.5
1.0
0.5
0.0
-0.5
ppm
1.000
1.012
1.998
0.990
2.995
CF3
N
O

24

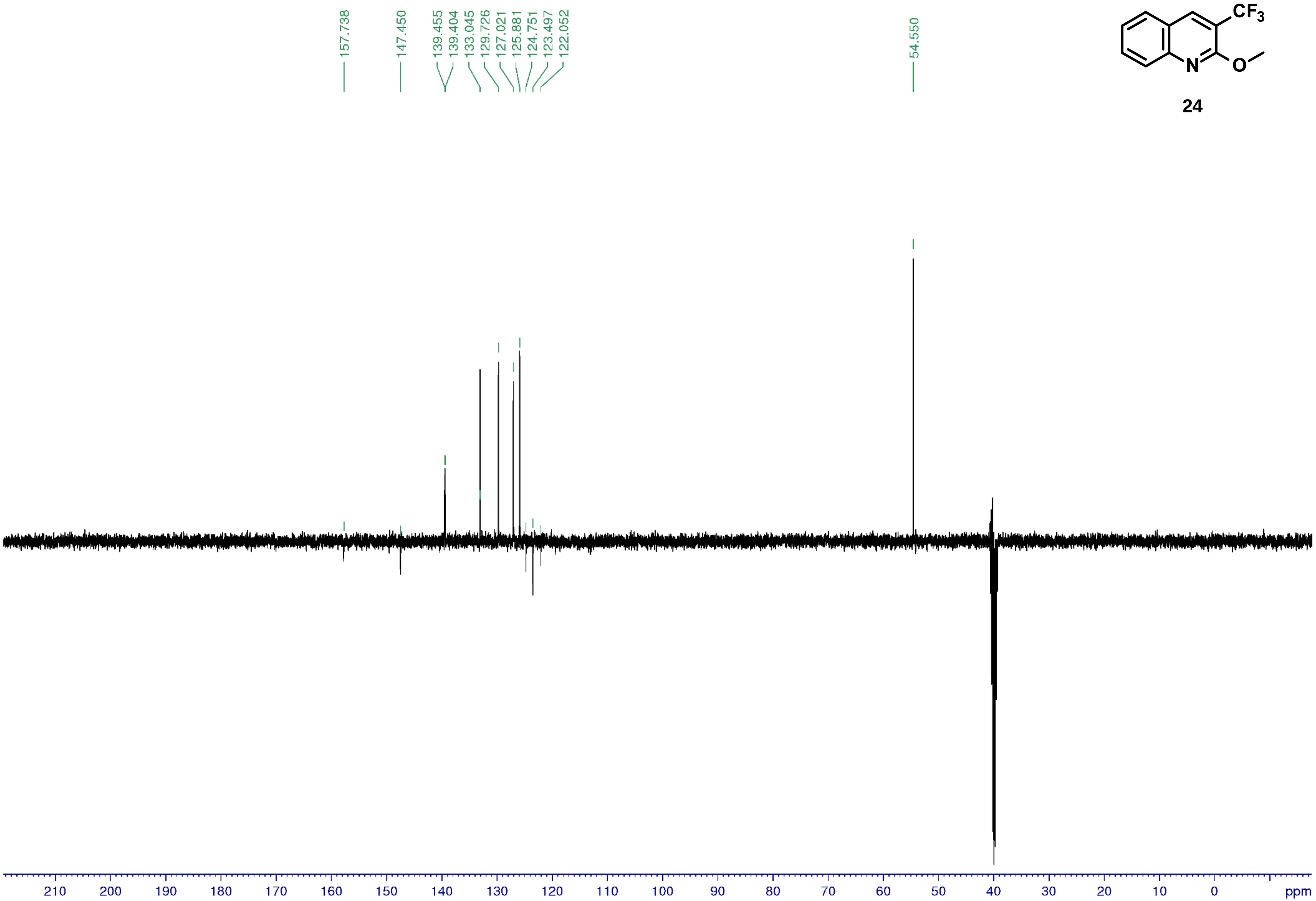
157.738
147.450
139.455
139.404
133.045
129.726
127.021
125.881
124.751
123.497
122.052
54.550
210 200 190 180 170 160 150 140 130 120 110 100 90 80 70 60 50 40 30 20 10 0 ppm
CF3
N
O

24

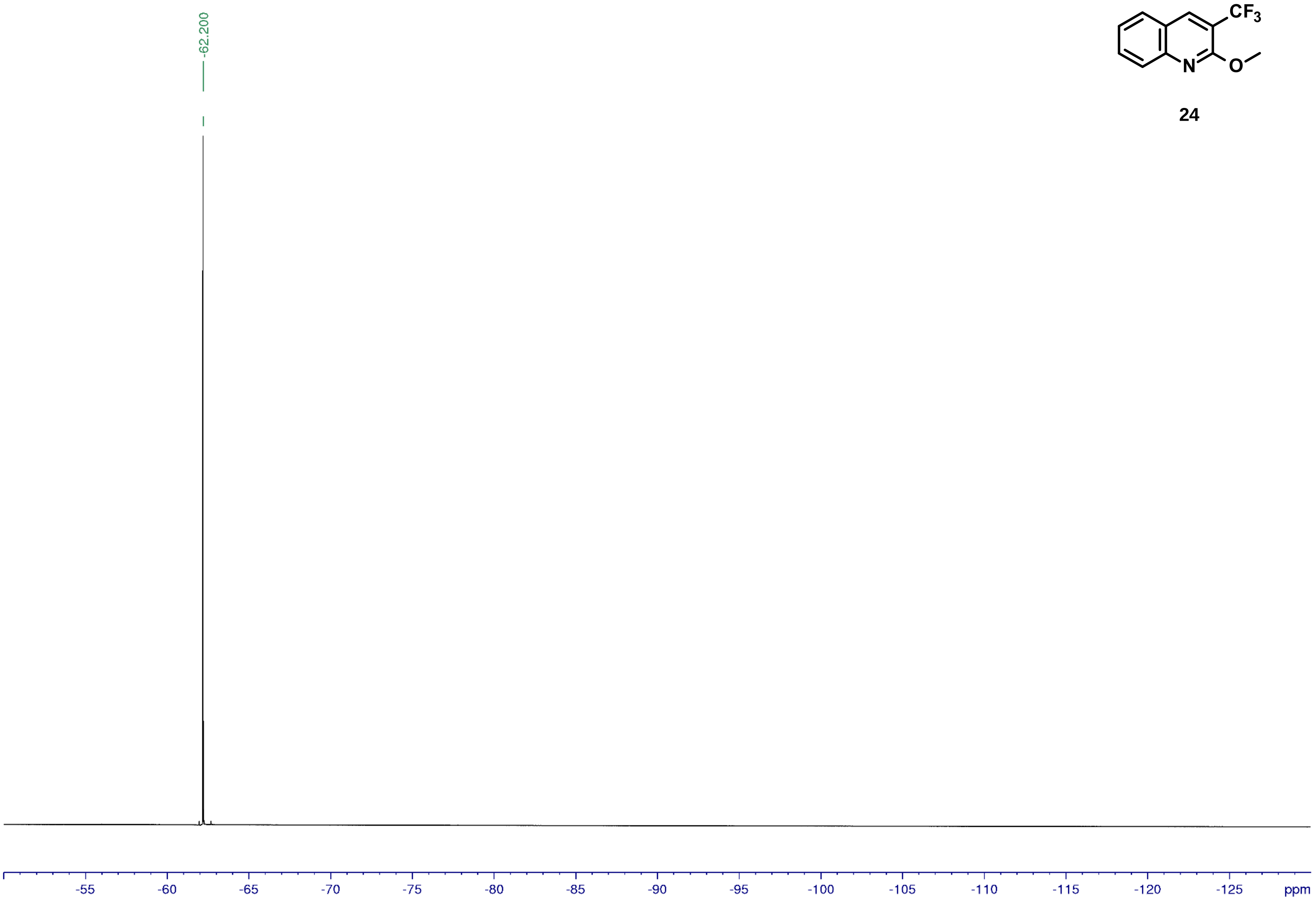
-62.200
CF3
N
O
24
-55
-60
-65
-70
-75
-80
-85
-90
-95
-100
-105
-110
-115
-120
-125
ppm

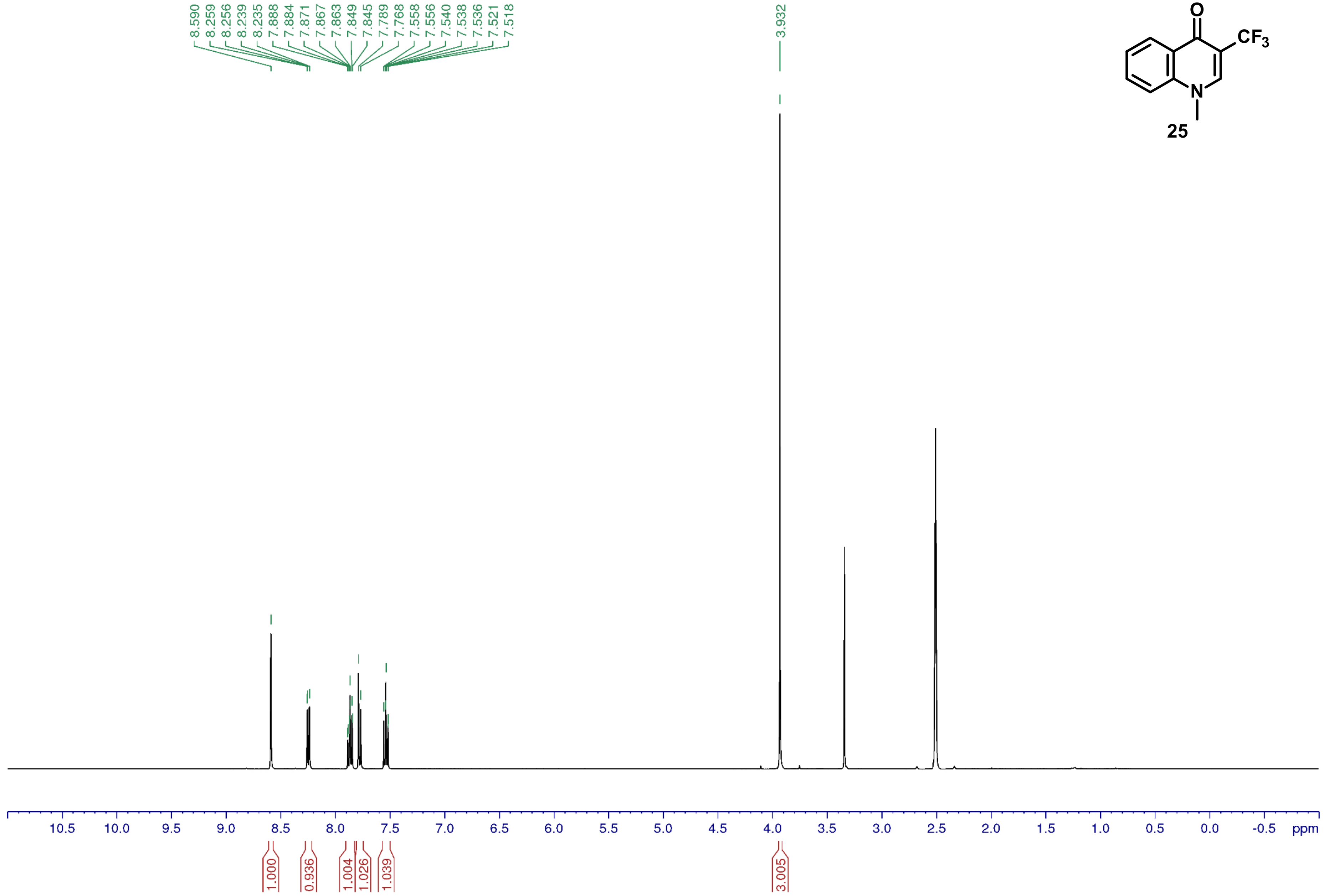
8.590
8.259
8.256
8.239
8.235
7.888
7.884
7.871
7.867
7.863
7.849
7.845
7.789
7.768
7.558
7.556
7.540
7.538
7.536
7.521
7.518
3.932
10.5 10.0 9.5 9.0 8.5 8.0 7.5 7.0 6.5 6.0 5.5 5.0 4.5 4.0 3.5 3.0 2.5 2.0 1.5 1.0 0.5 0.0 -0.5 ppm
1.000
0.936
1.004
1.026
1.039
3.005
O
CF3
N

25

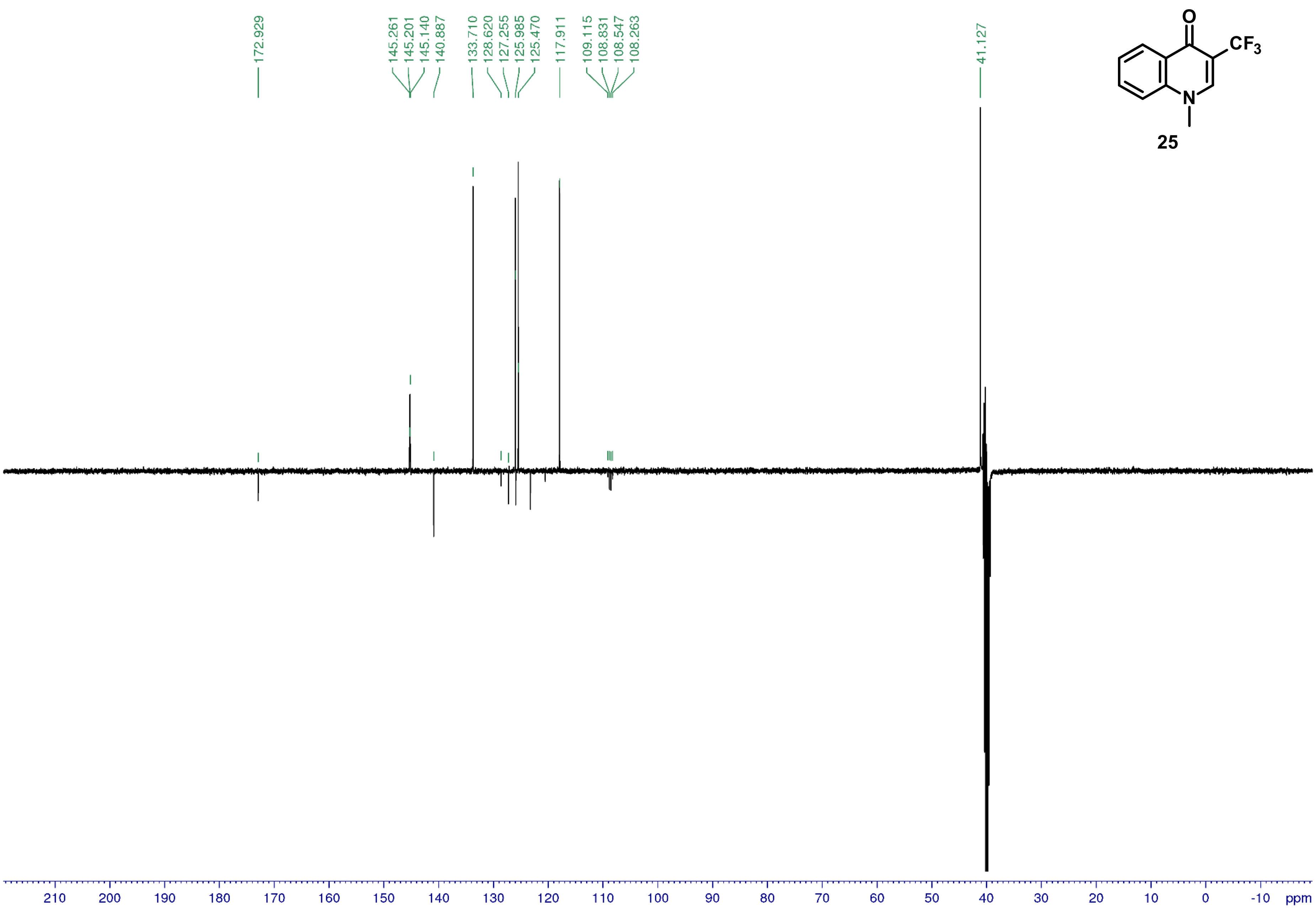
172.929
145.261
145.201
145.140
140.887
133.710
128.620
127.255
125.985
125.470
117.911
109.115
108.831
108.547
108.263
41.127
O
CF3
N
25
210 200 190 180 170 160 150 140 130 120 110 100 90 80 70 60 50 40 30 20 10 0 -10 ppm

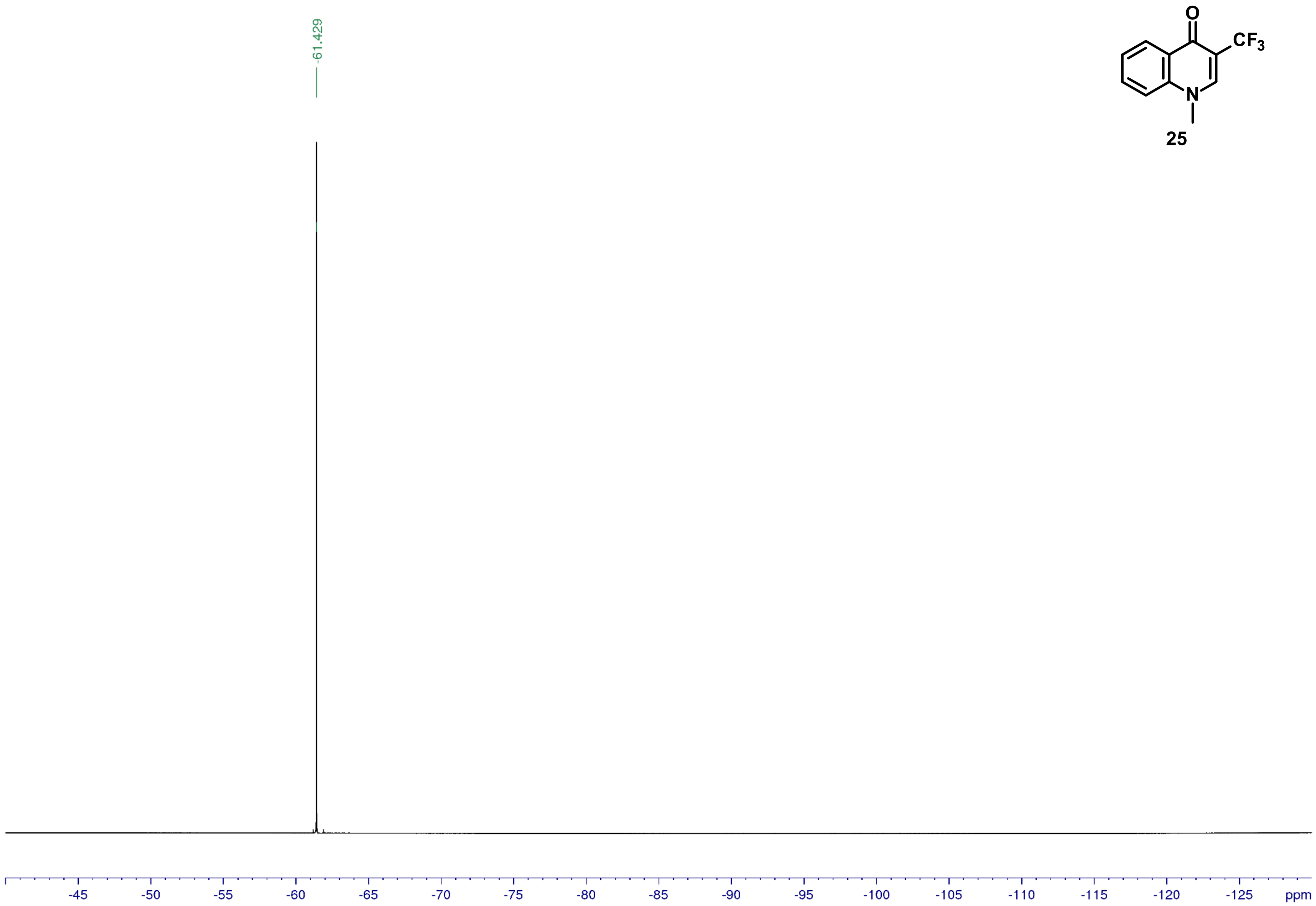
25
-61.429
-45
-50
-55
-60
-65
-70
-75
-80
-85
-90
-95
-100
-105
-110
-115
-120
-125
ppm
